# Anti‐Hofmeister Anion Selectivity via a Mechanical Bond Effect in Neutral Halogen‐Bonding [2]Rotaxanes

**DOI:** 10.1002/anie.202214523

**Published:** 2022-11-17

**Authors:** Andrew Docker, Yuen Cheong Tse, Hui Min Tay, Andrew J. Taylor, Zongyao Zhang, Paul D. Beer

**Affiliations:** ^1^ Department of Chemistry University of Oxford Chemistry Research Laboratory 12 Mansfield Road Oxford OX1 3TA UK

**Keywords:** Anion Recognition, Anti-Hofmeister Bias, Halogen Bonding (XB), Mechanically Interlocked Molecules, Rotaxanes

## Abstract

Exceptionally strong halogen bonding (XB) donor‐chloride interactions are exploited for the chloride anion template synthesis of neutral XB [2]rotaxane host systems which contain perfluoroaryl‐functionalised axle components, including a remarkably potent novel 4,6‐dinitro‐1,3‐bis‐iodotriazole motif. Halide anion recognition properties in aqueous‐organic media, determined via extensive ^1^H NMR halide anion titration experiments, reveal the rotaxane host systems exhibit dramatically enhanced affinities for hydrophilic Cl^−^ and Br^−^, but conversely diminished affinities for hydrophobic I^−^, relative to their non‐interlocked axle counterparts. Crucially, this mechanical bond effect induces a binding selectivity which directly opposes Hofmeister bias. Free‐energy analysis of this mechanical bond enhancement demonstrates anion recognition by neutral XB interlocked host systems as a rare and general strategy to engineer anti‐Hofmeister bias anion selectivity in synthetic receptor design.

## Graphical abstract

Neutral XB [2]rotaxanes containing remarkably potent XB axle components including a novel 4,6‐dinitro‐1,3‐bis‐iodotriazole motif are constructed via a discrete chloride anion template directed synthesis. Quantitative ^1^H NMR halide anion titration studies in competitive aqueous‐organic solvent mixtures reveal the XB [2]rotaxanes display pronounced anti‐Hofmeister halide anion recognition selectivity for chloride over iodide.

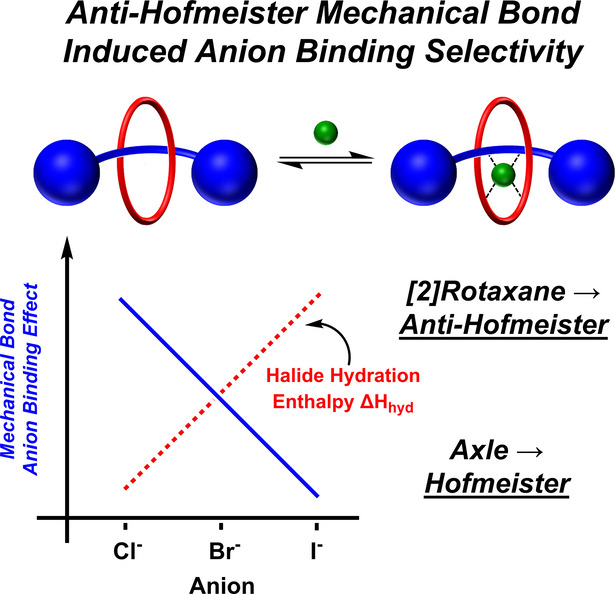

## Introduction

The pervasiveness of anions in a wide range of biological, environmental and industrial processes has continued to stimulate interest in the development of synthetic receptor systems capable of their strong and selective recognition.[^1^, ^2^] Our research endeavours have focused on exploiting the cavities of interlocked molecules (e.g. rotaxanes and catenanes) as unique preorganised coordination environments for the purposes of anion recognition and sensing.[^3^, ^4^] Indeed, we have demonstrated that mechanically interlocked host frameworks, in addition to possessing prodigious anion binding affinities, provide a powerful strategy to augment anion selectivity profiles relative to non‐interlocked systems.[^5^, ^6^] Despite the enormous progress that has been made in the anion recognition field, a persistent challenge is developing selective receptor systems capable of functioning in aqueous media[^7^, ^8^] wherein traditional non‐covalent interactions employed for anion binding such as hydrogen bonding (HB) are diminished.^[9]^ While highly positively charge assisted receptors typically exhibit enhanced anion affinities relative to neutral counterparts, the reliance on electrostatic interactions to bind anions generally elicits poor selectivity. To address this challenge, our group has sought to exploit potent, neutral, sigma (σ)‐hole‐based interactions such as halogen (XB), chalcogen (ChB) and pnictogen (PnB) bonding for the purposes of molecular ion recognition.[^10^, ^11^, ^12^, ^13^, ^14^, ^15^, ^16^, ^17^, ^18^] XB in particular has emerged as a powerful addition to the arsenal of non‐covalent interactions to achieve anion binding,[^19^, ^20^, ^21^, ^22^, ^23^, ^24^, ^25^] frequently exhibiting dramatically enhanced affinities relative to HB analogues, even in competitive aqueous media.[^26^, ^27^, ^28^, ^29^] However, extensive anion solvation in aqueous media almost invariably confers so‐called “Hofmeister bias”,[^30^, ^31^, ^32^] wherein anion binding selectivity profiles are dictated by hydration enthalpies, i.e. the less solvated the anion the more strongly it is bound.^[33]^ Considering that the majority of biologically relevant anions are strongly hydrophilic e.g. chloride, the predominant anionic electrolyte underpinning many crucial biological functions, strategies to mitigate Hofmeister bias would constitute a considerable leap forwards in the field of supramolecular anion host–guest recognition. Examples of synthetic receptors exhibiting anti‐Hofmeister anion selectivity however, are very rare, with the few reported examples typically relying on size discrimination or subtle anion induced aggregation effects.[^41^, ^42^, ^43^, ^44^, ^45^] Combining the advantages of mechanically bonded interlocked host topologies and highly potent XB donor motifs, herein we importantly demonstrate neutral XB [2]rotaxanes capable of anti‐Hofmeister halide anion recognition in water containing solvent media. This is achieved through the axle component integration of perfluoroaryl‐functionalised XB donors, including a remarkably potent novel 4,6‐dinitro‐1,3‐bis‐iodotriazole motif which facilitate unprecedented XB directed discrete chloride anion template assembly of the neutral [2]rotaxane host systems. Extensive quantitative ^1^H NMR halide anion titration studies in highly competitive aqueous‐organic solvent mixtures reveal that the mechanically bonded [2]rotaxanes exhibit pronounced selectivity for hydrophilic chloride over hydrophobic iodide i.e. anti‐Hofmeister bias. Crucially, quantitative free energy analysis reveals a general mechanical bond effect which directly opposes Hofmeister bias, thereby constituting a rare strategy to engineer anti‐Hofmeister anion selectivity behaviour.

## Results and Discussion

### Design and synthesis of acyclic receptor systems

**Scheme 1:**
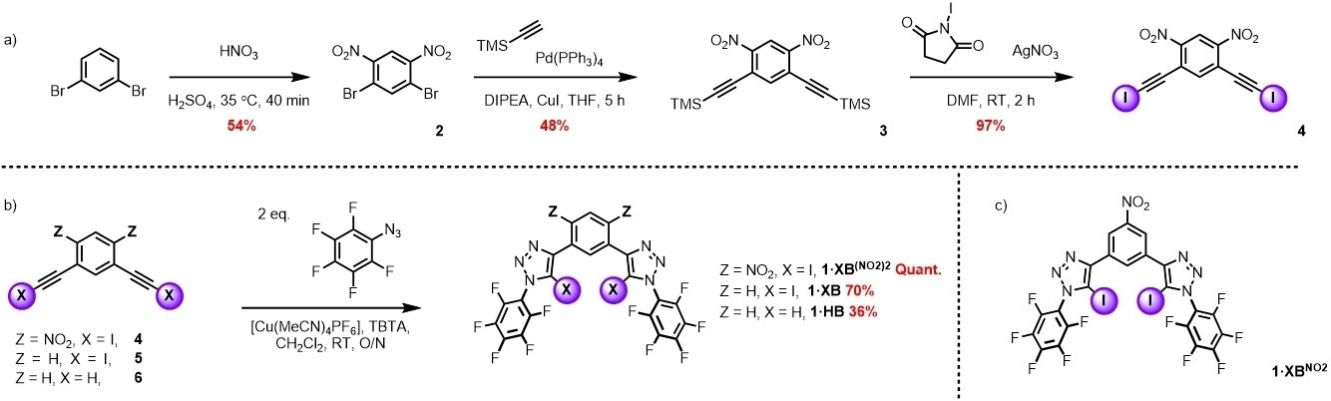
a) Synthesis of 4,6‐dinitro‐1,3‐bis‐iodoalkyne. b) Synthesis of XB and HB acyclic receptors **1** ⋅ **XB**, **1** ⋅ **XB^(NO2)2^** and **1** ⋅ **HB** via a CuAAC methodology. c) Chemical structure of a previously reported XB receptor; **1** ⋅ **XB^NO2^**.^[18]^

Given reports that XB donor potency is highly sensitive to local electronic environments and is dramatically influenced by proximal electron‐deficient functional groups,[^17^, ^27^] we sought to explore a new 1,3‐bis‐iodotriazole motif wherein the central aromatic scaffold is further decorated with strongly electron‐withdrawing groups. Taking into account the nitro (−NO_2_) substituent possesses one of the largest positive Hammett substituent constants for a neutral functional group (σ_p_=+0.778), a new 4,6‐dinitro‐1,3‐bis‐iodotriazole system was targeted, the synthesis of which is shown Scheme 1a. Firstly, 1,3‐dibromobenzene was subjected to nitration with fuming HNO_3_ and conc. H_2_SO_4_ to give 1,3‐dibromo‐4,6‐dinitrobenzne **2** in 54 % yield.^[47]^ A Sonagashira coupling reaction between **2** and TMS‐acetylene in the presence of Pd(PPh_3_)_4_ and CuI catalysts under a strictly controlled reaction time of 5 hours gave the bis‐TMS‐protected alkyne **3** in 48 % yield after purification by column chromatography. Treatment of **3** with N‐iodosuccinimide (NIS) in the presence of a catalytic amount of AgNO_3_ afforded, via an in situ TMS deprotection and silver acetylide formation, the bis‐iodoalkyne **4** quantitatively. The synthesis of the target acyclic receptor **1** ⋅ **XB^(NO2)2^** was achieved through a Cu^I^ azide‐alkyne cycloaddition (CuAAC) reaction between **4** and 2 equivalents of pentafluorophenyl azide, in the presence of catalytic [Cu(MeCN)_4_]PF_6_ and the rate accelerating ligand TBTA in dichloromethane, which after aqueous work up procedures and purification by column chromatography gave **1** ⋅ **XB^(NO2)2^** in excellent yield (Scheme 1b). To elucidate the effect of nitro group incorporation and the role of XB in the anion binding properties of the system, the XB 1,3‐bis‐iodotriazole benzene analogue **1** ⋅ **XB** and its HB bis‐prototriazole analogue **1** ⋅ **HB** were also synthesised in a similar fashion from the corresponding bis‐iodo and ‐proto alkynes of 1,3‐diethynyl benzene, **5** and **6** respectively and isolated in respective yields of 70% and 36% (Scheme 1b).

### Chloride anion binding studies of the acyclic receptor series

**Figure 1:**
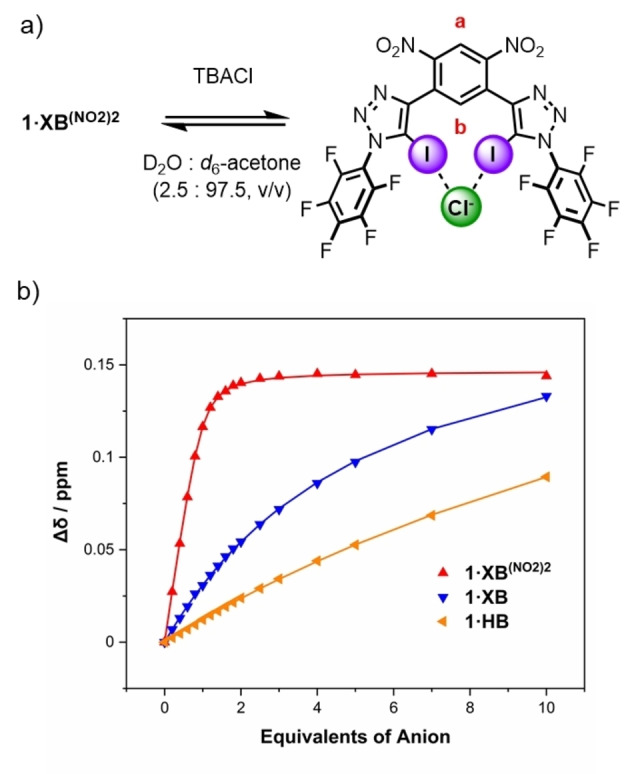
a) Chloride anion binding equilibrium of **1** ⋅ **XB^(NO2)2^**. b) Anion binding titration isotherm for **1** ⋅ **XB^(NO2)2^**, **1** ⋅ **XB** and **1** ⋅ **HB**, where triangles represent experimental data and the lines represent the fitted isotherm.

To firstly establish a relative scale for the anion binding potency of the XB receptors **1** ⋅ **XB^(NO2)2^**, **1** ⋅ **XB** and HB receptor, **1** ⋅ **HB**, preliminary ^1^H NMR spectroscopic chloride anion titration experiments were conducted in D_2_O : acetone‐d_6_ (2.5 : 97.5, v/v). In general, the addition of increasing equivalents of chloride, added as its tetrabutylammonium (TBA) salt, to solutions of the receptors induced significant chemical shift perturbations in multiple proton resonances. In the case of the XB receptors, **1** ⋅ **XB^(NO2)2^** and **1** ⋅ **XB**, the most significant downfield shifts were observed for the proton signal corresponding to the environment ortho to both iodo‐triazoles, in the case of **1** ⋅ **XB^(NO2)2^** proton signal b, consistent with XB mediated Cl^−^ binding, a representative example for **1** ⋅ **XB^(NO2)2^** is shown in Figure 1a. Similar observations were made in the corresponding signal of **1** ⋅ **HB** with concomitant downfield perturbations in the chemical shift of the triazole C−H providing evidence for the anion binding events occurring in the respective bis‐triazole clefts. Monitoring these chemical shift perturbations as a function of chloride concentration generated isotherm binding titration data (Figure 1b), which was analysed by Bindfit^[48]^ and generated 1 : 1 host:guest stoichiometric chloride association constants (*K*_a_) for the series (Table 1).

**Table 1:** Chloride anion association constants (*K*_a_) for the acyclic receptor series in D_2_O : acetone‐d_6_ (2.5 : 97.5, v/v).

	Chloride association constants (*K*_a_/M^−1^)^[a]^
**1** ⋅ **XB** ^[b]^	195 (1)
**1** ⋅ **XB^NO2^**	6 070 (343)
**1** ⋅ **XB^(NO2)2^** ^[b]^	9 650 (518)
**1** ⋅ **HB** ^[c]^	23 (1)

[a] Chloride added as the TBA salt. Errors (±) are in parentheses. [b] Fitted from perturbations of the internal aromatic proton signal. [c] Fitted from perturbations of the triazole proton signal.

Upon surveying the summarised *K*_a_ values, in Table 1, it is notable that incorporation of electron‐withdrawing nitro groups into the central aromatic scaffold is accompanied by a dramatic increase in chloride affinity, such that **1** ⋅ **XB^(NO2)2^** possesses a *K*_a_(Cl^−^) value 50 times larger than that of **1** ⋅ **XB**, and this dinitro motif even outperforms a previously reported mono‐nitrated analogue, **1** ⋅ **XB^NO2^**.[^18^, ^49^] Importantly, the potency of the XB interactions in the anion affinity is underscored by the HB analogue, **1** ⋅ **HB**, which exhibits a considerably weaker chloride affinity with a modest *K*_a_ of 23 M^−1^.

**Figure 2:**
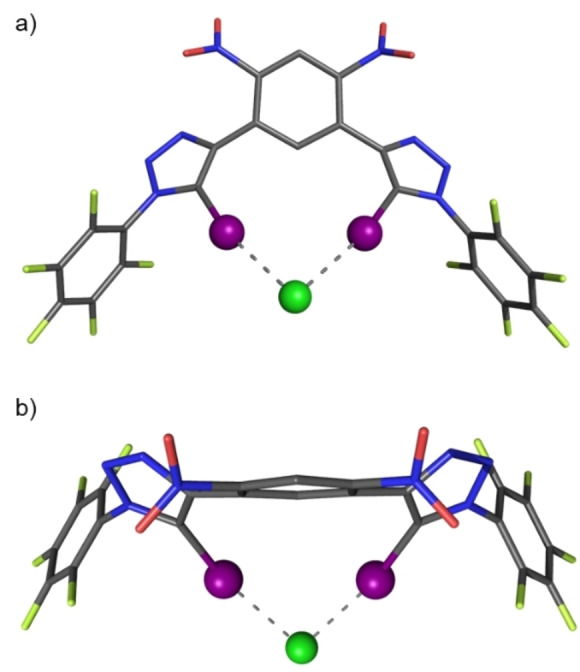
X‐ray crystal structure of **1** ⋅ **XB^(NO2)2^** ⋅ TBACl complex; a) front view; b) top view. TBA countercation and hydrogens omitted for clarity. Grey=carbon, light green=fluorine, dark green=chlorine, blue=nitrogen, red=oxygen, purple=iodine.

Further evidence for the postulated chloride binding mode of **1** ⋅ **XB^(NO2)2^** was obtained through X‐ray diffraction analysis of single crystals grown from equimolar mixtures of **1** ⋅ **XB^(NO2)2^** and TBACl in CH_2_Cl_2_. The determined structure confirms the formation of a bidentate XB‐anion coordination mode through bifurcated C−I⋅⋅⋅Cl^−^ contacts (Figure 2). Indeed, the potency of these interactions, alluded to by solution phase studies, is evidenced by the remarkably short iodine⋅⋅⋅chlorine distances of 3.075 Å and 3.099 Å which corresponds to contractions of approximately 80 % relative to their van der Waals radii.

### Axle and [2]rotaxane synthesis

**Figure 3:**
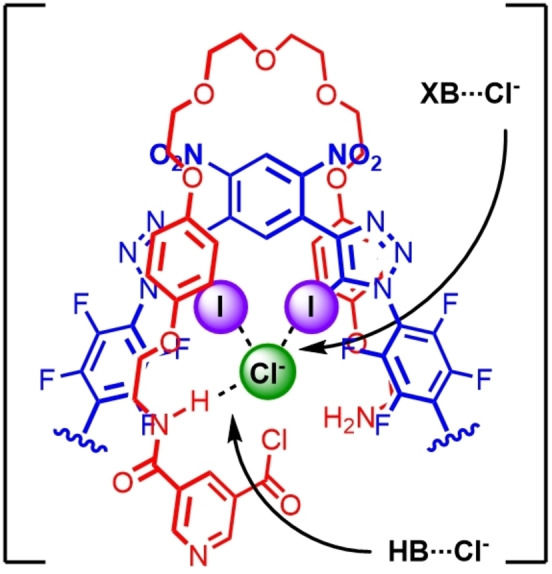
Proposed structure of an intermediate in a discrete XB and HB chloride anion templated amide condensation methodology for the synthesis of a neutral [2]rotaxane.

Motivated by the impressive chloride binding of the acyclic receptors; **1** ⋅ **XB** and **1** ⋅ **XB^(NO2)2^**, we sought to investigate whether these *neutral* motifs could be employed in our previously developed discrete anion template directed amide condensation methodology for the synthesis of cationic [2]rotaxanes using a positively charged HB donor 3,5‐bis‐amide pyridinium axle component.^[50]^ It was envisaged that integration of these potent XB motifs in axle components would bind a chloride template with sufficient affinity through complementary XB⋅⋅⋅Cl^−^ and HB⋅⋅⋅Cl^−^ anion interactions, as to facilitate an intramolecular “clipping” reaction around the axle component to access neutral [2]rotaxane formation (Figure 3).

**Scheme 2:**
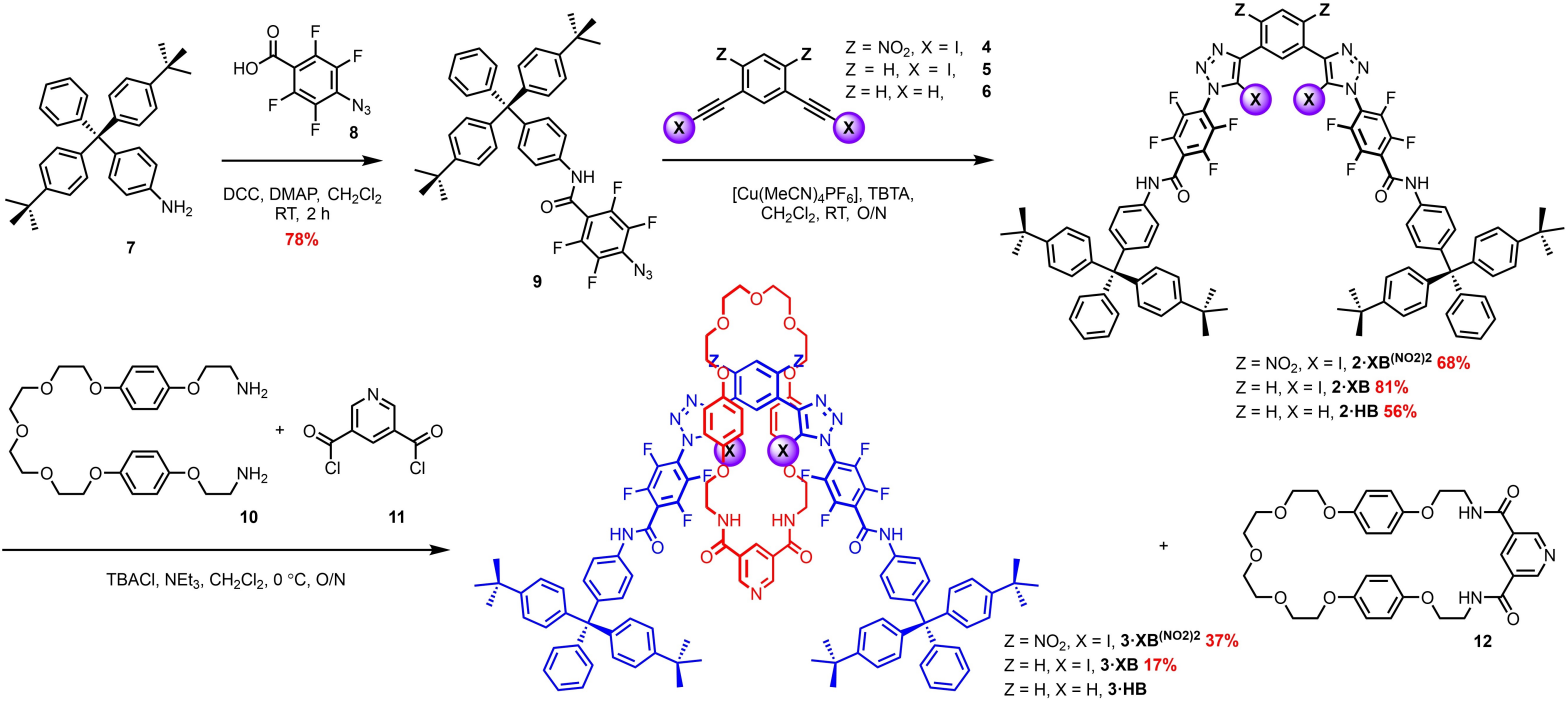
Axle and [2]rotaxane synthesis via a CuAAC methodology or a chloride anion template directed amide condensation reaction respectively.

For the synthesis of the targeted XB axle components, perfluoroaryl azide functionalised stopper precursor **9** was obtained via a DCC promoted amide coupling between aniline stopper **7** ^[5]^ and perfluorinated acid **8** ^[51]^ in the presence of catalytic DMAP to afford **9** in 78 % yield (Scheme 2). The target axles, **2** ⋅ **XB^(NO2)2^**, **2** ⋅ **XB** and **2** ⋅ **HB**, were obtained through CuAAC reactions between **9** and the appropriately functionalised bis‐iodoalkynes **4** and **5** or bis‐protoalkyne **6**, in yields of 68 %, 81 % and 56 % respectively.

With the series of XB and HB axles in hand, attention was directed towards the synthesis of the corresponding [2]rotaxanes. In a typical procedure, the axle was dissolved in an anhydrous CH_2_Cl_2_ solution also containing one equivalent of the Cl^−^ anion template agent as its TBA salt, and an excess of the bis‐amine macrocycle precursor **10** and NEt_3_ at 0 °C. This was followed by the addition of freshly generated bis‐acid chloride **11**, which was prepared from the corresponding dicarboxylic acid and oxalyl chloride. In the case of **2** ⋅ **XB^(NO2)2^** and **2** ⋅ **XB**, after 12 hours ESI‐MS analysis of the crude reaction mixtures revealed *m*/*z* peaks at 2392 and 2300 Da, corresponding to the target XB neutral [2]rotaxanes.

**Figure 4:**
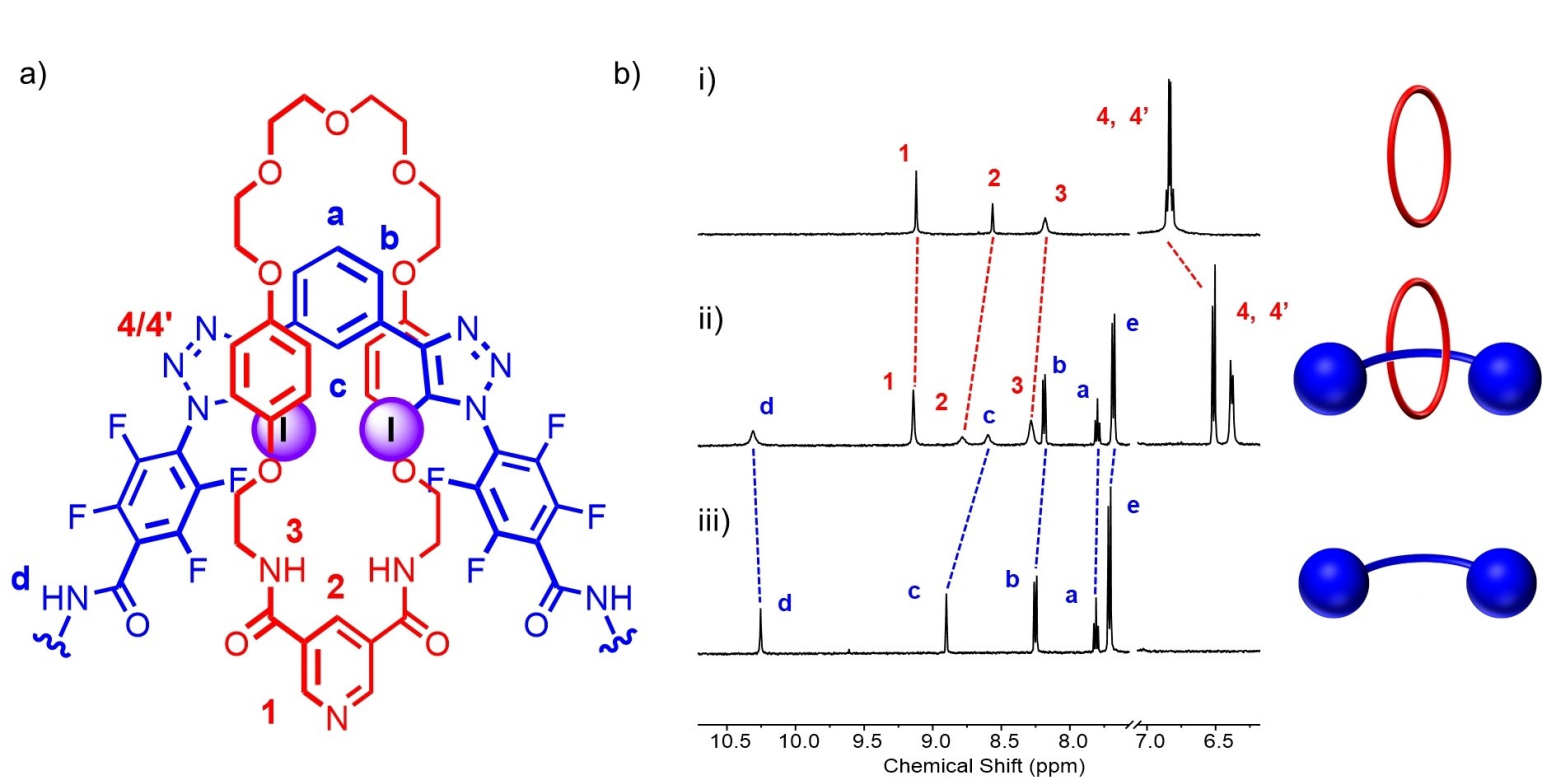
a) Truncated chemical structure of **3** ⋅ **XB**. b) Truncated stacked ^1^H NMR spectra of i) macrocycle **12**, ii) [2]rotaxane **3** ⋅ **XB** and iii) axle **2** ⋅ **XB** (acetone‐d_6_, 500 MHz, 298 K).

After aqueous work up procedures, purification of the crude reaction mixtures by preparative thin layer chromatography enabled the isolation of the target [2]rotaxanes **3** ⋅ **XB^(NO2)2^** and **3** ⋅ **XB** in 37 % and 17 % yields respectively, in addition to ring closed macrocycle **12**. Considering the *K*_a_(Cl^−^) values of the model XB acyclic systems (Table 1), the isolated yields are clearly correlated with chloride template affinity by the central XB donor motif. This is underscored by the fact that under identical reaction conditions no evidence for the formation of the interlocked HB [2]rotaxane product, **3** ⋅ **HB**, was observed when using the HB axle, **2** ⋅ **HB**, highlighting the crucial role of potent XB C−I⋅⋅⋅Cl^−^ interactions to template the formation of the [2]rotaxanes. The novel XB [2]rotaxanes **3** ⋅ **XB^(NO2)2^** and **3** ⋅ **XB** were characterised by ^1^H, ^19^F, ^13^C NMR spectroscopies and high‐resolution mass spectrometry. Evidence for the interlocked nature of the products is shown through the comparison of ^1^H NMR spectra of the [2]rotaxane, the respective axle components and macrocycle **12** (a representative example for **3** ⋅ **XB** is shown in Figure 4). The largest chemical shift perturbations relative to their non‐interlocked components, in addition to pronounced broadening, are those associated with the interior of the [2]rotaxane's cavity, namely the benzene axle proton c and pyridine macrocycle proton 2. Furthermore, the macrocycle hydroquinone resonances 4 and 4′ split and move significantly upfield (Δδ ≈0.3 ppm), which is rationalised by the formation of favourable donor‐acceptor type interactions between the electronic rich aryl groups of the macrocycle and the electron‐deficient axle component. Similar observations were noted in the case of **3** ⋅ **XB^(NO2)2^** (Figure S38). Further confirmation for the interlocked nature of **3** ⋅ **XB^(NO2)2^** and **3** ⋅ **XB** was also provided by 2D ^1^H‐^1^H ROESY NMR spectroscopy (Figures S76 and S77). It is noteworthy that in the context of anion template synthesis of interlocked molecules, this constitutes a considerable milestone. The overwhelming majority of examples to date feature at least one positively charged component to facilitate assembly, such that **3** ⋅ **XB^(NO2)2^** and **3** ⋅ **XB** are not only rare examples of neutral mechanically interlocked molecules constructed by discrete anion templation,[^44^, ^52^] but the first to exploit XB as the principal templating interaction.

### Halide anion binding studies of the axles and [2]rotaxanes

**Figure 5:**
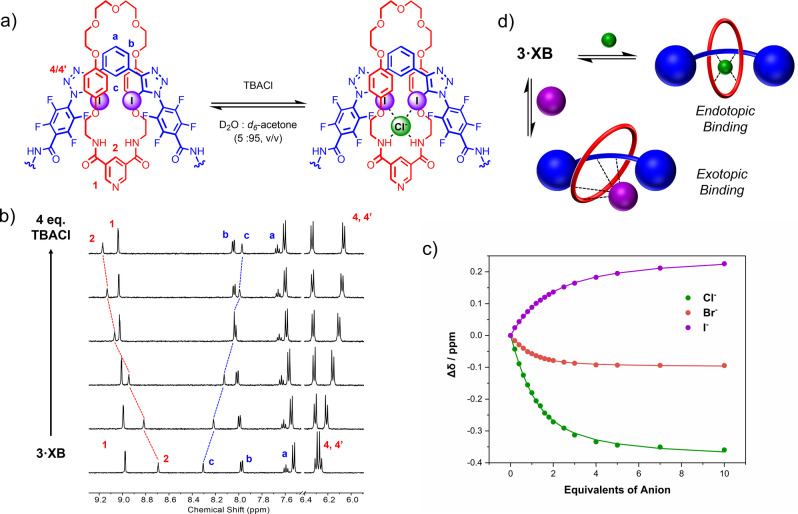
a) Truncated chemical structure and chloride binding equilibrium of **3** ⋅ **XB**. b) Stacked truncated ^1^H NMR spectra of the **3** ⋅ **XB** titration with TBACl (D_2_O : acetone‐d_6_ (5 : 95, v/v), 500 MHz, 298 K). c) Anion binding isotherms for **3** ⋅ **XB** and the halides where circles represent experimental data and the lines represent the fitted isotherm. d) Cartoon representation of rotaxane endotopic binding mode of chloride and bromide (represented by a green sphere), and exotopic iodide binding mode (represented by a purple sphere).

The halide anion recognition properties of neutral [2]rotaxanes **3** ⋅ **XB^(NO2)2^** and **3** ⋅ **XB**, and their respective non‐interlocked axle components **2** ⋅ **XB^(NO2)2^** and **2** ⋅ **XB**, were initially investigated in the competitive aqueous‐organic solvent mixture D_2_O : acetone‐d_6_ (5 : 95, v/v), via ^1^H NMR anion titration experiments. In a typical experiment the addition of either chloride, bromide or iodide, as their TBA salts, to solutions of the [2]rotaxanes or axles induced significant perturbations in XB binding cleft signals, as well as changes in the pyridine resonances of the macrocyclic components for the interlocked hosts. Interestingly, in the case of [2]rotaxane **3** ⋅ **XB**, anion titration experiments conducted with chloride and bromide induced an upfield shift of axle proton signal **c** and a downfield shift of macrocycle signal **2** (Figure 5a–c), (for further discussion regarding perturbations observed during anion titration experiments see Supporting Information page S39). However, upon the addition of iodide the direction of proton chemical shift perturbations observed for Cl^−^ and Br^−^ was reversed i.e., downfield shifts of **c** and upfield shifts of **2**. Bindfit analysis of the titration data in general determined 1 : 1 host:guest stoichiometric halide association constants summarised in Table 2. Considering first the 1,3‐bis‐iodotriazole benzene incorporated axle and rotaxane i.e. **2** ⋅ **XB** and **3** ⋅ **XB**, as expected the acyclic axle **2** ⋅ **XB** exhibits the Hofmeister bias trend *K*_a_: I^−^>Br^−^>Cl^−^. In stark contrast, **3** ⋅ **XB** exhibits anti‐Hofmeister anion selectivity i.e. *K*_a_: Br^−^>Cl^−^>I^−^. Indeed, the determined *K*_a_ values of 3 330 M^−1^ and 6580 M^−1^ correspond to an 8‐ and 5‐fold mechanical bond enhancement effect for chloride and bromide respectively relative to non‐interlocked axle **2** ⋅ **XB**. Impressively, not only does the rotaxane exhibit a larger affinity for chloride over iodide, but the *K*_a_(I^−^) value of 1550 M^−1^ for **3** ⋅ **XB** is actually diminished relative to acyclic axle **2** ⋅ **XB** (*K*_a_(I^−^)=2190 M^−1^). These observations suggest that the smaller radii of Cl^−^ and Br^−^ permit endotopic anion binding, while the larger radius of iodide prohibits binding within the [2]rotaxane's cavity, therefore an exotopic coordination mode is adopted (Figure 5d).

**Table 2:** Halide anion association constants (*K*_a_) for the axle and [2]rotaxanes, conducted in D_2_O : acetone‐d_6_ mixtures.

	Anion Association Constant (*K*_a_/M^−1^)							
	D_2_O : acetone‐d_6_ (5 : 95, v/v)				D_2_O : acetone‐d_6_ (10 : 90, v/v)			
Anion^[a]^	**2** ⋅ **XB**	**3** ⋅ **XB**	**2** ⋅ **XB^(NO2)2^**	**3** ⋅ **XB^(NO2)2^**	**2** ⋅ **XB**	**3** ⋅ **XB**	**2** ⋅ **XB^(NO2)2^**	**3** ⋅ **XB^(NO2)2 [e]^**
Cl^−^	415 (11)	3 330 (121)	1 690 (15)	>10^5 [b]^	–^[d]^	274 (7)	206 (1)	3 800 (69)
Br^−^	1 320 (29)	6 580 (315)	5 900 (126)	>10^5 [b]^	236 (2)	461 (13)	955 (7)	5 760 (321)
I^−^	2 190 (103)	1 550 (25)	14 000 (1 200)	–^[c]^	760 (15)	551 (3)	3 050 (23)	2 660 (83)

[a] Halide added as the TBA salt. Errors (±) are in parentheses. [b] Too large to be accurately determined. [c] Complexity of chemical shift perturbations prevented reliable data fitting. [d] No binding. [e] Fitting details of titration data given in Supporting Information page S42.

This is not only consistent with enhanced chloride and bromide affinities of **3** ⋅ **XB** relative to **2** ⋅ **XB**, as anion binding is assisted by macrocycle HB‐anion interactions, but attenuated iodide affinity is likely a result of the mechanical bond topological inaccessibility of the XB binding site. Importantly, the ability to elicit anion binding selectivity which contrasts the anticipated trend on the basis of aqueous anion solvation presents a rare example of a synthetic receptor system exhibiting anti‐Hofmeister bias.

Analogous halide titration experiments in D_2_O : acetone‐d_6_ (5 : 95, v/v) were conducted on the dinitro functionalised axle, **2** ⋅ **XB^(NO2)2^** (monitoring proton signal b), and [2]rotaxane, **3** ⋅ **XB^(NO2)2^** (monitoring proton signal a, unless otherwise specified). As anticipated the **2** ⋅ **XB^(NO2)2^** axle anion affinities are consistently larger than those of **2** ⋅ **XB** and also exhibit Hofmeister bias, such that the *K*_a_ values are ordered I^−^>Br^−^>Cl^−^. Rotaxane **3** ⋅ **XB^(NO2)2^** exhibits increased chloride and bromide affinities relative to **2** ⋅ **XB^(NO2)2^** and **3** ⋅ **XB** such that *K*_a_ values could not be accurately determined i.e. >10^5^ M^−1^. In the case of iodide binding by **3** ⋅ **XB^(NO2)2^**, the situation is more complex as revealed by inspection of the generated isotherm (Figure S61). During the course of the titration the addition of up to ca. 2 equivalents of I^−^ induces a downfield or upfield perturbation in multiple proton signals associated with the binding cavity. The addition of more than 2 equivalents of I^−^ serves to reverse this respective direction of chemical shift perturbation. In light of these and other spectroscopic observations (Figure S56), it is clear that **3** ⋅ **XB^(NO2)2^**, as for **3** ⋅ **XB**, is binding iodide exotopically, while chloride and bromide are bound endotopically. The complexity of the iodide binding isotherm is attributed to a subtle combination of chemical shift perturbations arising from anion binding and macrocycle co‐conformation change which prohibited reliable fitting of the titration data.

Additional halide titration experiments were undertaken in an increased aqueous content solvent medium of D_2_O : acetone‐d_6_ (10 : 90, v/v) (Table 2). Predictably, the increased D_2_O content results in a universal decrease in halide anion *K*_a_ values for all receptors tested. However, pleasingly, inspection of the halide affinities for rotaxane **3** ⋅ **XB^(NO2)2^** demonstrate that despite the higher water percentage, an anti‐Hofmeister bias is still observed; *K*_a_(Cl^−^)=3 800 M^−1^, *K*_a_(Br^−^)=5 760 M^−1^ and *K*_a_(I^−^)=2 660 M^−1^. Whereas the acyclic axle **2** ⋅ **XB^(NO2)2^** displays a stronger iodide affinity *K*_a_(I^−^)=3 050 M^−1^ with Hofmeister halide selectivity. Despite the reduced iodide binding strength in comparison to axle component **2** ⋅ **XB**, rotaxane **3** ⋅ **XB** exhibits, albeit a very modest, Hofmeister governed trend in *K*_a_ values; *K*_a_(Cl^−^)=274 M^−1^, *K*_a_(Br^−^)=460 M^−1^ and *K*_a_(I^−^)=551 M^−1^, suggesting that anion hydration effects, now outcompete any advantages from a mechanical bond induced size exclusion effect.

To quantitatively evaluate the effect of the mechanical bond upon anion selectivity behaviour, “Mechanical Bond Enhancement” (MBE) factors, defined as MBE=(Log*K_a_* ^Rot^/Log*K_a_* ^Axle^) where *K_a_* ^Rot^ and *K_a_* ^Axle^ refer to the halide association constants of the [2]rotaxanes and axles respectively, were determined (Table 3).

**Table 3:** Mechanical Bond Enhancement (MBE) factors.

	Mechanical Bond Enhancement (MBE) Factor=(Log*K_a_* ^Rot^/Log*K_a_* ^Axle^)^[a]^	
	**3** ⋅ **XB/2** ⋅ **XB**	**3** ⋅ **XB^(NO2)2^/2** ⋅ **XB^(NO2)2^**
Anion	D_2_O : acetone‐d_6_ (5 : 95, v/v)	D_2_O : acetone‐d_6_ (10 : 90, v/v)
Cl^−^	1.35 (0.008)	1.55 (0.004)
Br^−^	1.22 (0.008)	1.26 (0.008)
I^−^	0.96 (0.006)	0.98 (0.004)

[a] Errors (±) are in parentheses.

**Figure 6:**
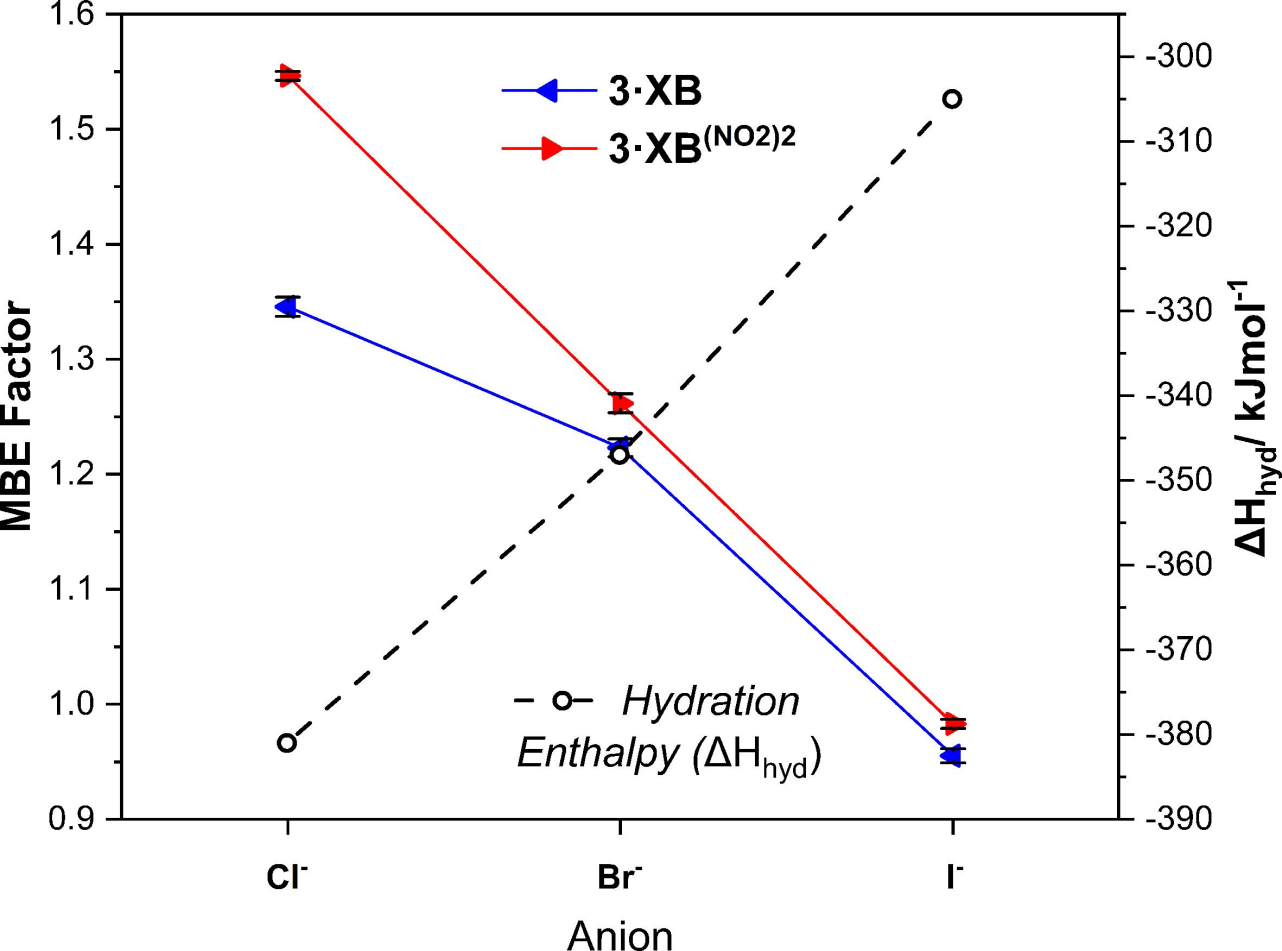
Plot showing MBE values for chloride, bromide and iodide for rotaxanes **3** ⋅ **XB** (blue) and **3** ⋅ **XB^(NO2)2^** (red) versus halide hydration enthalpies (black).

Importantly, this free‐energy analysis allows for a representative comparison of halide anion binding energetics and how they are modulated by the mechanical bond. Several important trends are elucidated from inspection of these MBE values. Firstly, as noted for the *K*_a_ values themselves the MBE values >1 observed for chloride and bromide indicate enhanced binding relative to the axle, while the MBE <1 for iodide show diminished halide affinity upon mechanical bond formation. Most importantly, it is also shown that the MBE values universally follow the trend Cl^−^>Br^−^>I^−^, such that the mechanical bond in essence inherently opposes the trend anticipated on the basis of the Hofmeister series. This is graphically visualised in Figure 6, wherein a plot of MBE value versus halide hydration enthalpies (Δ*H*_hyd_) for each halide show *directly* opposing trends. While the origin of this effect is almost certainly a halide size‐based discrimination, crucially this pronounced “mechanical bond enhancement” for more hydrophilic halides translates as a rare antagonist to selectivity profiles dictated by Hofmeister effects. Hence, in the wider context of anion recognition in aqueous media, employing neutral XB mechanically bonded host design to engineer anti‐Hofmeister bias anion selectivity is a promising potential general strategy to pursue in synthetic receptor design.

## Conclusion

In conclusion, we report the anion template synthesis of novel neutral XB [2]rotaxanes which exhibit anti‐Hofmeister halide anion recognition in water containing solvent media. Maximising the strength of XB‐chloride anion interactions through axle component containing perfluoroaryl‐functionalised bis‐iodotriazole XB donors including a remarkably potent novel 4,6‐dinitro‐1,3‐bis‐iodotriazole motif, enabled the unprecedented construction of a neutral XB mechanically interlocked molecule (MIM) via a discrete anion template directed strategy. Extensive ^1^H NMR halide anion recognition studies of the [2]rotaxane series in aqueous‐organic solvent mixtures revealed pronounced anti‐Hofmeister bias, in stark contrast to the non‐interlocked acyclic axle components. Free‐energy analysis of this “mechanical bond effect” on halide binding affinities demonstrate preferential stabilisation of hydrophilic chloride over hydrophobic iodide, thereby directly opposing hydration‐driven binding selectivity profiles. Importantly, these results serve to highlight the exploitation of highly potent neutral XB donor interlocked topologies in synthetic receptor design is not only capable of dramatically raising the bar in terms of anion affinity in aqueous media, but crucially provides a rare and potentially powerful strategy to directly oppose Hofmeister selectivity bias.

## Conflict of interest

The authors declare no conflict of interest.

1

## Supporting information

As a service to our authors and readers, this journal provides supporting information supplied by the authors. Such materials are peer reviewed and may be re‐organized for online delivery, but are not copy‐edited or typeset. Technical support issues arising from supporting information (other than missing files) should be addressed to the authors.

Supporting Information

## Data Availability

The data that support the findings of this study are available in the supplementary material of this article.
