# Supplementary material for: Anti‐Hofmeister Anion Selectivity via a Mechanical Bond Effect in Neutral Halogen‐Bonding [2]Rotaxanes
Source: Angew Chem Int Ed Engl. 2022 Nov 17;61(50):e202214523. doi: 10.1002/anie.202214523 (PMC10100147; doi:10.1002/anie.202214523)
Supplement: Supplementary file 1 — Supporting Information [file ANIE-61-0-s001.pdf]

Angewandte Chemie
Eine Zeitschrift der Gesellschaft Deutscher Chemiker

Supporting Information

# Anti-Hofmeister Anion Selectivity via a Mechanical Bond Effect in Neutral Halogen-Bonding [2]Rotaxanes

*A. Docker, Y. C. Tse, H. M. Tay, A. J. Taylor, Z. Zhang, P. D. Beer**

**Supporting information**

**Contents**

1. Materials and Methods ........ 2
2. Synthesis and Characterisation ........ 3
3. $^{1}$H NMR Anion Recognition Studies ........ 38
$^{1}$H NMR anion titration protocol ........ 38
Discussion regarding chemical shift perturbations observed during anion titration experiments ........ 39
Summarised anion binding isotherms and stacked $^{1}$H NMR spectra ........ 44
4. 2D $^{1}$H-$^{1}$H ROESY NMR Spectra ........ 80
5. Single Crystal Diffraction Data ........ 82
6. References ........ 84

## 1. Materials and Methods

All solvents and reagents were purchased from commercial suppliers and used as received unless otherwise stated. Dry solvents were obtained by purging with nitrogen and then passing through an MBraun MPSP-800 column. $H_2O$ was de-ionized and micro filtered using a Milli-Q ® Millipore machine. Column chromatography was carried out on Merck® silica gel 60 under a positive pressure of nitrogen. Routine NMR spectra were recorded on either a Varian Mercury 300, a Bruker AVIII 400 or a Bruker AVIII 500 spectrometer with $^1$H NMR titrations recorded on a Bruker AVIII 500 spectrometer. TBA salts were stored in a vacuum desiccator containing phosphorus pentoxide prior to use. Where mixtures of solvents were used, ratios are reported by volume. Chemical shifts are quoted in parts per million relative to the residual solvent peak. Mass spectra were recorded on a Bruker µTOF spectrometer. Triethylamine was distilled from and stored over potassium hydroxide. Tris[(1-benzyl-1H-1,2,3-triazol-4-yl)methyl]amine (TBTA).

## 2. Synthesis and Characterisation

### General Procedure 1 (GP1)

$[Cu(MeCN)_4]PF_6$ (0.2 eq.) and TBTA (0.2 eq.) were dissolved in dry, degassed $CH_2Cl_2$ (ca. 3 mL) and stirred for 30 minutes. The respective bis-alkyne was added (1 eq.) in dry, degassed $CH_2Cl_2$ (2 mL) before the addition of perfluorophenyl azide (2.2 eq.). The resulting mixture was stirred at room temperature overnight under an atmosphere of $N_2$. The crude was diluted with $CH_2Cl_2$ and washed with an EDTA/$NH_4OH_{(aq)}$ solution followed by brine before drying the organic layer over $MgSO_4$ and concentrated under reduced pressure. Products were purified using silica gel flash column chromatography.

**3**

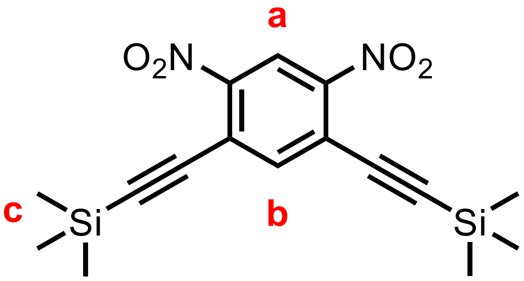


**2** (1.00 g, 3.08 mmol), $Pd(PPh_3)_4$ (177 mg, 1.53 mmol) and CuI (58 mg, 0.305 mmol) were suspended in dry degassed THF (20 ml), to which was added DIPEA (5.34 ml, 30.7 mmol) and TMS acetylene (4.34 ml, 30.6 mmol) and left to stir for 5 hours at room temperature. After which time the mixture was filtered through celite, the filtrate concentrated *in vacuo* and the crude residue dissolved in $CH_2Cl_2$, the organic layer was washed with a 0.1 M $NH_4OH/EDTA_{(aq)}$ solution, followed by brine. The organic layer was dried over $MgSO_4$ and concentrated to dryness, after which the crude was subjected to silica gel column chromatography ($CH_2Cl_2$:hexane, 3:7, v/v) to afford **3** as a yellow solid (533 mg, 1.48 mmol, 48%).

**$^1$H NMR** (500 MHz, $CDCl_3$) δ 8.75 (s, 1$H_a$), 7.91 (s, 1$H_b$), 0.30 (s, 18$H_c$).

**$^{13}$C NMR** (126 MHz, $CDCl_3$) δ 77.95, 71.84, 53.07, 51.83, 41.25, 27.36, -70.38.

**HRMS** (ESI+ve) m/z: 361.1033 ($[M+H]^+$, $C_{16}H_{21}O_4N_2Si_2$ requires 361.1034)

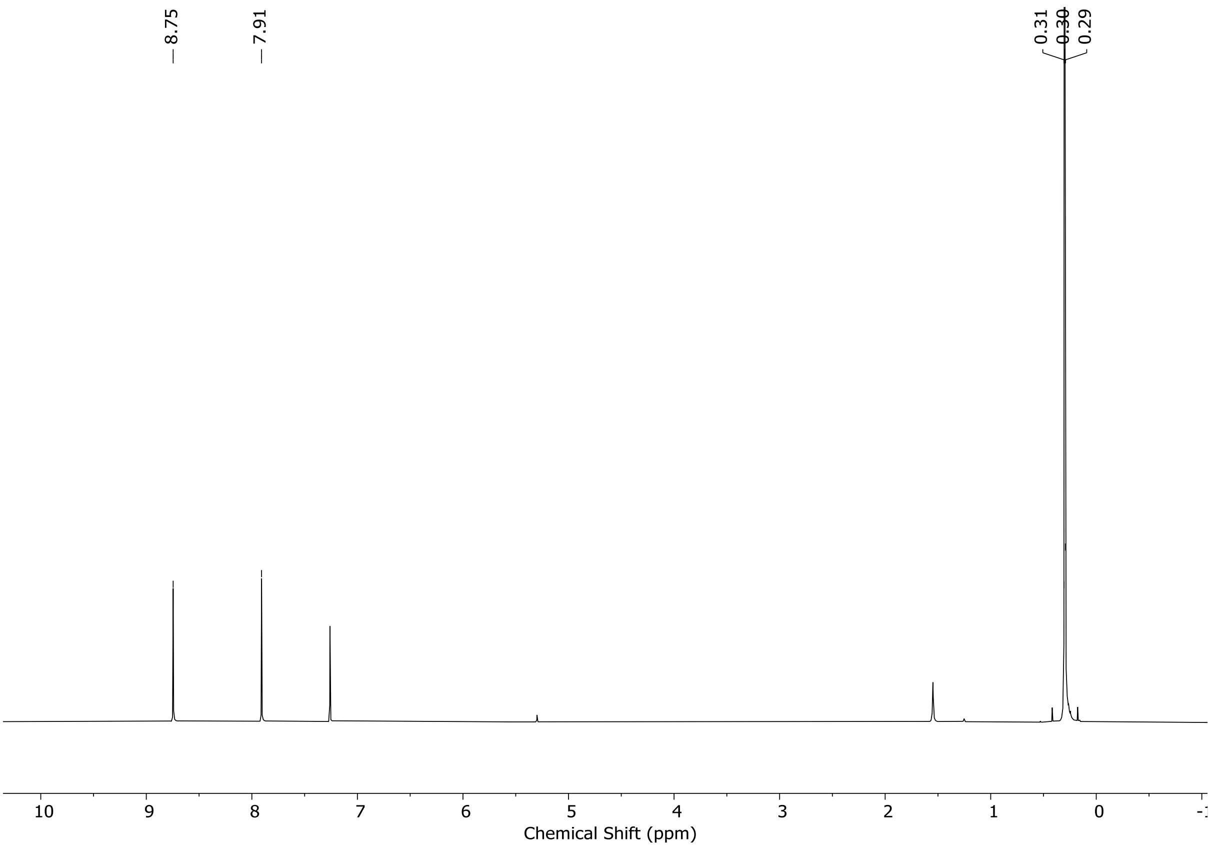


Figure S1. $^{1}H$ NMR spectrum of **3** ($CDCl_3$, 298 K, 500 MHz).

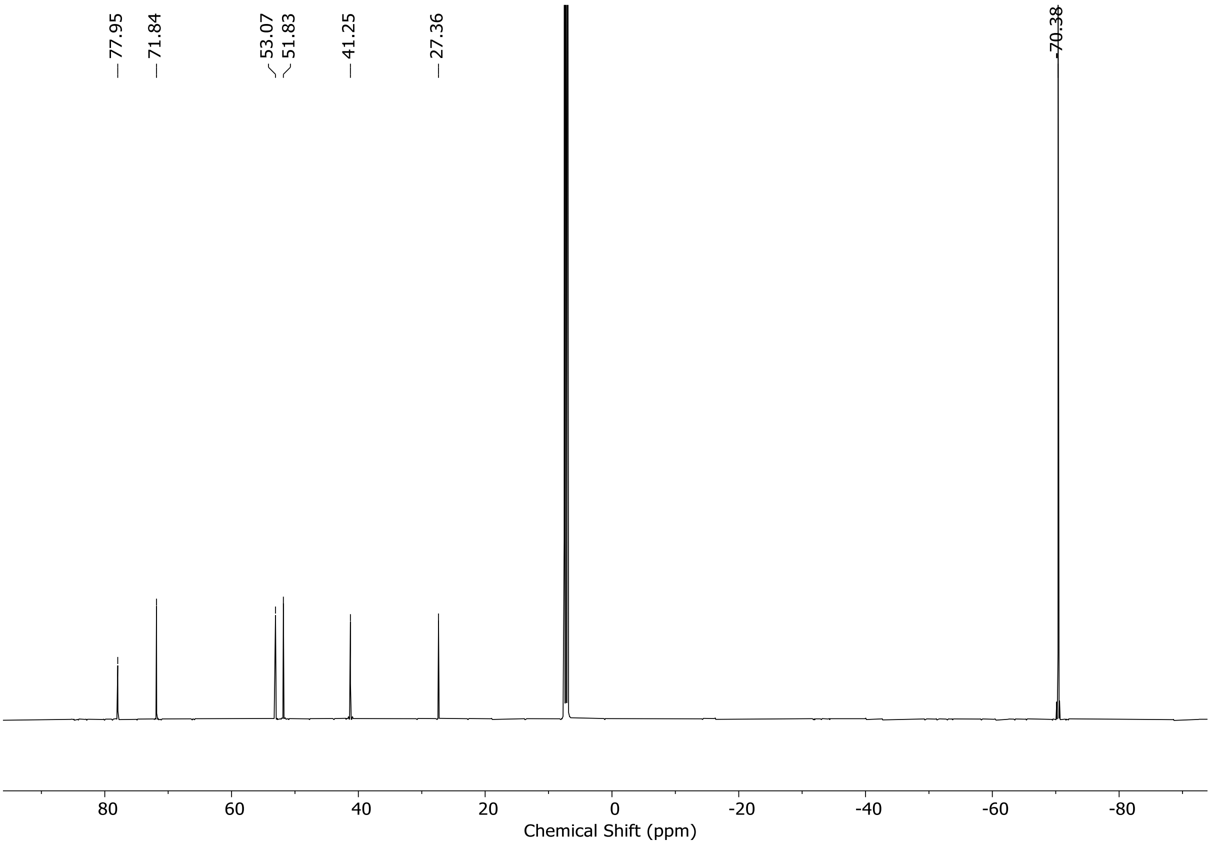


Figure S2. $^{13}C$ NMR spectrum of **3** ($CDCl_3$, 298 K, 126 MHz).

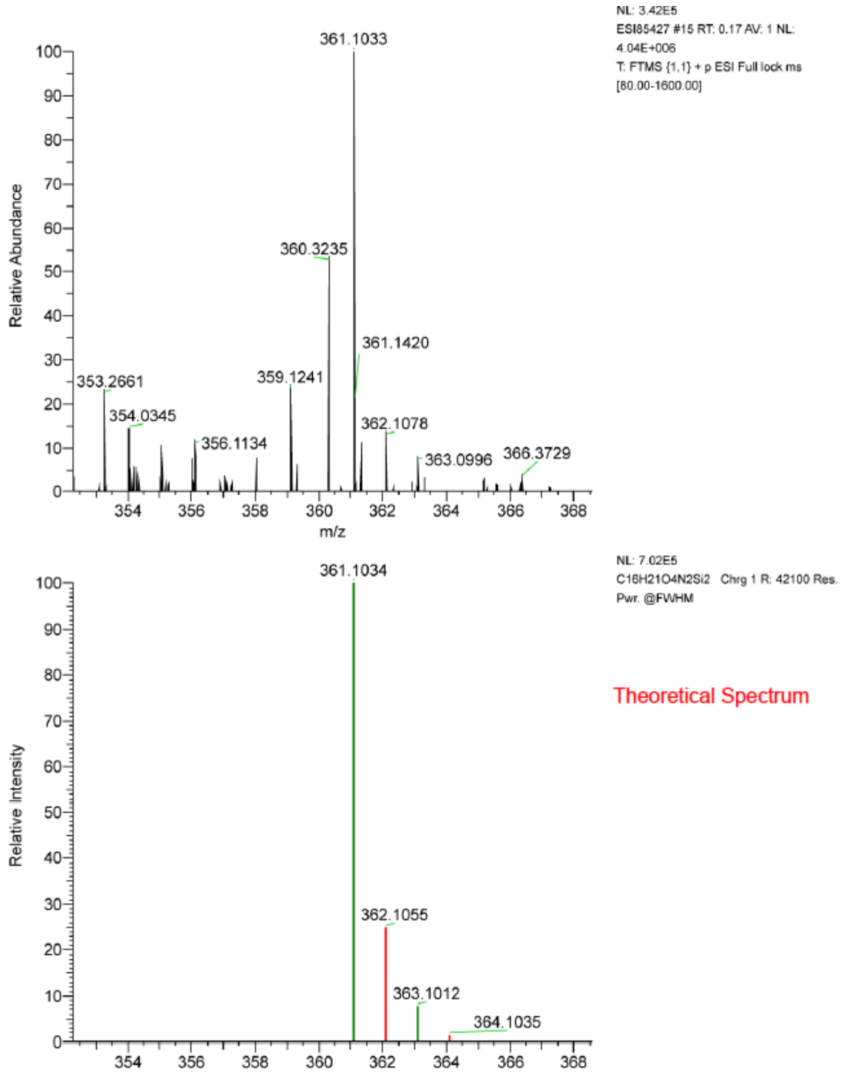


Figure S3. HRESI-MS of **3**.

**4**

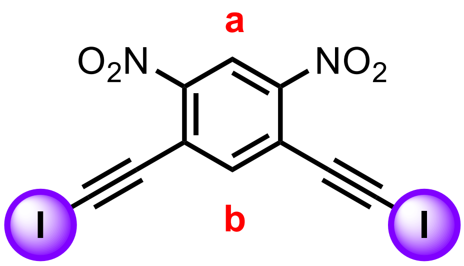


**3** (520 mg, 1.44 mmol) was dissolved in anhydrous DMF (20 ml), to which was added $AgNO_3$ (48 mg, 0.213 mmol) and cooled to 0 °C. At which point NIS (735 mg, 4.33 mmol) was added portion wise and left to stir at 0 °C for 10 minutes, after which the reaction was allowed to stir at room temperature for 2 hours, excluded from light. The reaction mixture was diluted with $H_2O$ (250 ml) and extracted with $Et_2O$ (ca. 500 ml), the collected organic phases were washed with $H_2O$ and dried over $MgSO_4$ and concentrated *in vacuo* to afford **4** as a yellow solid (655 mg, 1.40 mmol, Quant.).

**$^1$H NMR** (500 MHz, $CDCl_3$) δ 8.81 (s, 1$H_a$), 7.92 (s, 1$H_b$).

**$^{13}$C NMR** (126 MHz, $CDCl_3$) δ 148.28, 143.60, 123.19, 122.01, 87.53, 26.15.

**HRMS** (MSS-ve) m/z: 467.8091 ($[M]^-$, $C_{10}H_2O_4N_2I_2$ requires 467.8109).

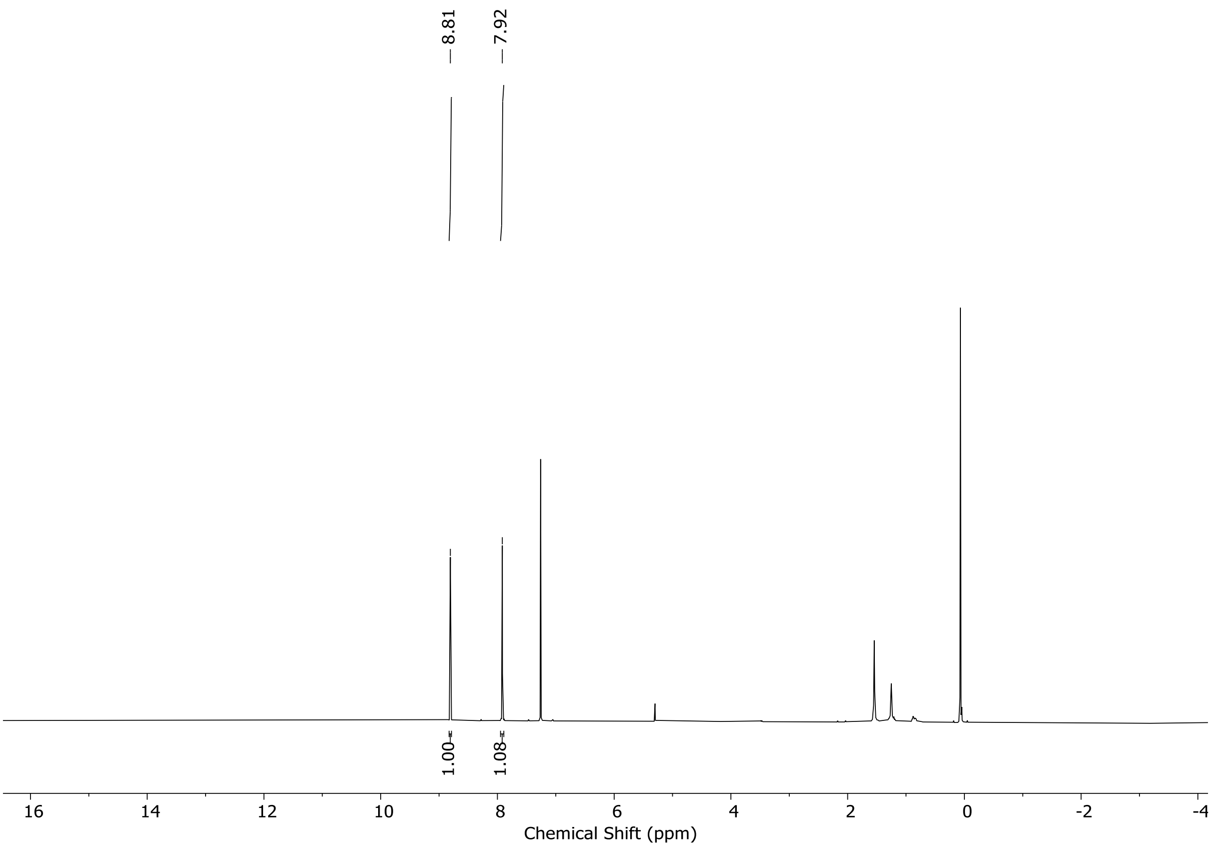


Figure S4. $^{1}H$ NMR spectrum of **4** ($CDCl_3$, 298 K, 500 MHz).

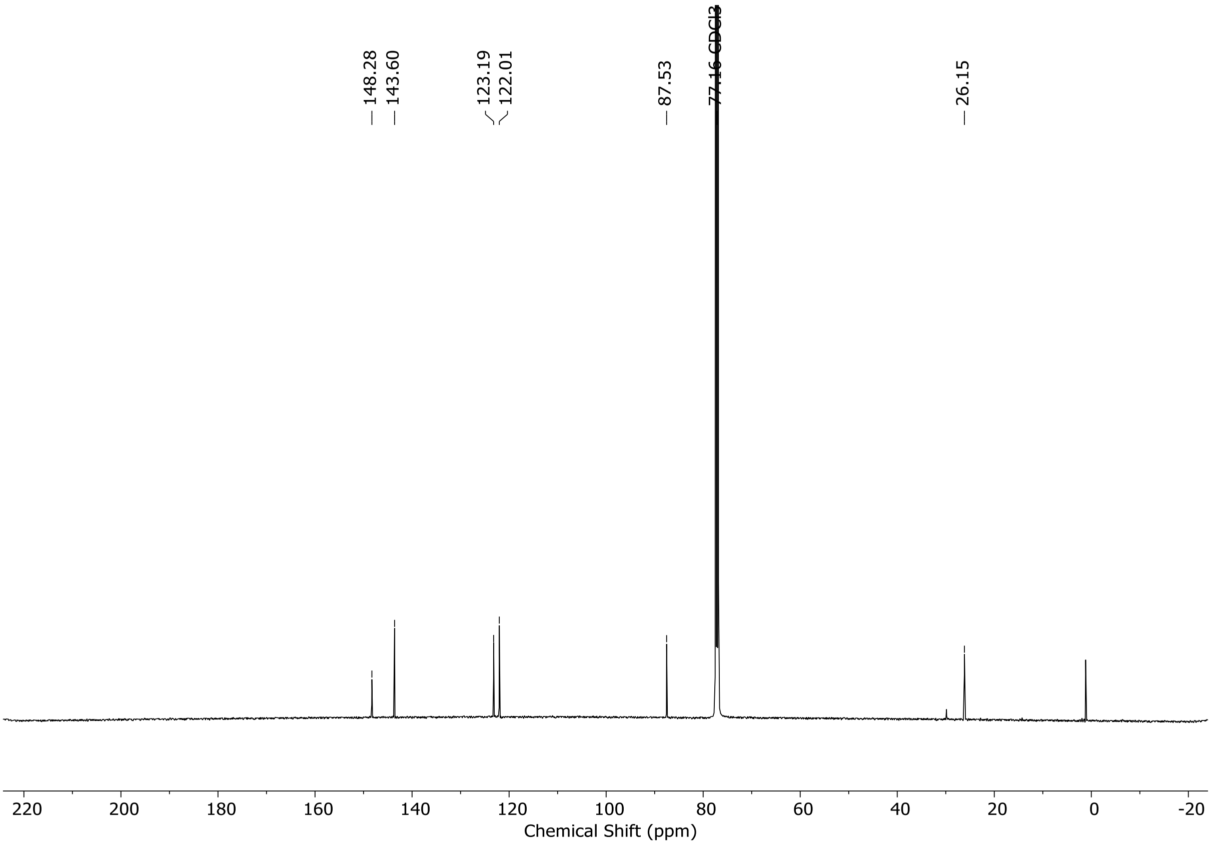


Figure S5.$^{13}C$ NMR spectrum of **4** ($CDCl_3$, 298 K, 126 MHz).

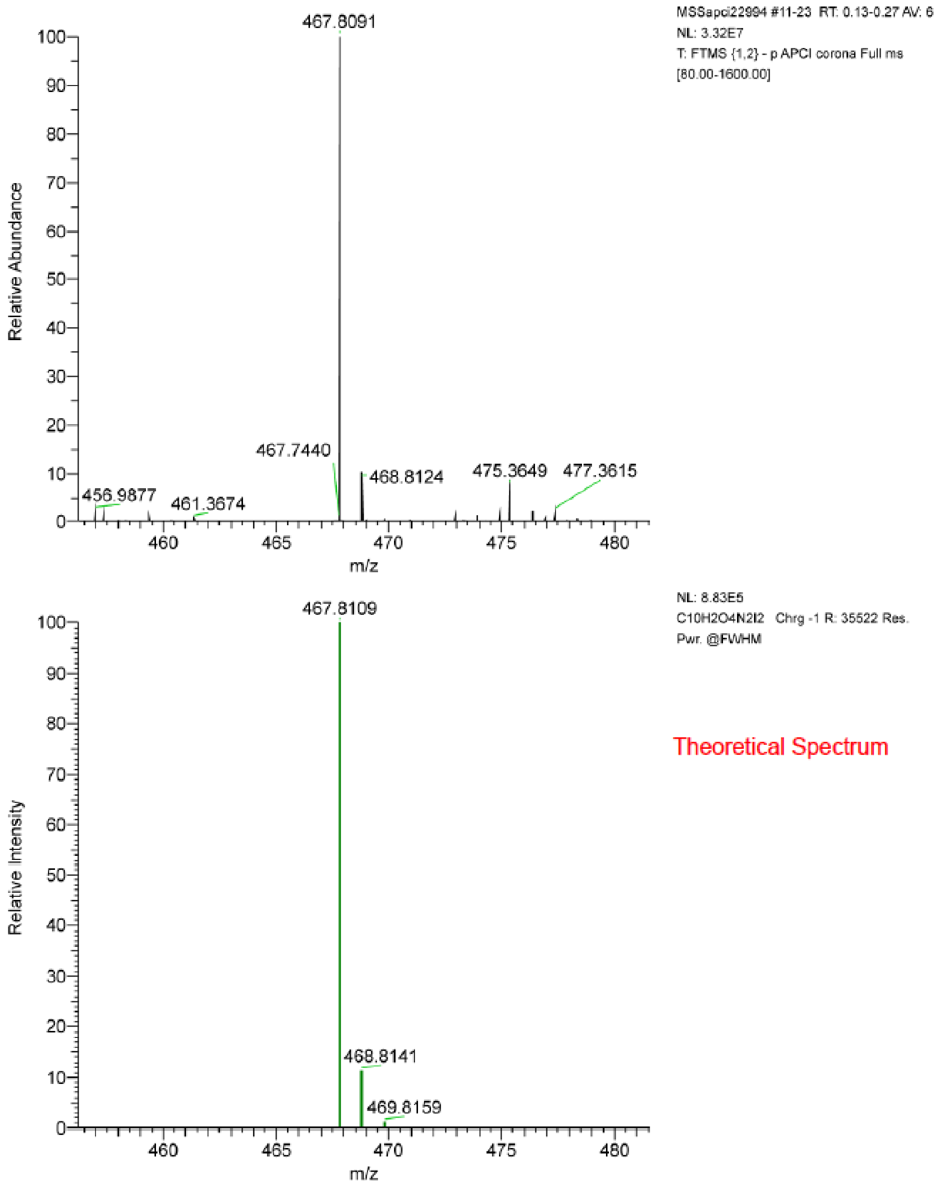


Figure S6. HRESI-MS of **4**.

**1·XB**

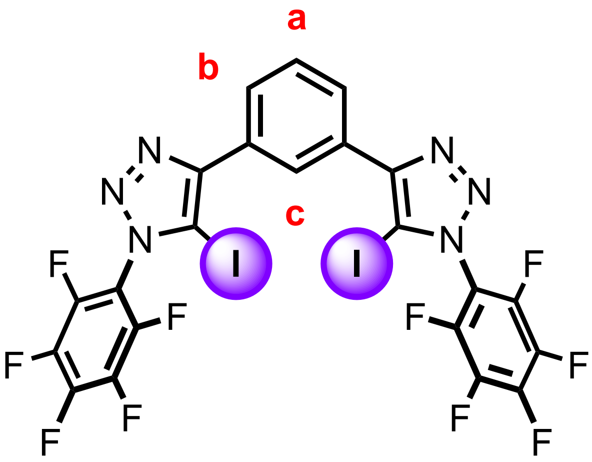


Synthesised *via* GP1.

Isolated as white solid (70%).

**$^{1}$H NMR** (500 MHz, $CDCl_3$) δ 8.75 (t, *J* = 1.7 Hz, 1$H_c$), 8.16 (dd, *J* = 7.8, 1.8 Hz, 2$H_b$), 7.69 (t, *J* = 7.8 Hz, 1$H_a$).

**$^{19}$F NMR** (470 MHz, $CDCl_3$) δ -142.18 (dt, *J* = 19.6, 3.8 Hz), -145.56 – -149.33 (m), -156.79 – -162.40 (m).

**$^{13}$C NMR** (126 MHz, $CDCl_3$) δ 150.20, 143.82 (dm, *J* = 261 Hz), 143.62 (dm, *J* = 256 Hz), 138.13 (dm, *J* = 255 Hz), 129.91, 129.51, 128.30, 126.27, 112.49, 80.56.

**HRMS** (ESI+ve) m/z: 796.8512 ($[M+H]^+$, $C_{22}H_5F_{10}N_6I_2$ requires 796.8505).

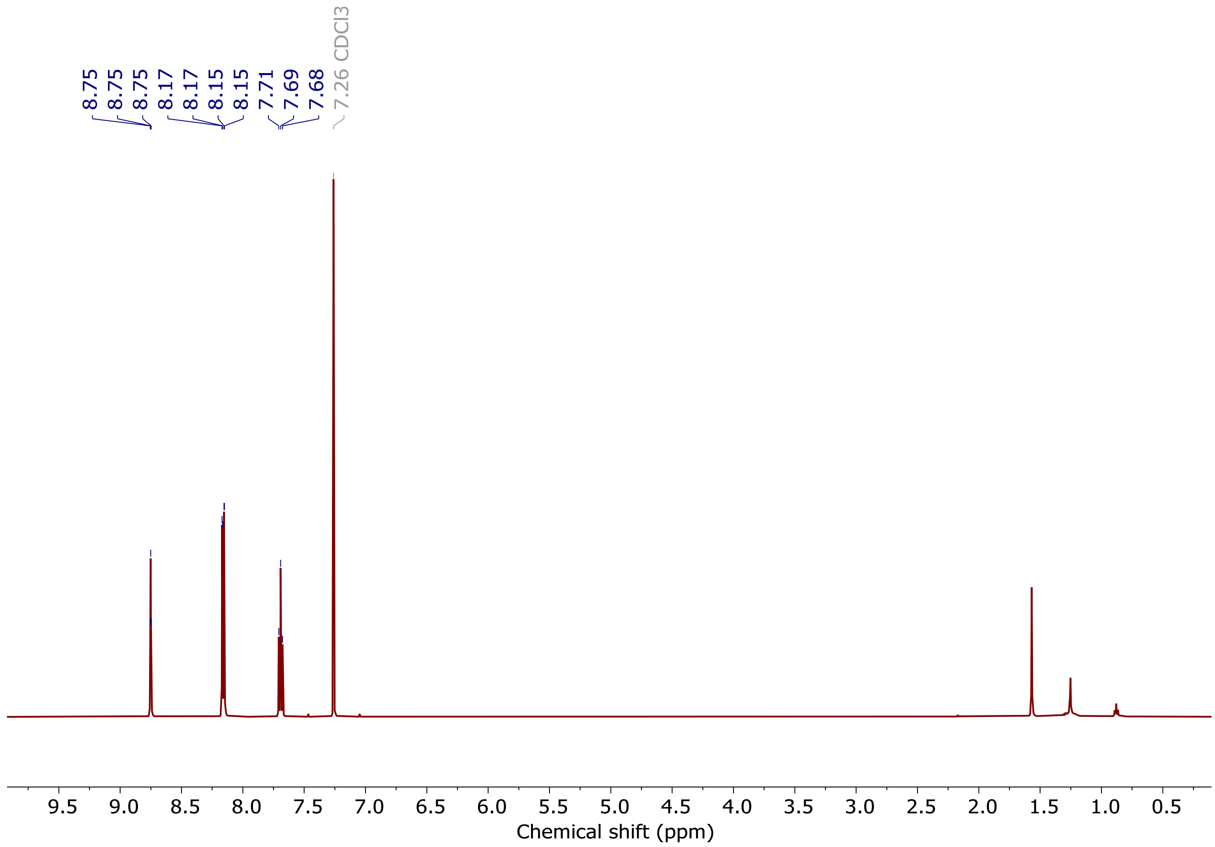


Figure S7. $^{1}H$ NMR spectrum of **1·XB** ($CDCl_3$, 298 K, 500 MHz).

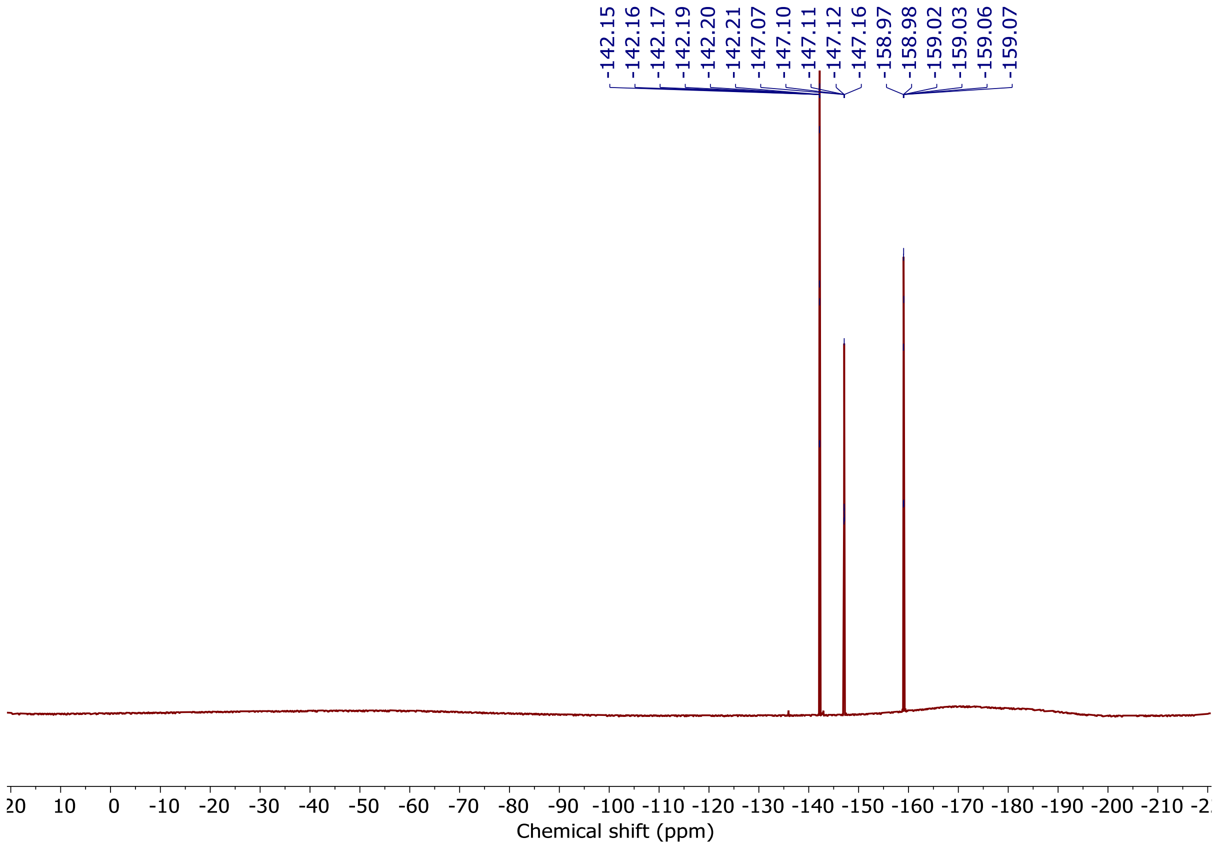


Figure S8. $^{19}F$ NMR spectrum of **1·XB** ($CDCl_3$, 298 K, 470 MHz).

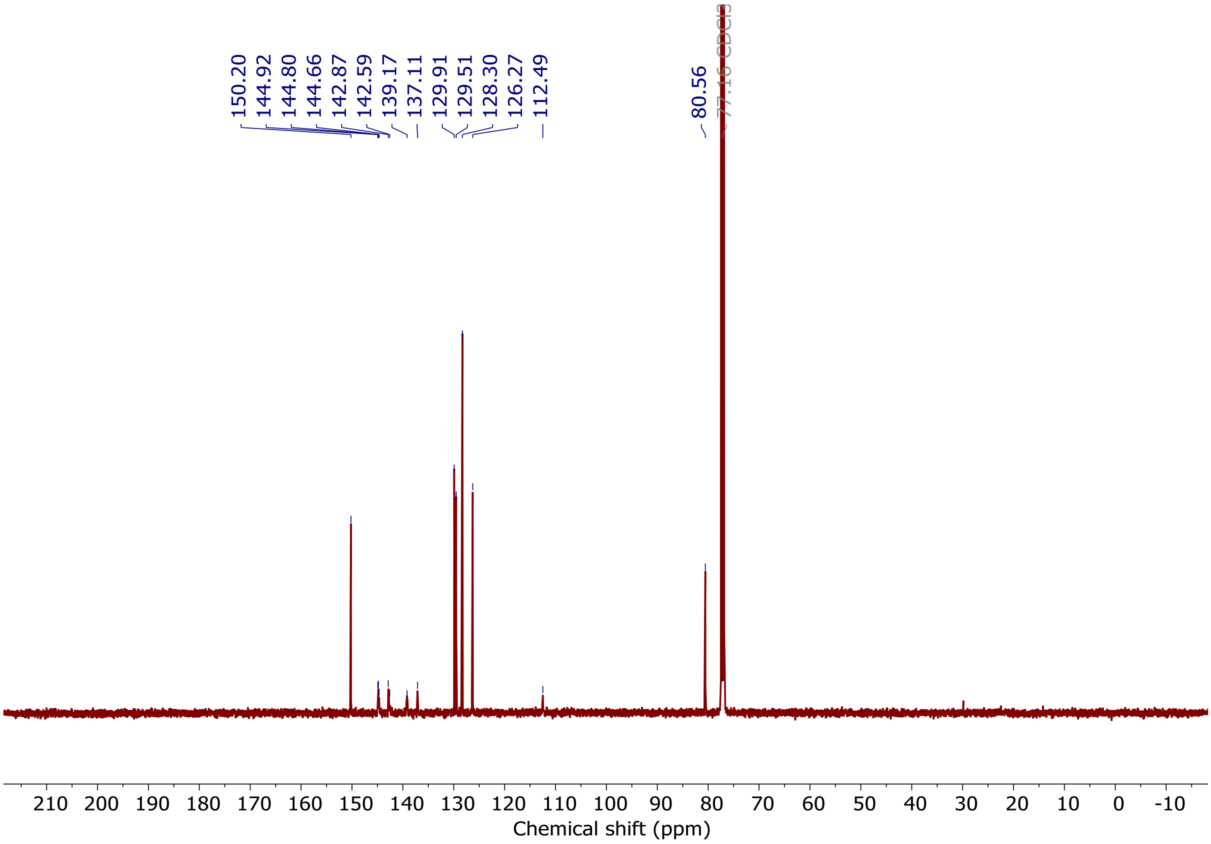


Figure S9. $^{13}$C NMR spectrum of **1·XB** ($CDCl_3$, 298 K, 126 MHz).

**1·HB**

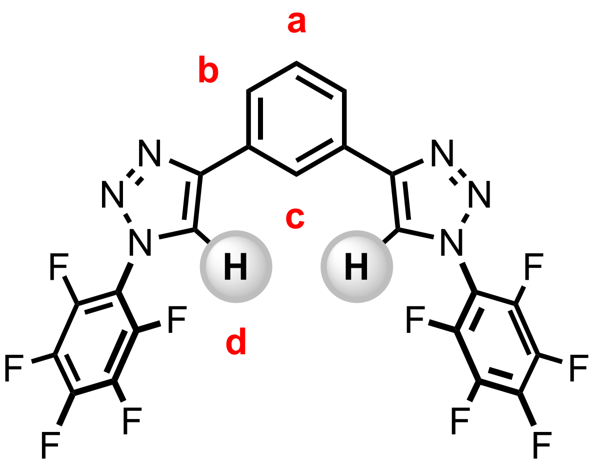


Synthesised *via* GP1.

Isolated as white solid (36%).

**$^{1}$H NMR** (500 MHz, DMSO) δ 9.26 (s, 2$H_d$), 8.58 (t, *J* = 1.7 Hz, 1$H_c$), 8.00 (dd, *J* = 7.8, 1.7 Hz, 2$H_b$), 7.67 (t, *J* = 7.8 Hz, 1$H_a$).

**$^{19}$F NMR** (470 MHz, DMSO) δ -146.70 – -147.48 (m), -151.18 (t, *J* = 22.9 Hz), -160.82 (td, *J* = 21.4, 3.0 Hz).

**$^{13}$C NMR** (126 MHz, DMSO) δ 146.67, 143.34 –140.97 (Coincident $^{13}$C signals), 137.68 (d, *J* = 251.9 Hz), 130.19, 130.05, 125.83, 124.72, 122.55, 112.47 (d, *J* = 13.6 Hz).

**HRMS** (ESI+ve) m/z: 545.0578 ($[M+H]^+$, $C_{22}H_7F_{10}N_6$ requires 545.0573).

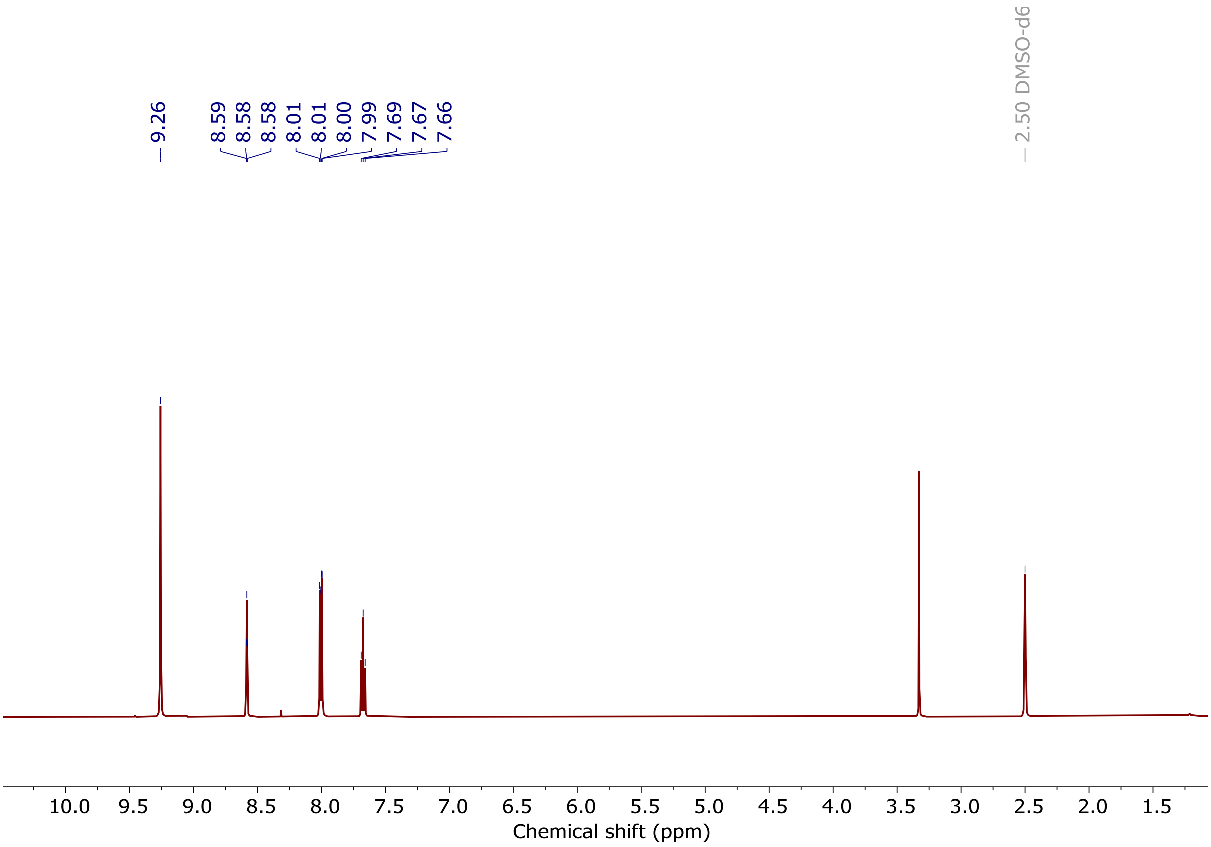


Figure S10. $^{1}H$ NMR spectrum of **1·HB** (DMSO, 298 K, 500 MHz).

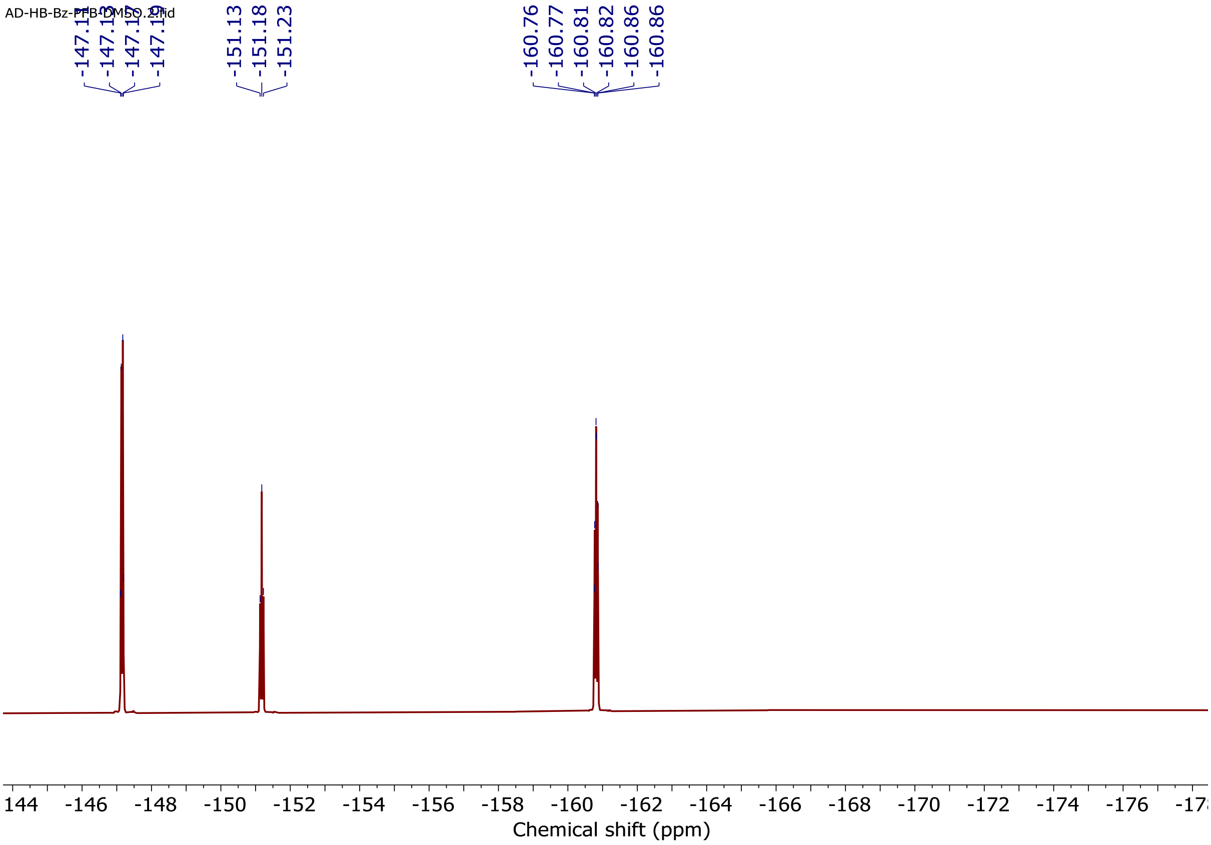


Figure S11. $^{19}F$ NMR spectrum of **1·HB** (DMSO, 298 K, 470 MHz).

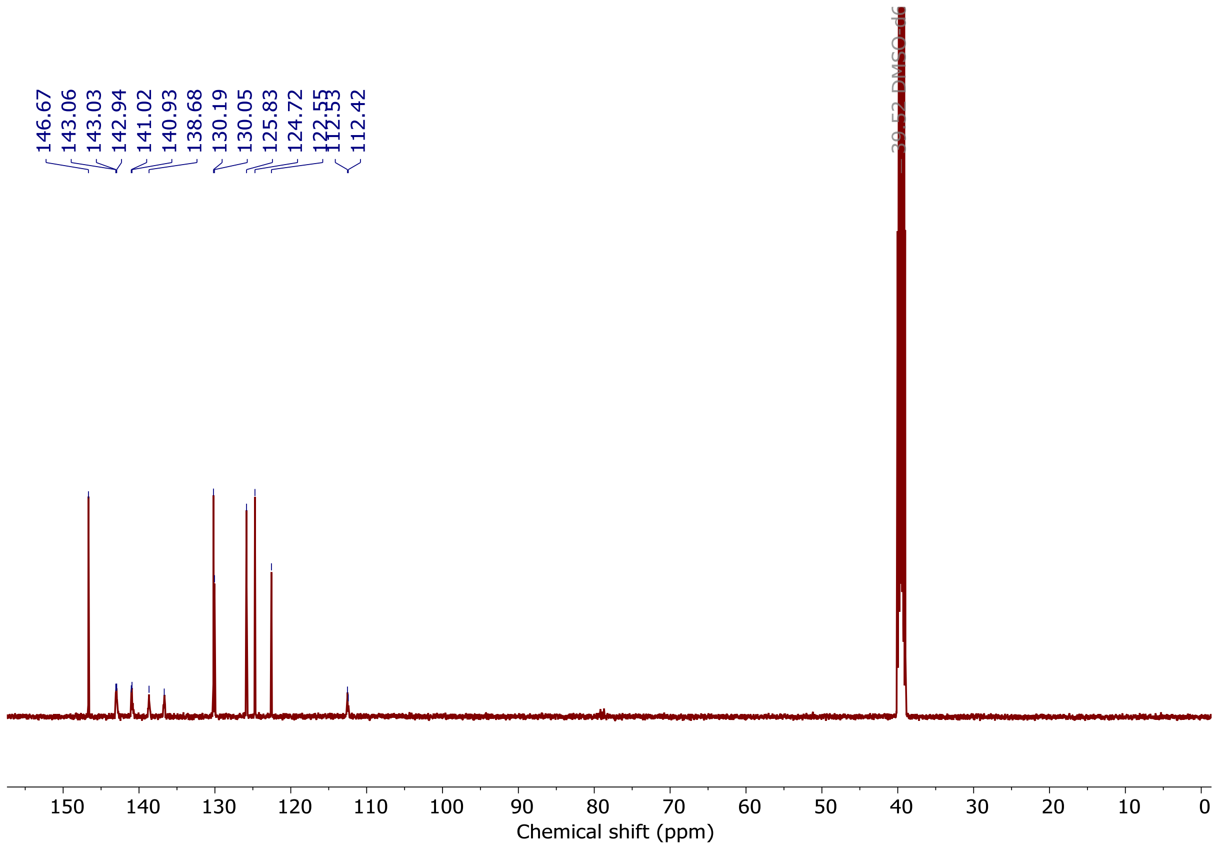


Figure S12. $^{13}C$ NMR spectrum of **1·HB** (DMSO, 298 K, 126 MHz).

**1·XB^(NO2)2**

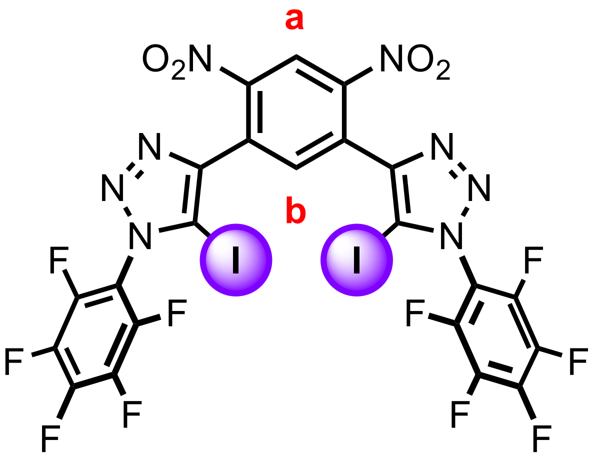


Synthesised *via* GP1.

Isolated as yellow solid (Quant.).

**$^{1}$H NMR** (500 MHz, $CDCl_3$) δ 8.91 (d, *J* = 4.5 Hz, $1H_a$), 8.18 (d, *J* = 4.5 Hz, $1H_b$).

**$^{13}$C NMR** (126 MHz, $CDCl_3$) δ 148.28, 147.06, 143.95 (dm, *J* = 263 Hz), 143.95 (dm, *J* = 263 Hz), 143.68 (dm, *J* = 258 Hz), 138.22 (dm, *J* = 257 Hz) 137.44, 128.92, 122.68, 111.85 (t, *J* = 13.9 Hz), 84.84.

**HRMS** (ESI+ve) m/z: 886.8192 ($[M+H]^+$, $C_{22}H_3O_4N_8F_{10}I_2$ requires 886.8201).

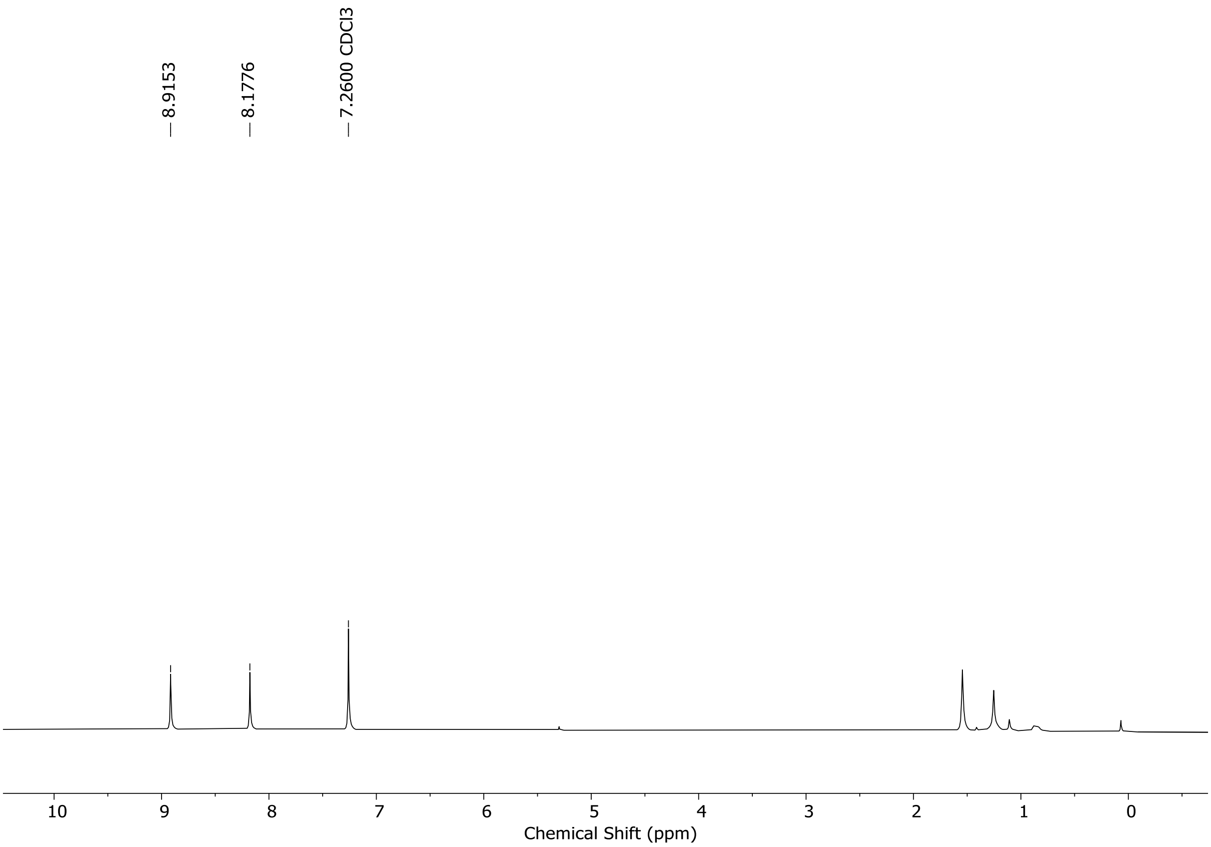


Figure S13. $^{1}H$ NMR spectrum of **1·XB$^{(NO2)2}$** ($CDCl_3$, 298 K, 500 MHz).

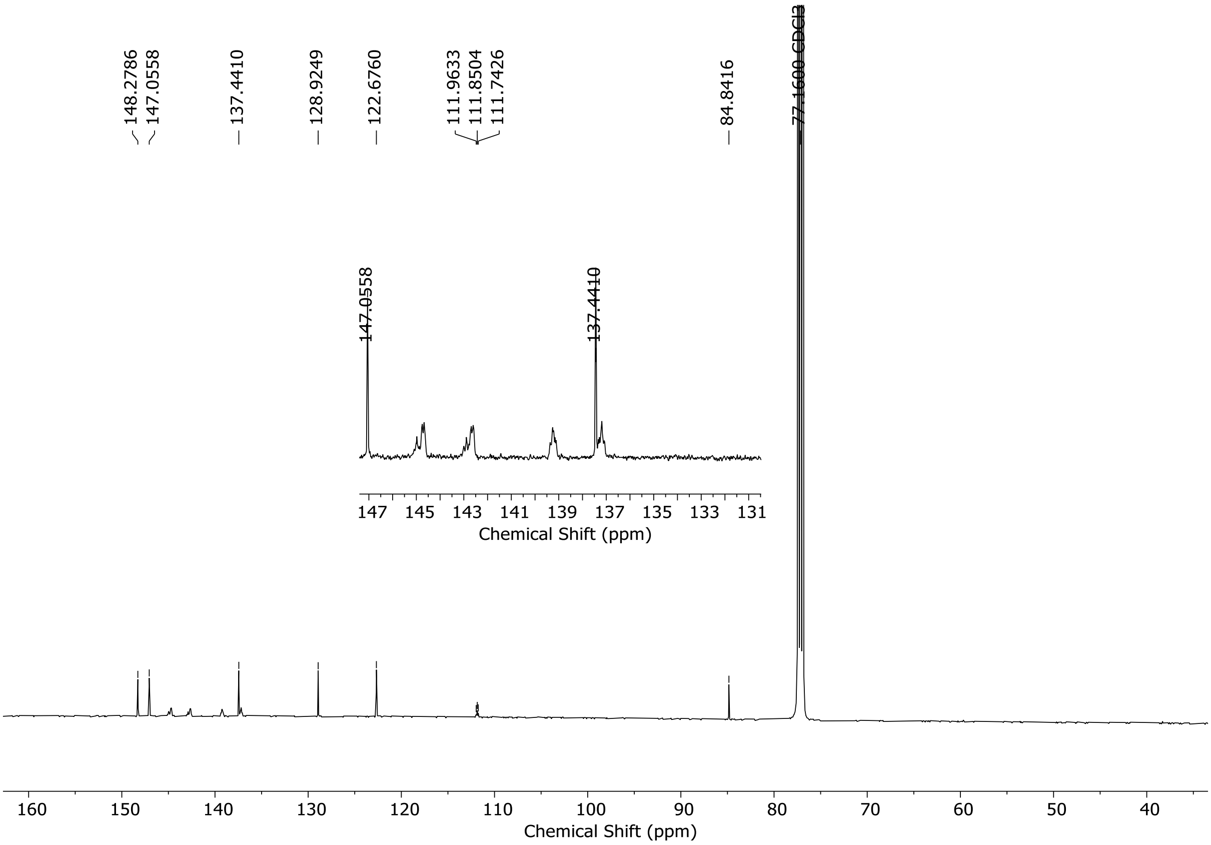


Figure S14. $^{13}C$ NMR spectrum of **1·XB$^{(NO2)2}$** ($CDCl_3$, 298 K, 126 MHz).

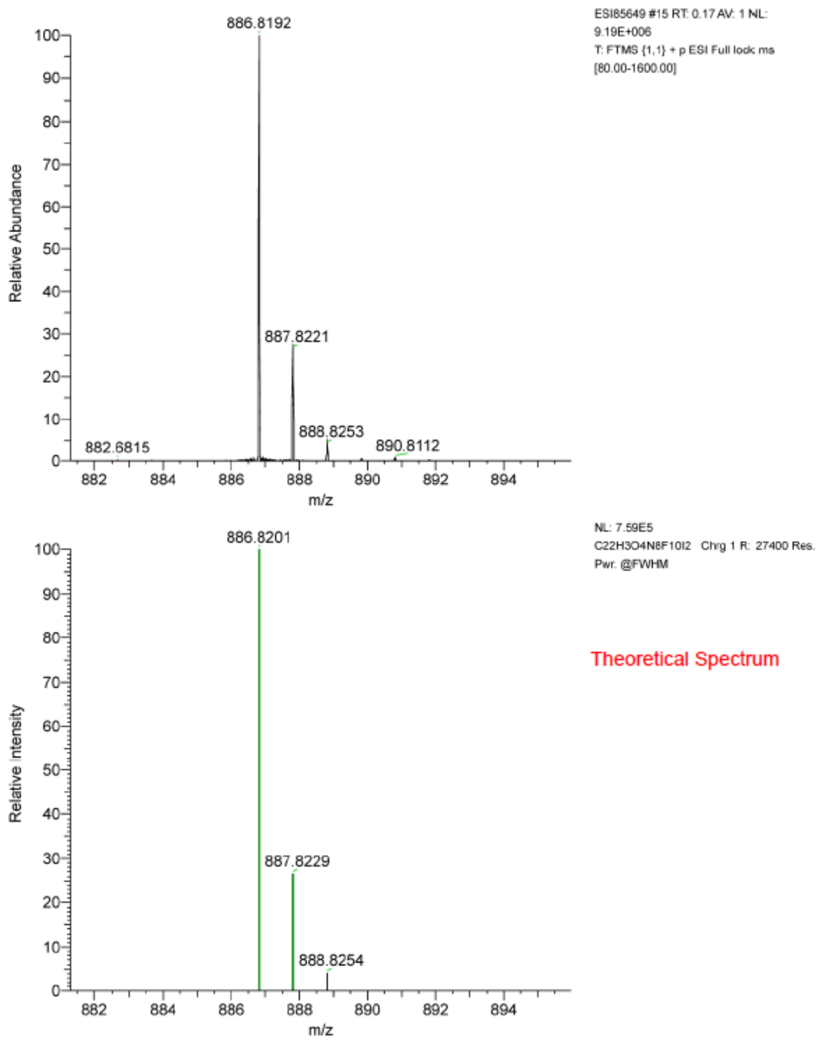


Figure S15. HRESI-MS of **1·XB$^{(NO2)2}$**.

**9**

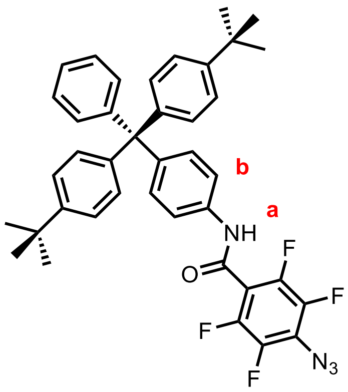


**7** (350 mg, 0.781 mmol) and **8** (202 mg, 0.859 mmol) were dissolved in anhydrous $CH_2Cl_2$ (10 ml), to which was added DCC (161 mg, 0.781 mmol) and DMAP (5 mg, cat.), the mixture was left to stir at room temperature for 2 hours. After which time the reaction mixture was filtered and the solid carefully washed with $CH_2Cl_2$ (10 ml), the collected filtrate was concentrated to dryness *in vacuo* and purified by silica gel column chromatography (hexane:$CH_2Cl_2$) to afford **9** as a white solid (405 mg, 0.609 mmol, 78 %).

**$^1$H NMR** (500 MHz, $CDCl_3$) δ 7.63 (s, 1$H_a$), 7.50 – 7.43 (m, 2$H_b$), 7.32 – 7.16 (m, 11H), 7.15 – 7.08 (m, 4H), 1.31 (s, 18H).

**$^{19}$F NMR** (470 MHz, $CDCl_3$) δ -140.26 – -140.72 (m), -149.79 – -150.34 (m).

**$^{13}$C NMR** (126 MHz, $CDCl_3$) δ 155.59, 148.74, 147.05, 145.18 (dm, $J$ = 256 Hz), 144.94, 143.62, 140.68 (dm, $J$ = 252 Hz), 134.51, 132.10, 131.23, 130.78, 127.54, 126.00, 124.46, 122.57 (m) 119.21, 111.55 (t, $J$ = 18.2 Hz), 63.97, 34.46, 31.50.

**HRMS** (ESI+ve) m/z: 665.2893 ($[M+H]^+$, $C_{40}H_{37}ON_4F_4$ requires 665.2898)

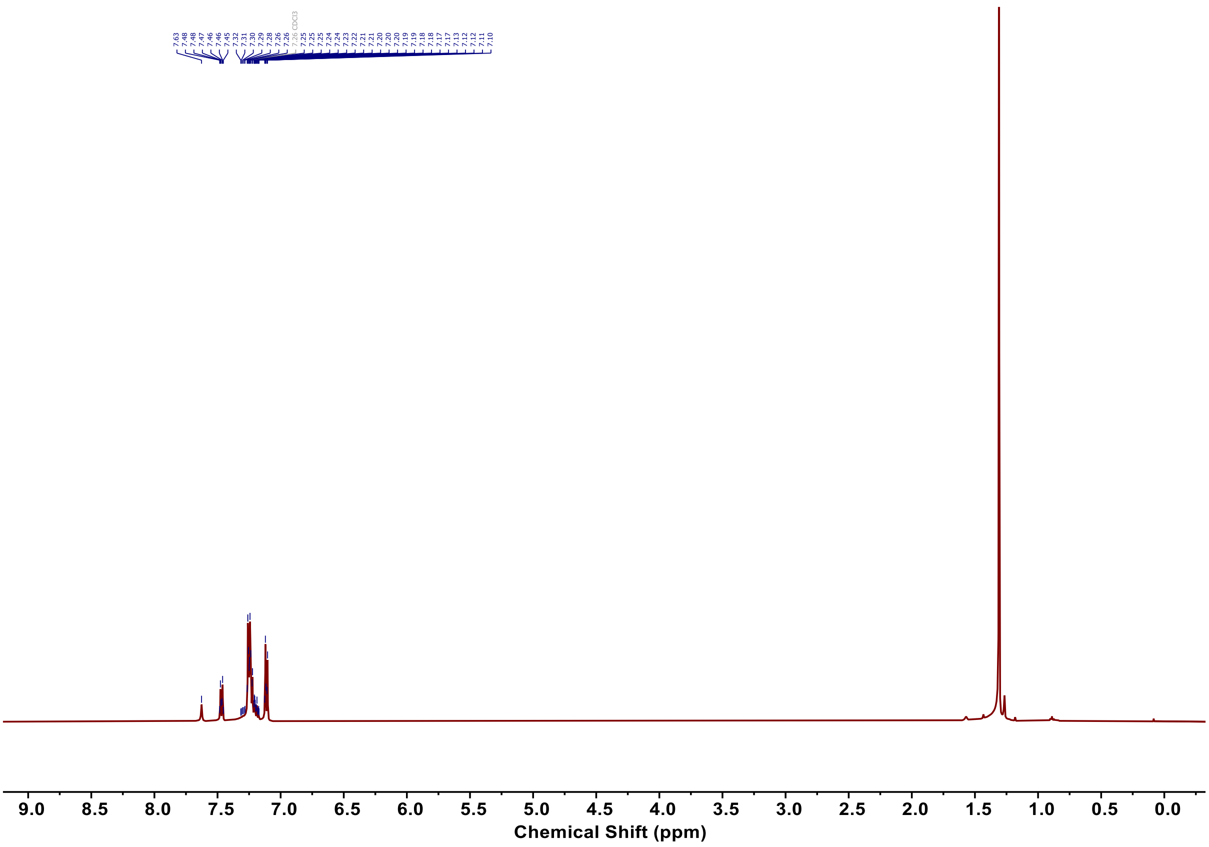


Figure S16. $^{1}$H NMR spectrum of **9** ($CDCl_3$, 298 K, 500 MHz).

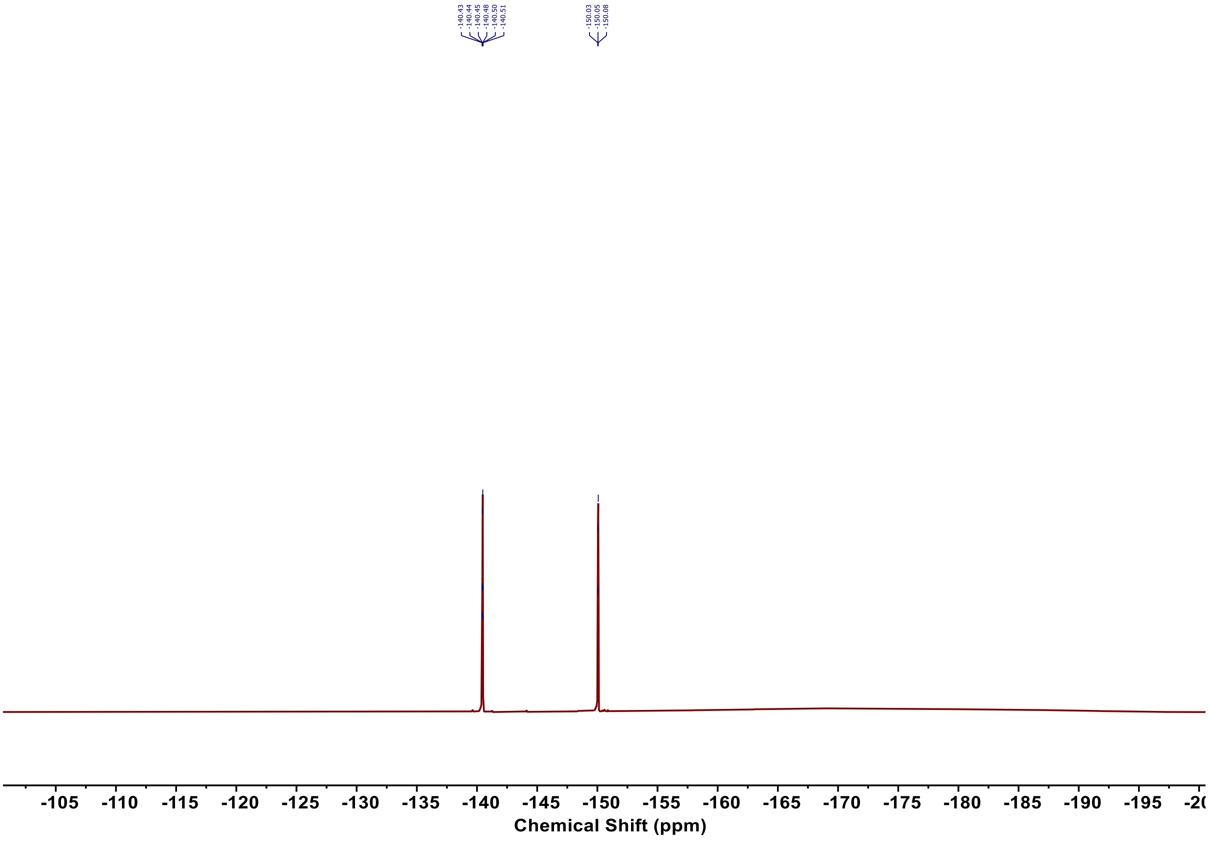


Figure S17. $^{19}$F NMR spectrum of **9** ($CDCl_3$, 298 K, 470 MHz).

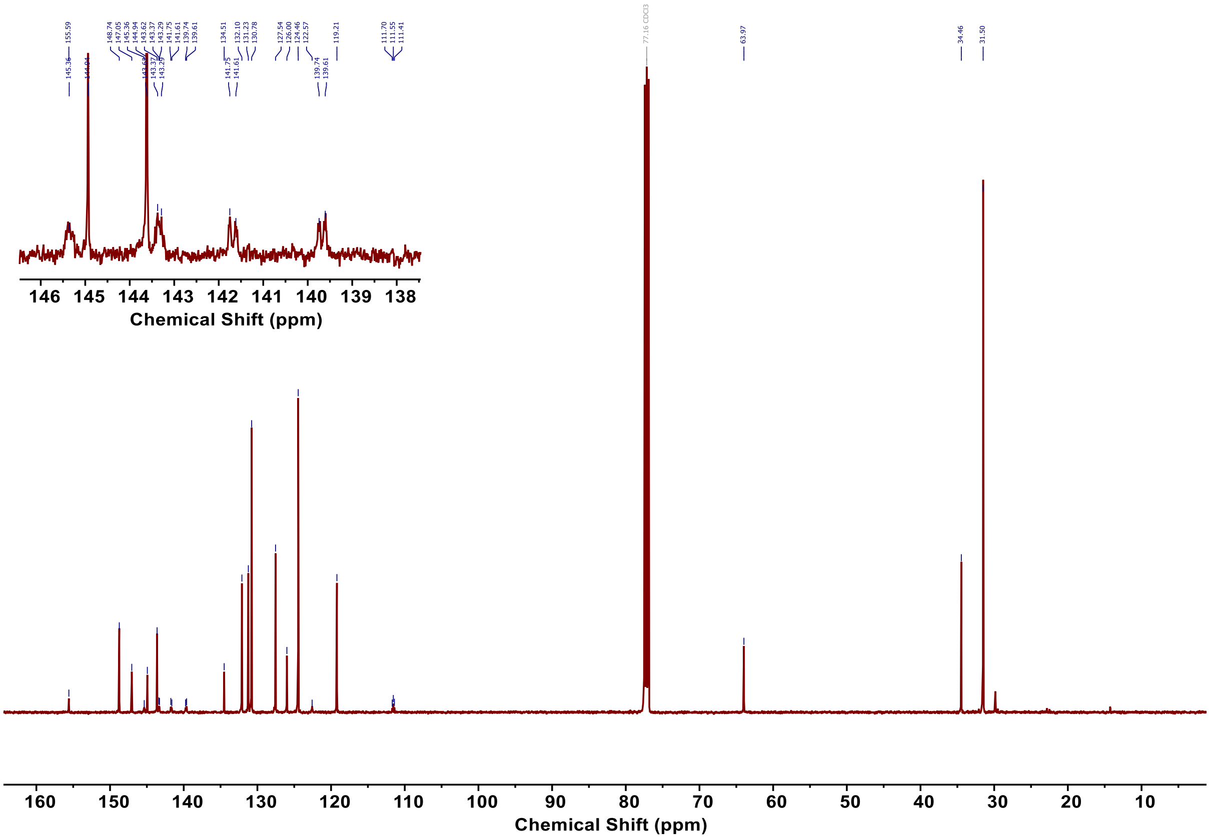

Figure S18. $^{13}C$ NMR spectrum of **9** ($CDCl_3$, 298 K, 126 MHz).

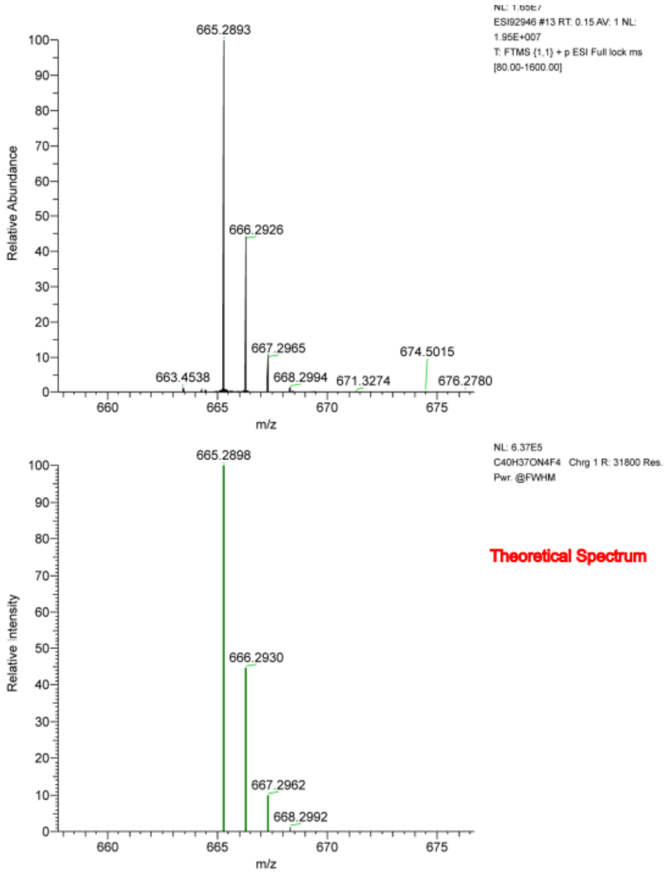

Figure S19. HRESI-MS of **9**.

**2·XB**

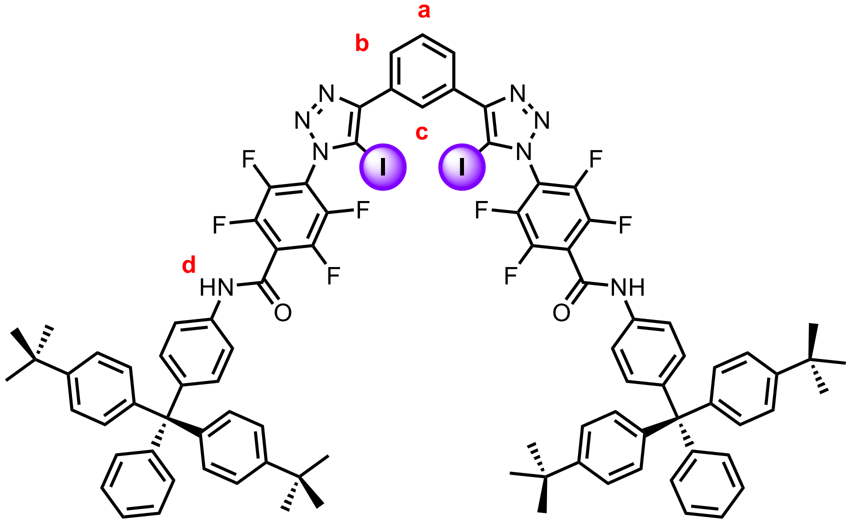


$[Cu(MeCN)_4]PF_6$ (28 mg, 0.0752 mmol) and TBTA (40 mg, 0.0752 mmol) were dissolved in anhydrous degassed $CH_2Cl_2$ (5 ml) and left to stir at room temperature under an atmosphere of nitrogen for 10 minutes. After which time, **9** (250 mg, 0.376 mmol) and **5** (68 mg, 0.179 mmol) were added as solids to the mixture and left to stir overnight. The reaction mixture was diluted with $CH_2Cl_2$ (50 ml) and the organic phase washed with 0.1 M $NH_4OH/EDTA_{(aq)}$ (20 ml) and $H_2O$ (20 ml) and dried over $MgSO_4$ and concentrated to dryness *in vacuo*. The crude solid was subjected to purification by silica gel column chromatography ($EtOAc:CH_2Cl_2$) to afford **2·XB** as a white solid (247 mg, 0.145 mmol, 81 %).

**$^1$H NMR** (500 MHz, $CDCl_3$) δ 9.05 (s, $2H_d$), 8.37 (s, $1H_c$), 7.79 (d, $J$ = 7.6 Hz, $2H_b$), 7.36 (d, $J$ = 8.5 Hz, 4H), 7.31 – 7.02 (m, 31H), 1.33 (s, 36H).

**$^{19}$F NMR** (470 MHz, $CDCl_3$) δ -138.50 (dd, $J$ = 23.3, 11.5 Hz), -141.99 (d, $J$ = 20.6 Hz).

**$^{13}$C NMR** (126 MHz, $CDCl_3$) δ 155.14, 150.98, 148.81, 147.03, 144.91, 143.61, 134.50, 132.00, 131.23, 130.78, 130.22 – 128.94 (m), 127.54, 126.05, 124.48, 119.50, 63.97, 34.47, 31.50, 29.85. Some peaks not observed due to C-F coupling.

**HRMS** (ESI+ve) m/z: 1707.4143 ($[M+H]^+$, $C_{90}H_{77}O_2N_8F_8I_2$ requires 1707.4126).

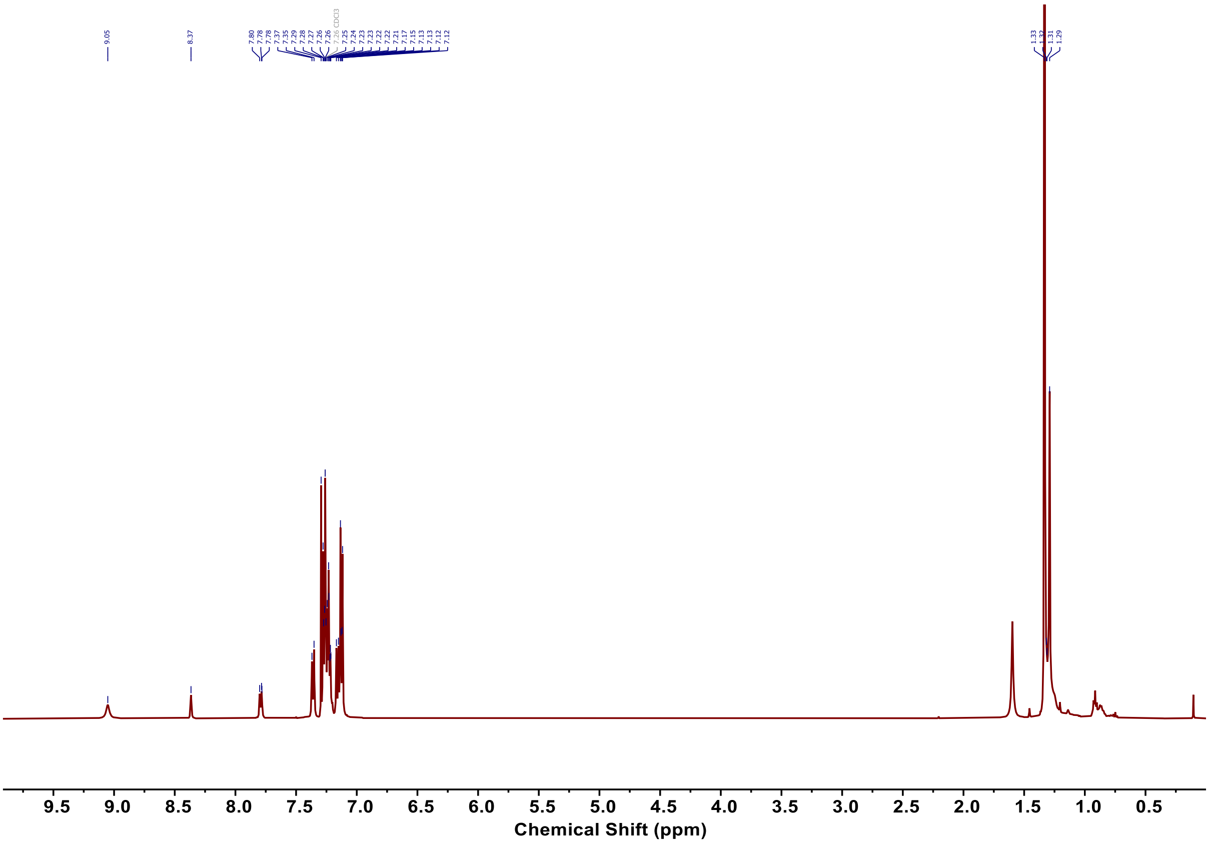

Figure S20. $^{1}H$ NMR spectrum of **2·XB** ($CDCl_3$, 298 K, 500 MHz).

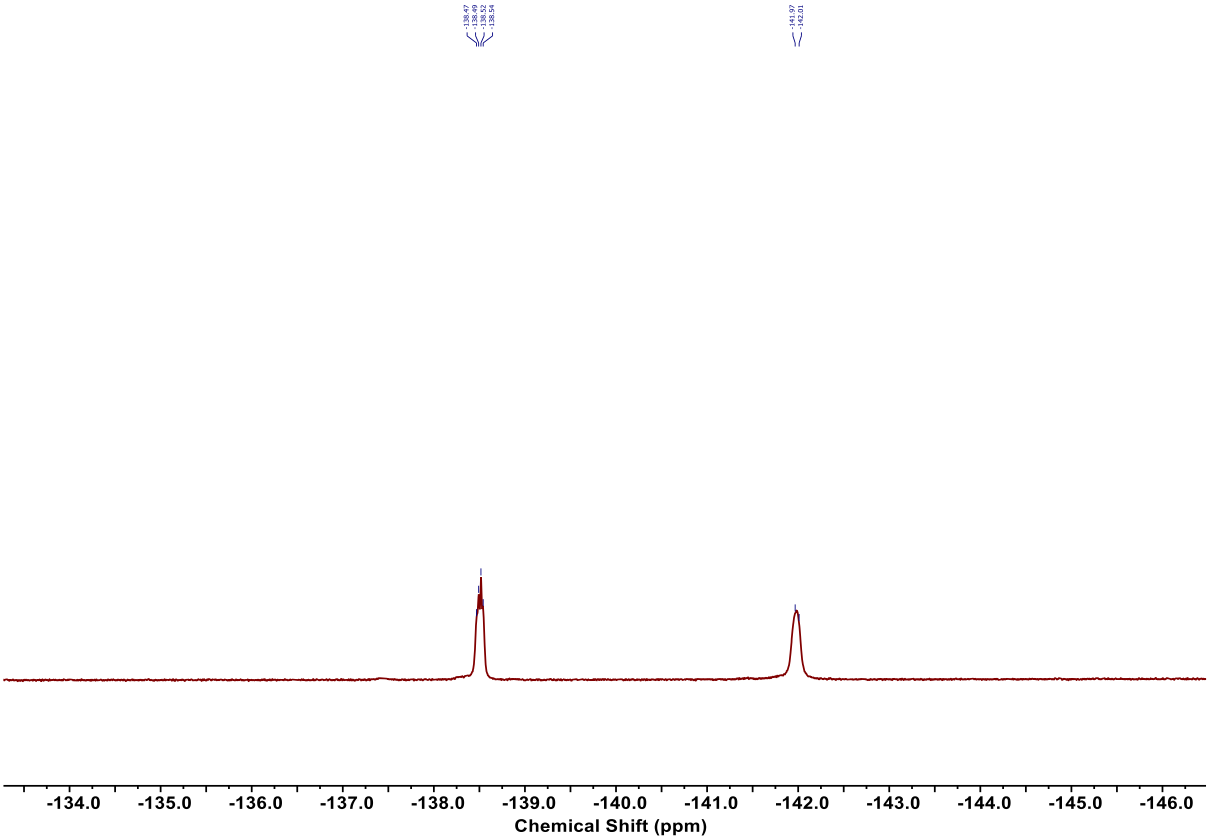

Figure S21. $^{19}F$ NMR spectrum of **2·XB** ($CDCl_3$, 298 K, 470 MHz).

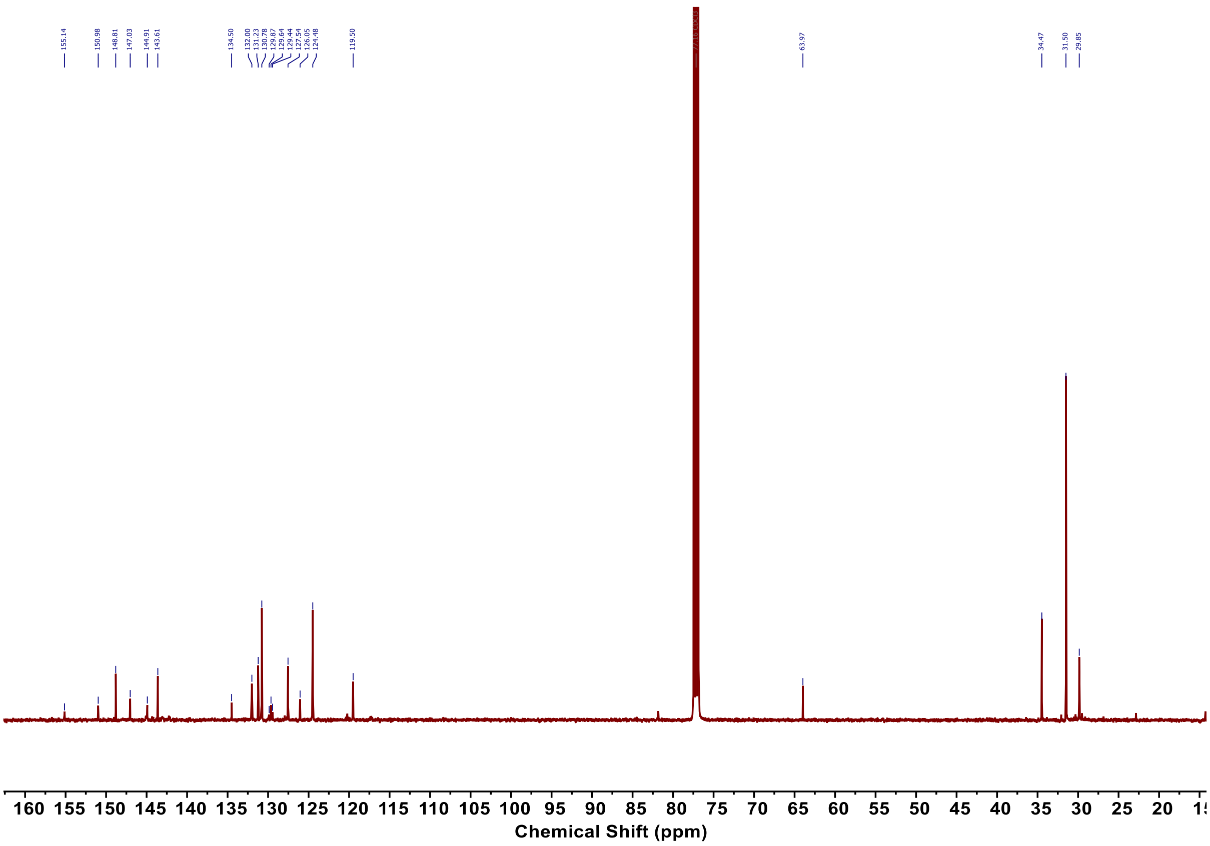


Figure S22. $^{13}$C NMR spectrum of **2·XB** ($CDCl_3$, 298 K, 126 MHz).

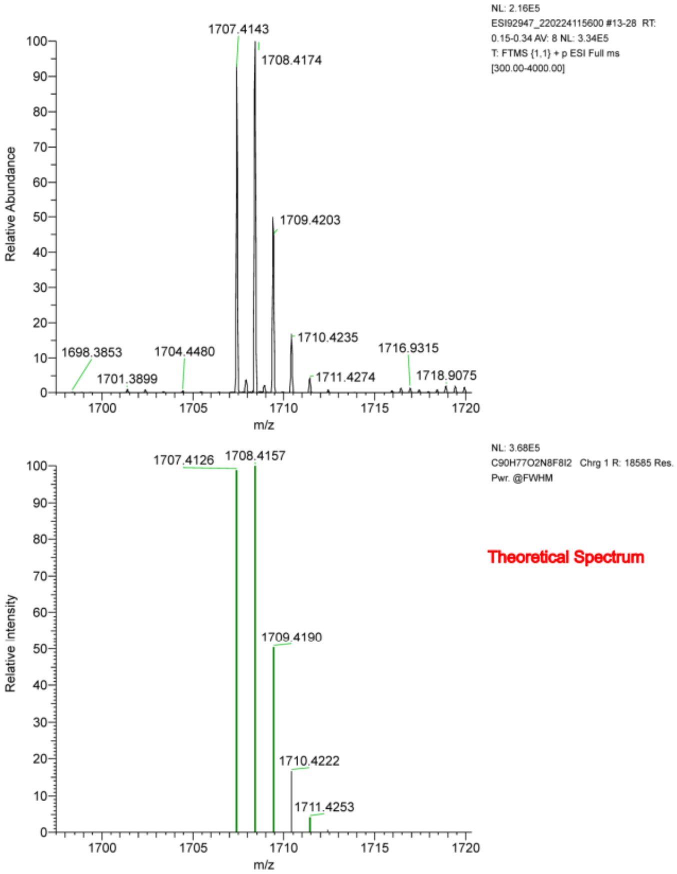


Figure S23. HRESI-MS of **2·XB**.

**2·HB**

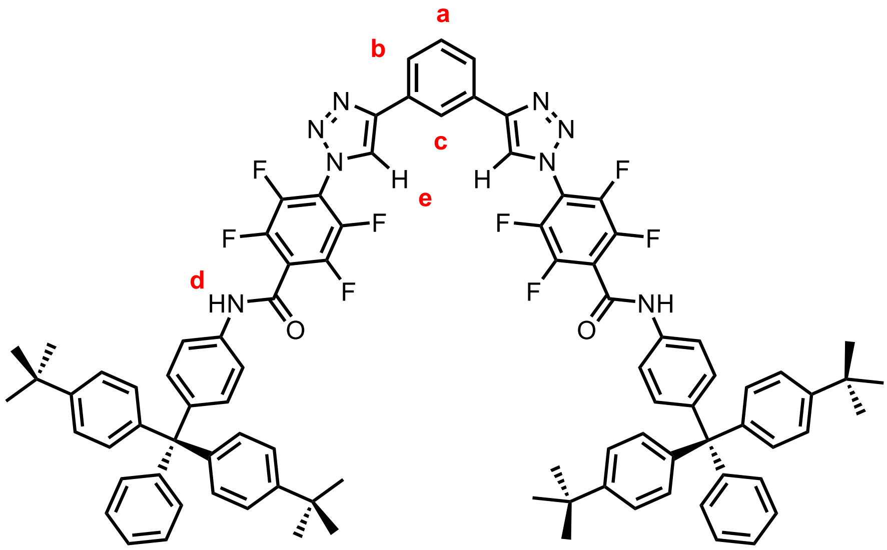

$[Cu(MeCN)_4]PF_6$ (28 mg, 0.0752 mmol) and TBTA (40 mg, 0.0752 mmol) were dissolved in anhydrous degassed $CH_2Cl_2$ (5 ml) and left to stir at room temperature under an atmosphere of nitrogen for 10 minutes. After which time, **9** (250 mg, 0.376 mmol) and **6** (23 mg, 0.179 mmol) were added to the mixture and left to stir overnight. The reaction mixture was diluted with $CH_2Cl_2$ (50 ml) and the organic phase washed with 0.1 M $NH_4OH/EDTA_{(aq)}$ (20 ml) and $H_2O$ (20 ml) dried over $MgSO_4$ and concentrated to dryness *in vacuo*. The crude solid was subjected to purification by silica gel column chromatography ($EtOAc:CH_2Cl_2$) to afford **2·HB** as a white solid (146 mg, 0.100 mmol, 56 %).

**$^1$H NMR** (500 MHz, $CDCl_3$) δ 8.92 (s, $2H_d$), 8.16 (s, $1H_c$), 8.07 (s, $2H_e$), 7.79 (d, *J* = 7.7 Hz, $2H_b$), 7.57 (d, *J* = 8.3 Hz, 4H), 7.48 (t, *J* = 7.7 Hz, $1H_a$), 7.28 – 7.00 (m, 28H), 1.29 (s, 36H).

**$^{19}$F NMR** (470 MHz, $CDCl_3$) δ -138.21, -144.80.

**$^{13}$C NMR** (126 MHz, $CDCl_3$) δ 154.87, 148.75, 147.39, 147.04, 145.08, 143.61, 134.72, 132.14, 131.21, 130.77, 130.22, 129.65, 127.54, 126.60, 126.00, 124.47, 122.40, 119.29, 63.99, 34.46, 31.50.

**HRMS** (ESI+ve) m/z: 1456.6221 ($[M+H]^+$, $C_{90}H_{79}O_2N_8F_8I_2$ requires 1456.6225).

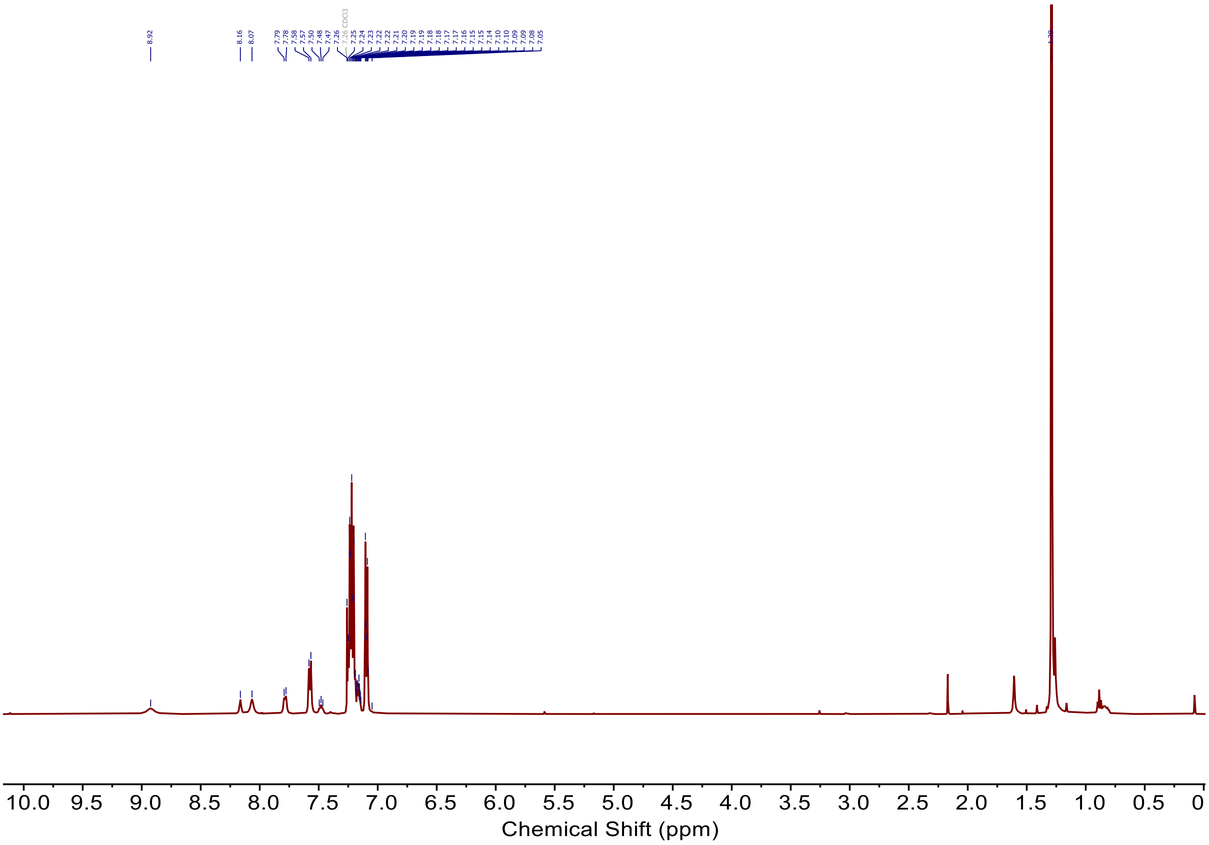


Figure S24. $^{1}$H NMR spectrum of **2·HB** ($CDCl_3$, 298 K, 500 MHz).

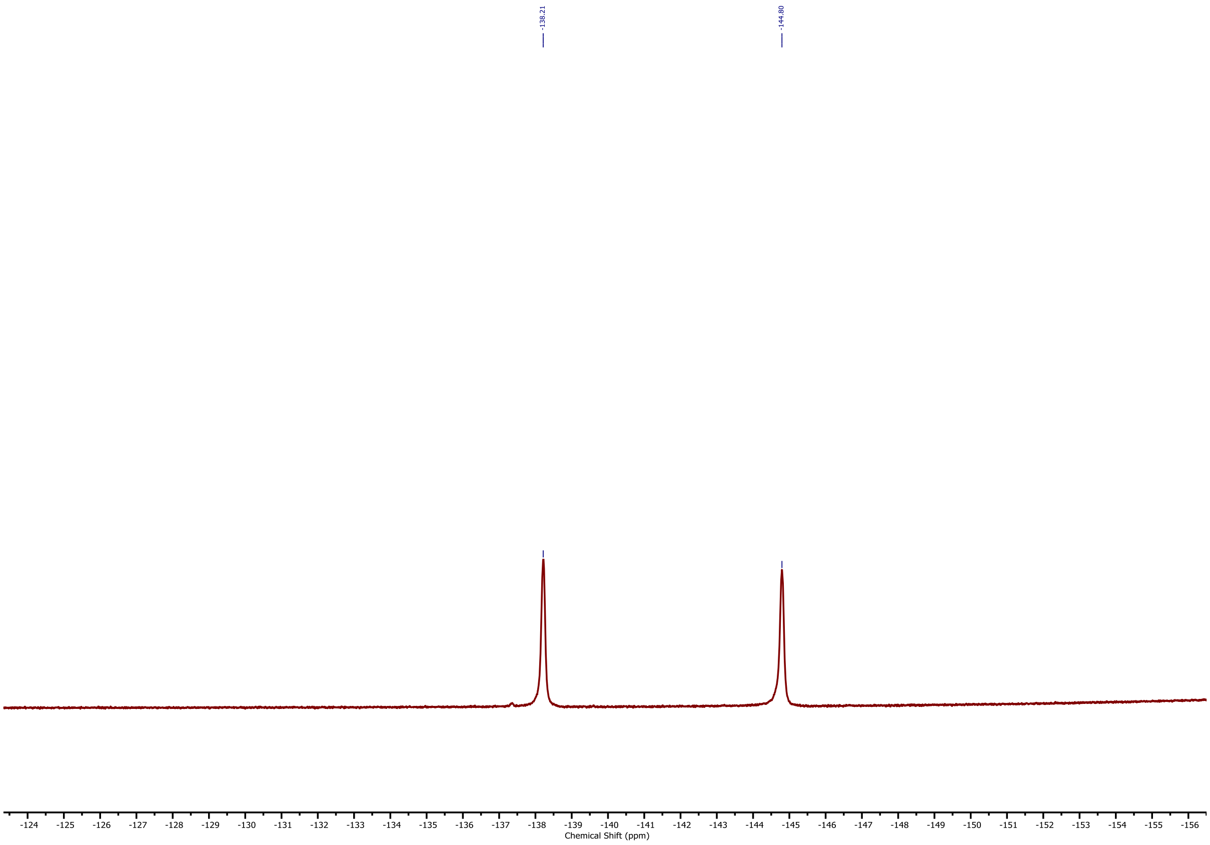


Figure S25. $^{19}$F NMR spectrum of **2·HB** ($CDCl_3$, 298 K, 470 MHz).

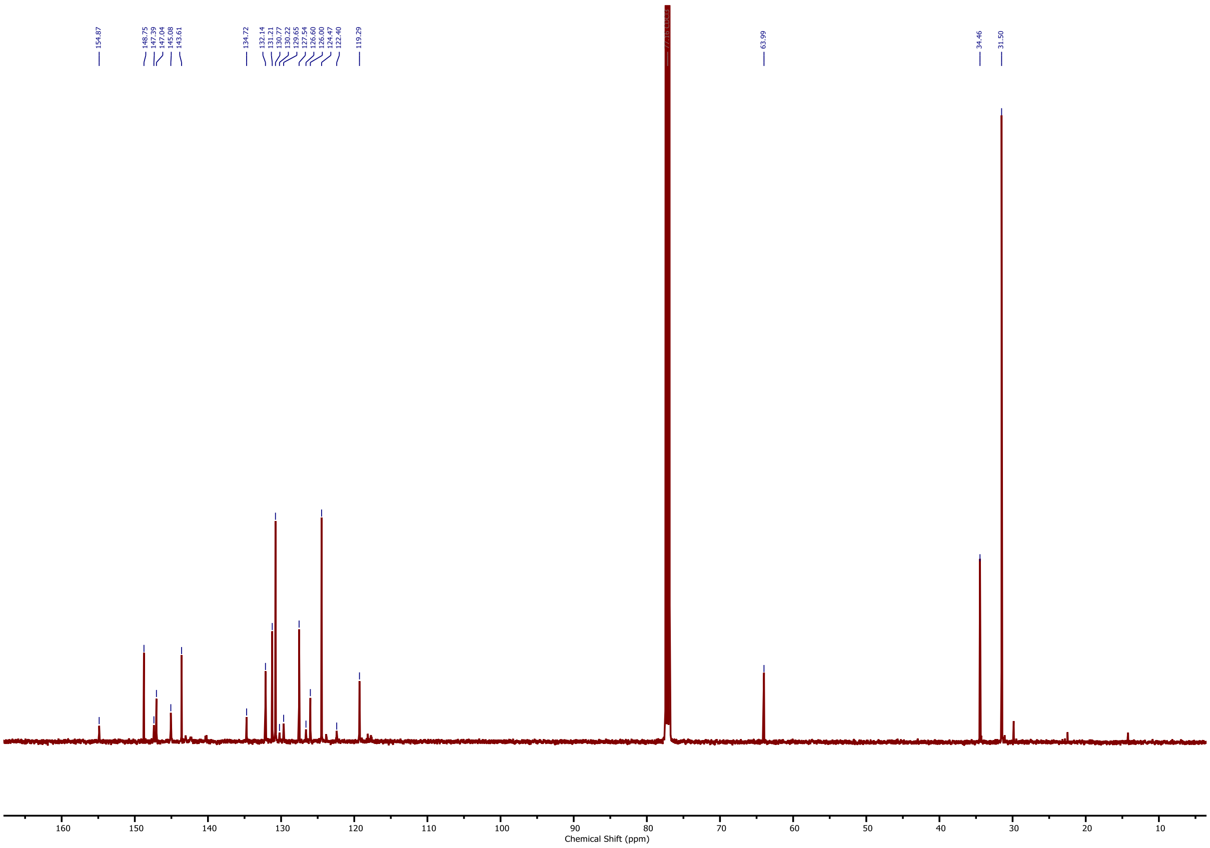


Figure S26. $^{13}C$ NMR spectrum of **2·HB** ($CDCl_3$, 298 K, 126 MHz).

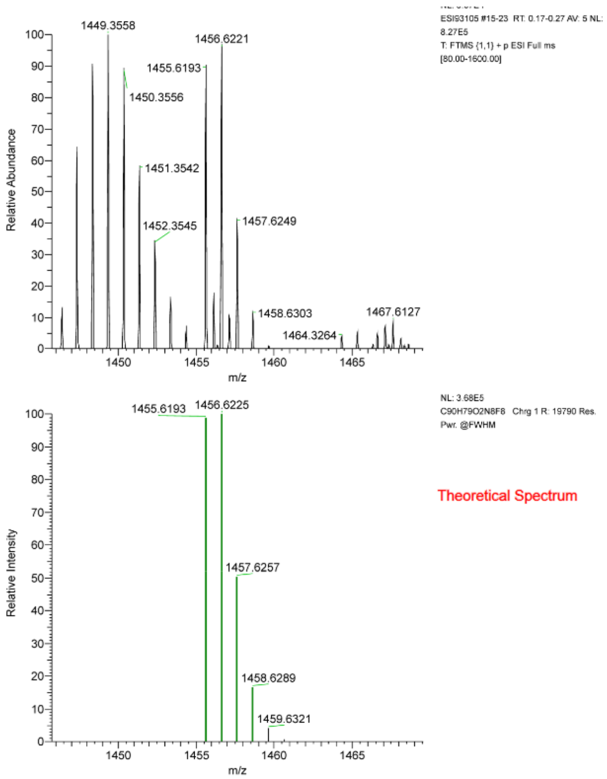


Figure S27. HRESI-MS of **2·HB**.

**3·XB**

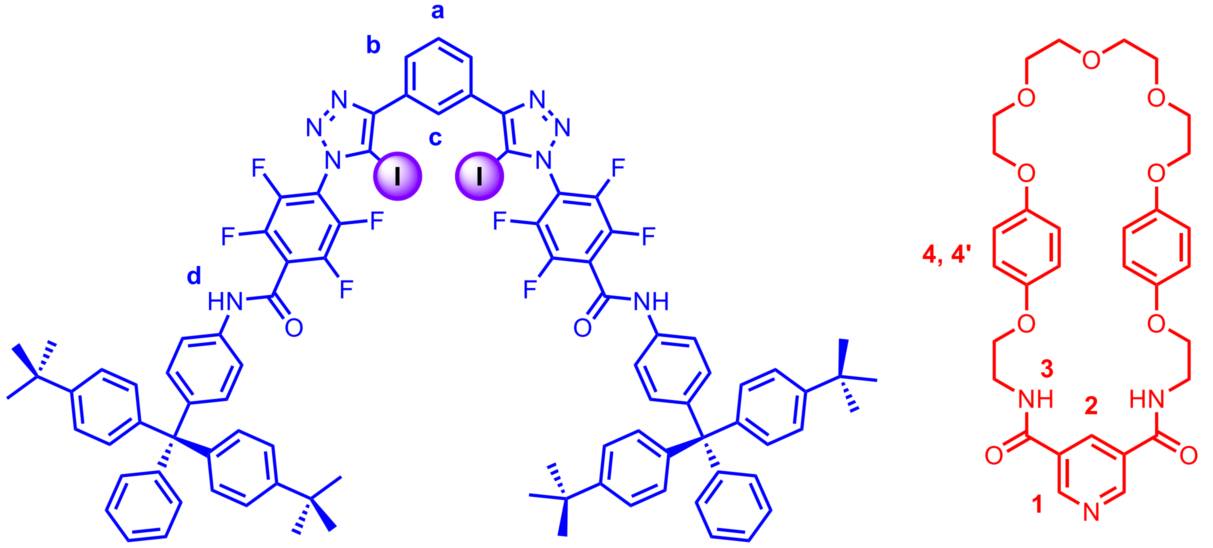


3,5-Pyridinedicarboxylic acid (49 mg, 0.293 mmol) was suspended in neat oxalyl chloride (2 ml), to which was added one drop of DMF. After stirring for 2 hours, the mixture became homogenous, at which point the volatiles were removed *in vacuo* to afford the corresponding bis-acid chloride **11**. In a separate flask, **2·XB** (50 mg, 0.0293 mmol), **10** (136 mg, 0.293 mmol), TBACl (8 mg, 0.0293 mmol) and $NEt_3$ (0.1 ml) were dissolved in anhydrous $CH_2Cl_2$ (12 ml) and cooled to 0 °C. Bis-acid chloride **11** was dissolved in anhydrous $CH_2Cl_2$ (3 ml) and added dropwise to a solution of **2·XB**, the mixture was allowed to warm to room temperature and stirred overnight. The reaction mixture was diluted with $CH_2Cl_2$ (50 ml) and washed with sat. $Na_2CO_{3(aq)}$ (20 ml) and $H_2O$ (20 ml), the collected organic phase was dried over $MgSO_4$, concentrated to dryness *in vacuo* and purified by preparative thin layer chromatography (EtOAC:$CH_2Cl_2$:MeOH) and isolated as a white solid (11 mg, 4.98 µmol, 17 %).

**$^1$H NMR** (500 MHz, acetone-$d_6$) δ 10.31 (s, $2H_d$), 9.14 (s, $2H_1$), 8.78 (s, $1H_c$), 8.60 (s, $1H_2$), 8.28 (s, $1H_3$), 8.19 (dd, *J* = 7.8, 1.8 Hz, $1H_b$), 7.80 (t, *J* = 7.8 Hz, $1H_a$), 7.68 (d, *J* = 8.4 Hz, 4H), 7.46 – 7.04 (m, 30H), 6.60 – 6.30 (m, $8H_{4,4'}$), 4.01 (t, *J* = 5.2 Hz, 4H), 3.88 – 3.68 (m, 4H), 3.68 – 3.50 (m, 16H), 1.31 (s, 36H).

**$^{19}$F NMR** (470 MHz, acetone-$d_6$) δ -140.41, -143.42 – -144.59 (m).

**$^{13}$C NMR** (126 MHz, acetone-$d_6$) δ 165.78, 155.47, 154.28, 153.99, 151.89, 150.75, 149.44, 148.00, 145.03, 144.75, 136.67, 136.57, 133.53, 132.51, 131.75, 131.45, 131.26, 130.44, 128.72, 128.42, 126.82, 126.63, 125.30, 119.71, 119.62, 116.39, 116.29, 83.51, 71.45, 70.39, 68.95, 67.85, 64.72, 40.46, 34.91, 31.64. Some peaks not observed due to C-F coupling.

**HRMS** (ESI+ve) m/z: 2303.6664 ($[M+H]^+$, $C_{121}H_{114}O_{11}N_{11}F_8I_2$ requires 2303.6687).

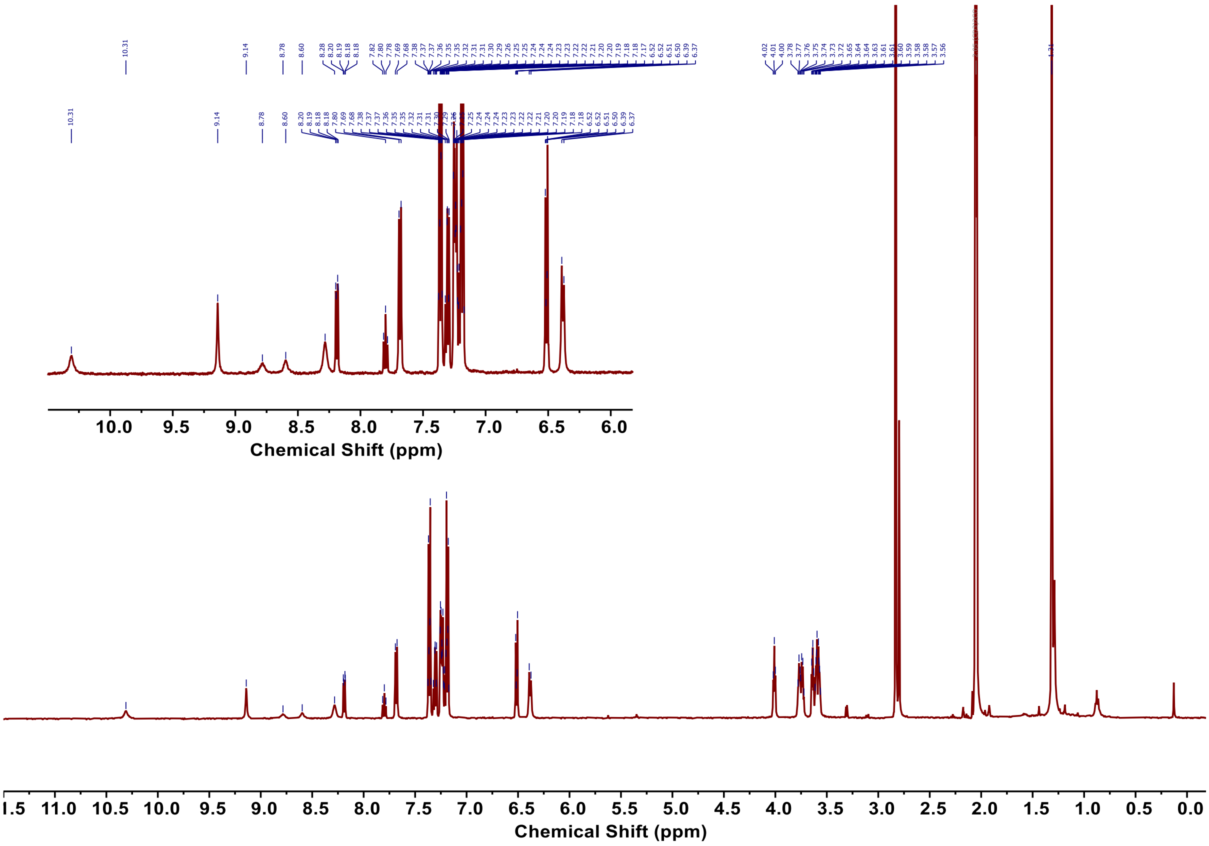


Figure S28. $^{1}H$ NMR spectrum of **3·XB** (acetone-$d_6$, 298 K, 500 MHz).

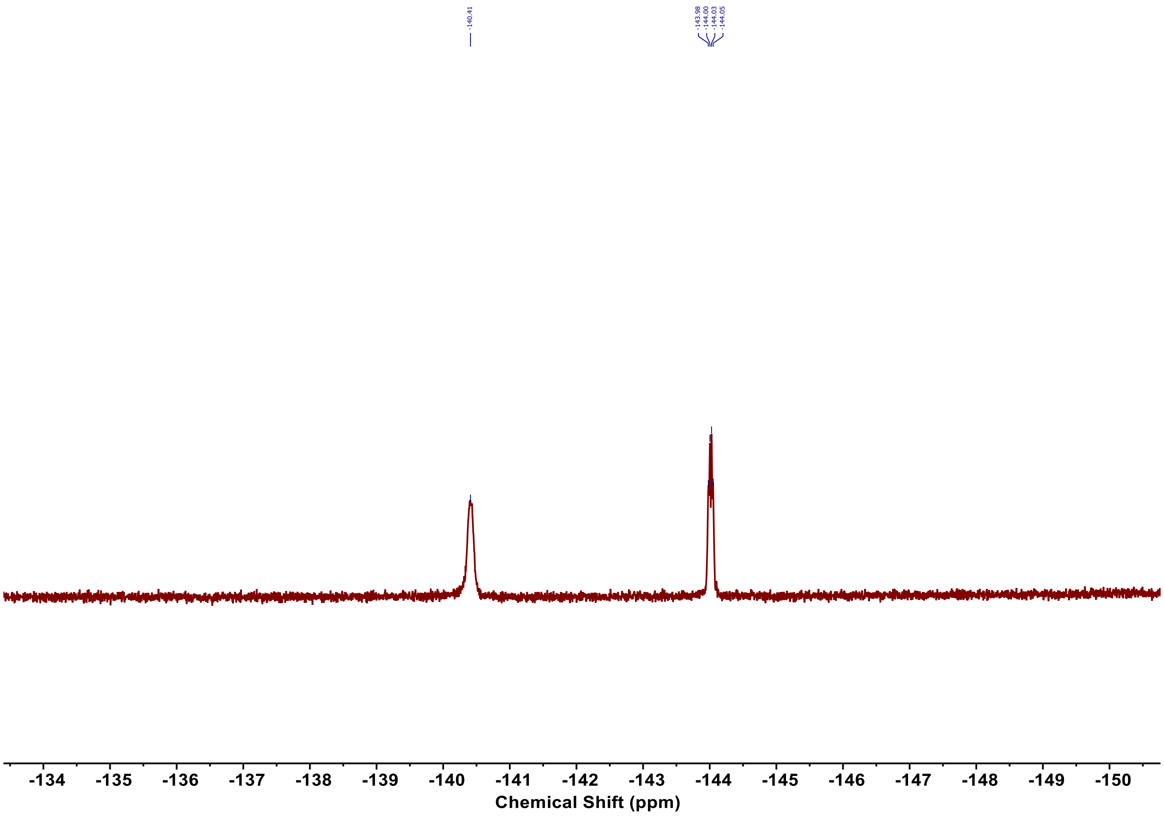


Figure S29. $^{19}F$ NMR spectrum of **3·XB** (acetone-$d_6$, 298 K, 470 MHz).

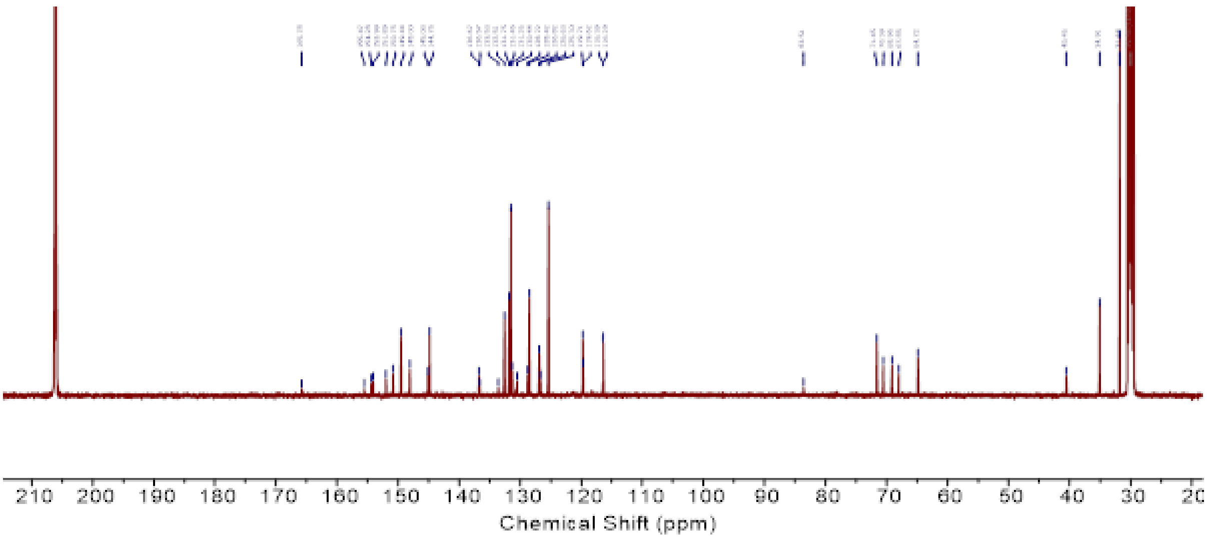


Figure S30. $^{13}$C NMR spectrum of **3·XB** (acetone-$d_6$, 298 K, 126 MHz).

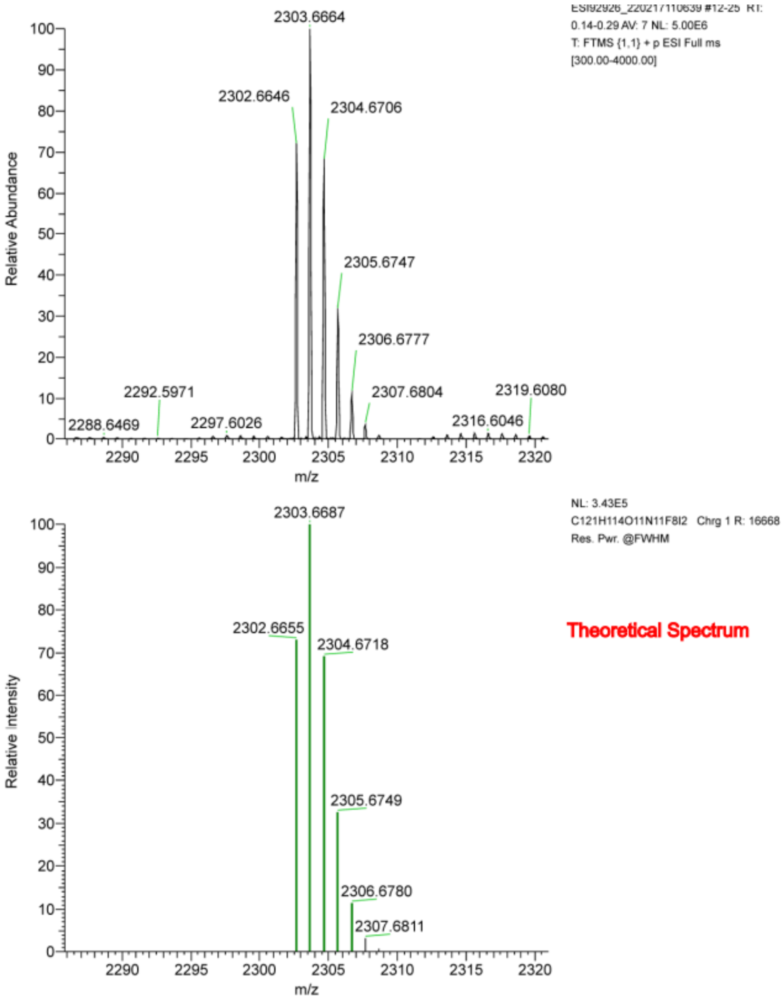


Figure S31. HRESI-MS of **3·XB**.

**2·XB$^{(NO2)2}$**

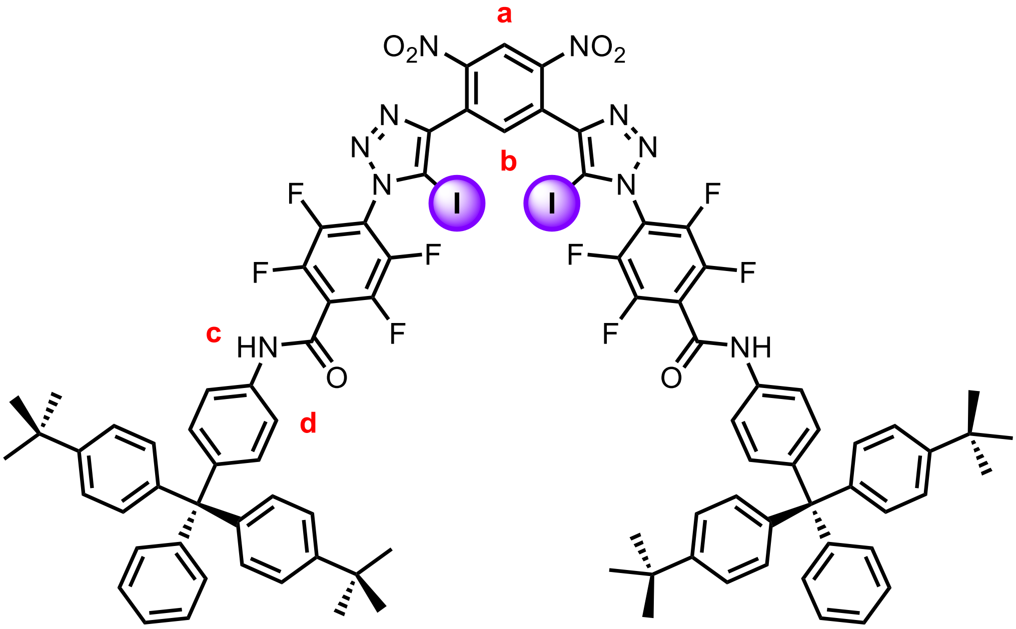


$[Cu(MeCN)_4]PF_6$ (28 mg, 0.0752 mmol) and TBTA (40 mg, 0.0752 mmol) were dissolved in anhydrous degassed $CH_2Cl_2$ (5 ml) and left to stir at room temperature under an atmosphere of nitrogen for 10 minutes. After which time, **9** (250 mg, 0.376 mmol) and **4** (84 mg, 0.179 mmol) were added as solids to the mixture and left to stir overnight. The reaction mixture was diluted with $CH_2Cl_2$ (50 ml) and the organic phase washed with 0.1 M $NH_4OH$/$EDTA_{(aq)}$ (20 ml) and $H_2O$ (20 ml) dried over $MgSO_4$ and concentrated to dryness *in vacuo*. The crude solid was subjected to purification by silica gel column chromatography (EtOAc:$CH_2Cl_2$) to afford **2·XB$^{(NO2)2}$** as a yellow solid (219 mg, 0.122 mmol, 68 %).

**$^1$H NMR** (500 MHz, $CDCl_3$) δ 8.85 (s, $1H_a$), 8.13 (s, $1H_b$), 8.06 (s, $2H_c$), 7.47 (d, *J* = 8.7 Hz, $4H_d$), 7.31 – 7.04 (m, 30H), 1.31 (s, 32H).

**$^{19}$F NMR** (470 MHz, $CDCl_3$) δ -137.16 – -137.79 (m), -140.14 – -141.12 (m).

**$^{13}$C NMR** (126 MHz, $CDCl_3$) δ 154.59, 148.86, 148.43, 147.14, 146.98, 145.42, 143.54, 137.53, 134.26, 132.21, 131.24, 130.78, 128.83, 127.59, 126.09, 124.52, 122.60, 119.26, 84.73, 64.00, 34.48, 31.50, 29.85. Some peaks not resolved due to C-F coupling.

**HRMS** (ESI+ve) m/z: 1797.3845 ($[M+H]^+$, $C_{90}H_{75}F_8N_{10}I_2$ requires 1797.3833).

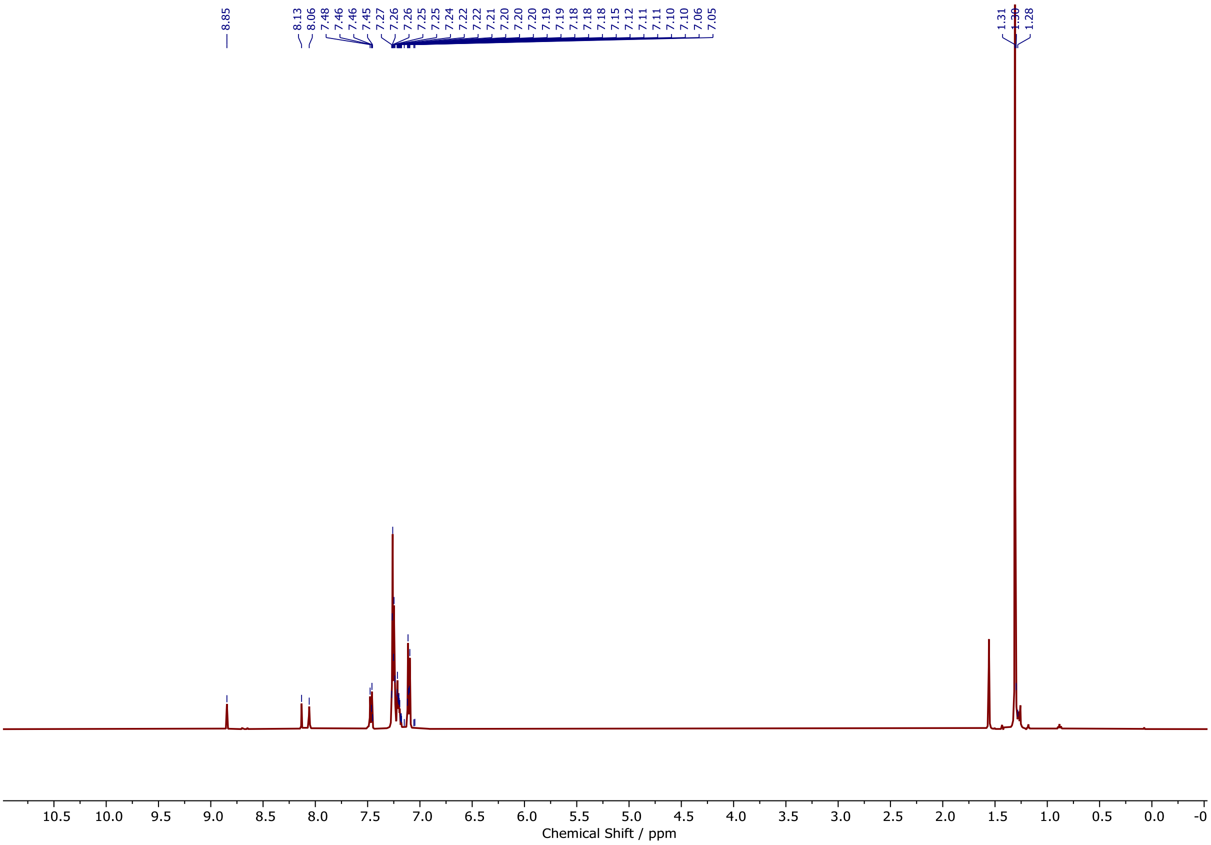


Figure S32. $^{1}H$ NMR spectrum of **2·XB$^{(NO2)2}$** ($CDCl_3$, 298 K, 500 MHz).

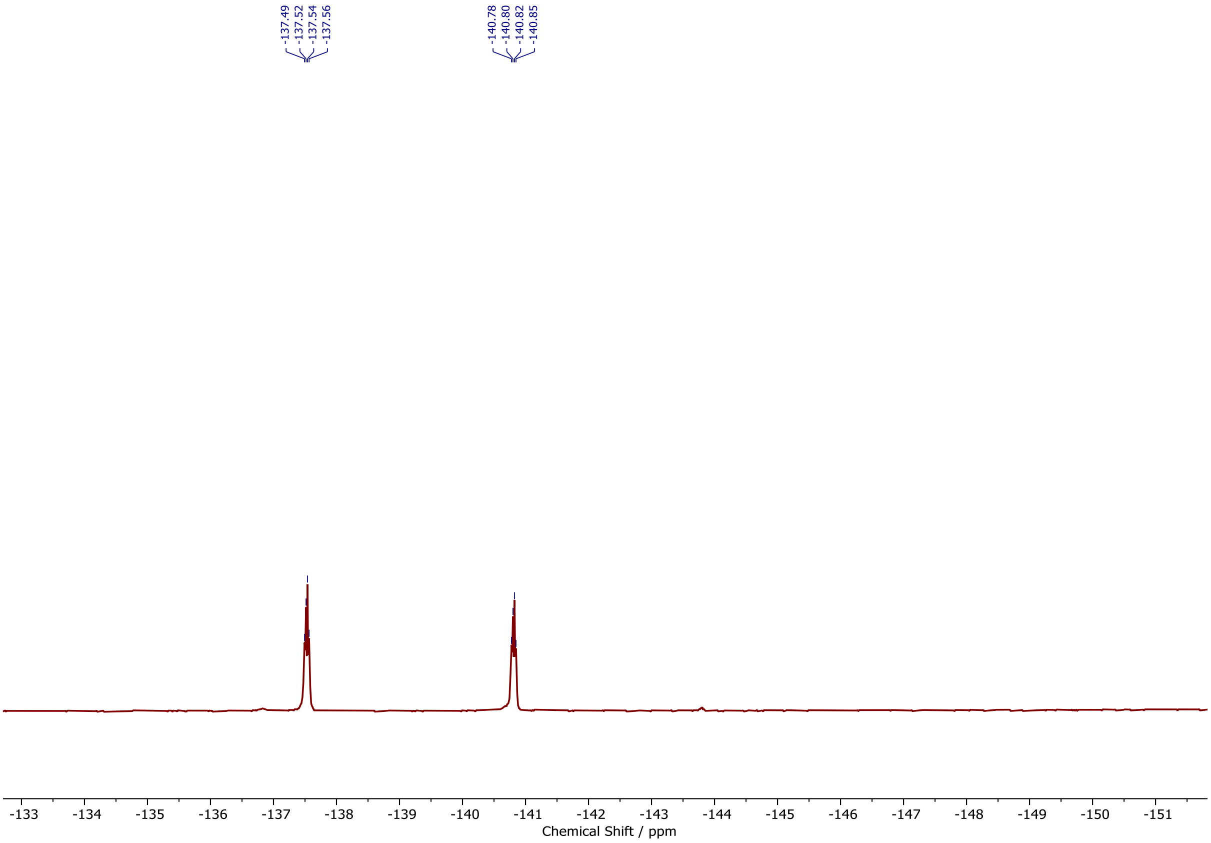


Figure S33. $^{19}F$ NMR spectrum of **2·XB$^{(NO2)2}$** ($CDCl_3$, 298 K, 470 MHz).

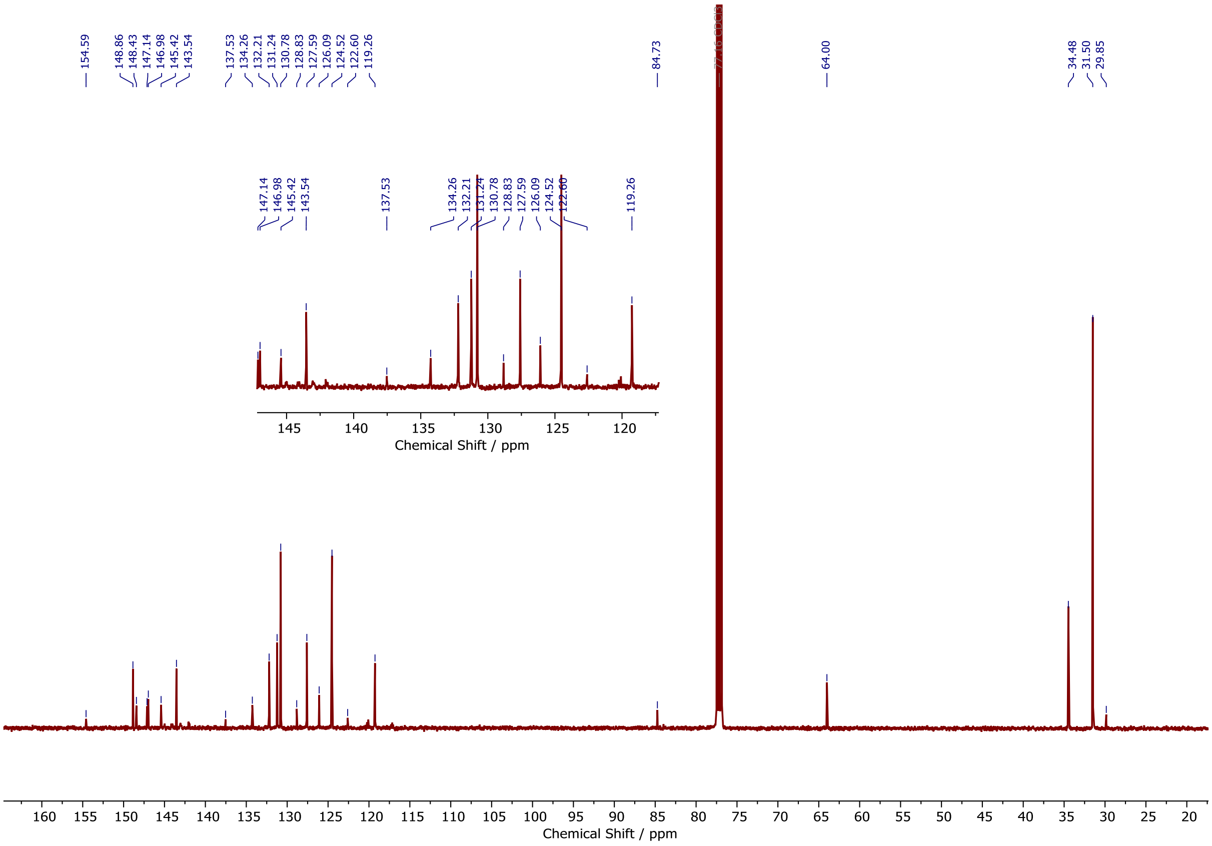


Figure S34. $^{13}$C NMR spectrum of **2·XB$^{(NO2)2}$** ($CDCl_3$, 298 K, 126 MHz).

**3·XB$^{(NO2)2}$**

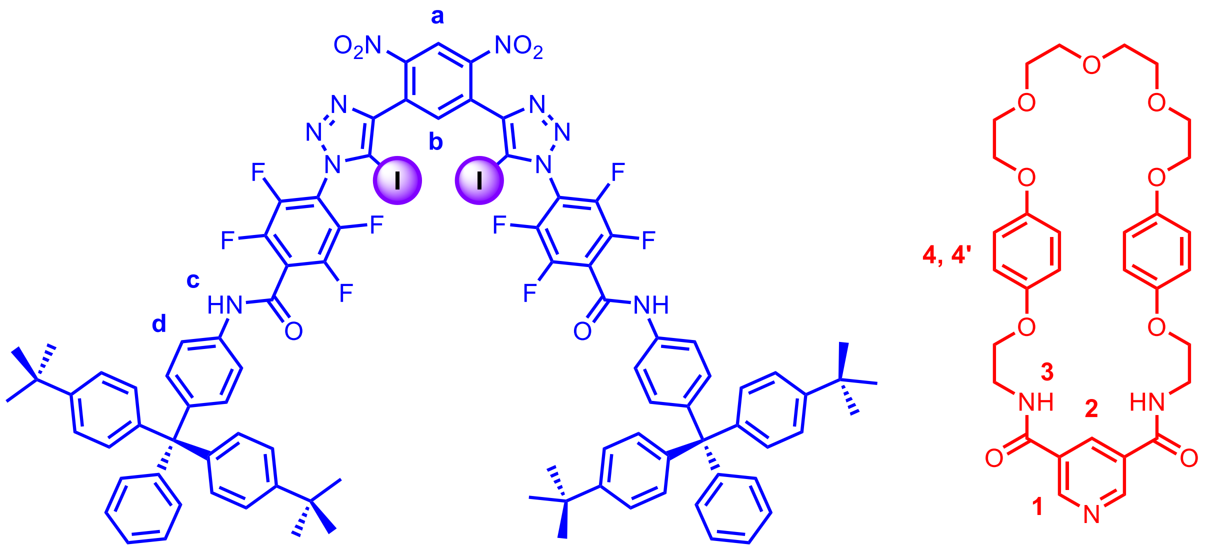


3,5-Pyridinedicarboxylic acid (50 mg, 0.278 mmol) was suspended in neat oxalyl chloride (2 ml), to which was added one drop of DMF. After stirring for 2 hours, the mixture became homogenous, at which point the volatiles were removed *in vacuo* to afford the corresponding bis-acid chloride **11**. In a separate flask, **2·XB$^{(NO2)2}$** (50 mg, 0.0278 mmol), **10** (129 mg, 0.278 mmol), TBACl (7.7 mg, 0.0278 mmol) and $NEt_3$ (0.1 ml) were dissolved in anhydrous $CH_2Cl_2$ (12 ml) and cooled to 0 °C. Bis-acid chloride **11** was dissolved in anhydrous $CH_2Cl_2$ (3 ml) and added dropwise to solution of **2·XB$^{(NO2)2}$**, the mixture was allowed to warm to room temperature and stirred overnight. The reaction mixture was diluted with $CH_2Cl_2$ (50 ml) and washed with sat. $Na_2CO_{3(aq)}$ (20 ml) and $H_2O$ (20 ml), the collected organic phase was dried over $MgSO_4$, concentrated to dryness *in vacuo* and purified by preparative thin layer chromatography (EtOAc:$CH_2Cl_2$:MeOH) and isolated as a white solid (24.6 mg, 10.3 μmol, 37 %).

**$^1$H NMR** (500 MHz, acetone-$d_6$) δ 10.25 (s, $2H_c$), 9.14 (s, $2H_1$), 9.10 (s, $1H_a$), 8.67 (s, $1H_2$) 8.26 (s, $1H_b$), 7.99 (s, $2H_3$), 7.67 (d, $J$ = 8.4 Hz, $4H_d$), 7.44 – 7.07 (m, 30H), 6.63 – 6.27 (m, $8H_{4,4'}$), 4.02 (t, $J$ = 5.2 Hz, 4H), 3.86 – 3.41 (m, 20H), 1.31 (s, 36H).

**$^{13}$C NMR** (151 MHz, acetone-$d_6$) δ 165.79, 155.24 (d, $J$ = 14.3 Hz), 153.67 (d, $J$ = 3.0 Hz), 151.46, 149.47 (d, $J$ = 3.0 Hz), 149.40, 148.04, 147.64, 144.91, 144.83, 136.60 (d, $J$ = 14.9 Hz), 134.67, 132.36, 131.75, 131.47, 130.73, 128.43, 126.79, 125.31, 119.89 (d, $J$ = 12.1 Hz), 116.36 (d, $J$ = 13.2 Hz), 115.83 (d, $J$ = 3.3 Hz), 71.45, 71.35, 70.40, 70.28, 68.97, 68.26, 67.87, 67.57, 64.74.

**HRMS** (ESI+ve) m/z: 2393.6362 ($[M+H]^+$, $C_{121}H_{112}F_8I_2O_{15}N_{13}$ requires 2393.6389).

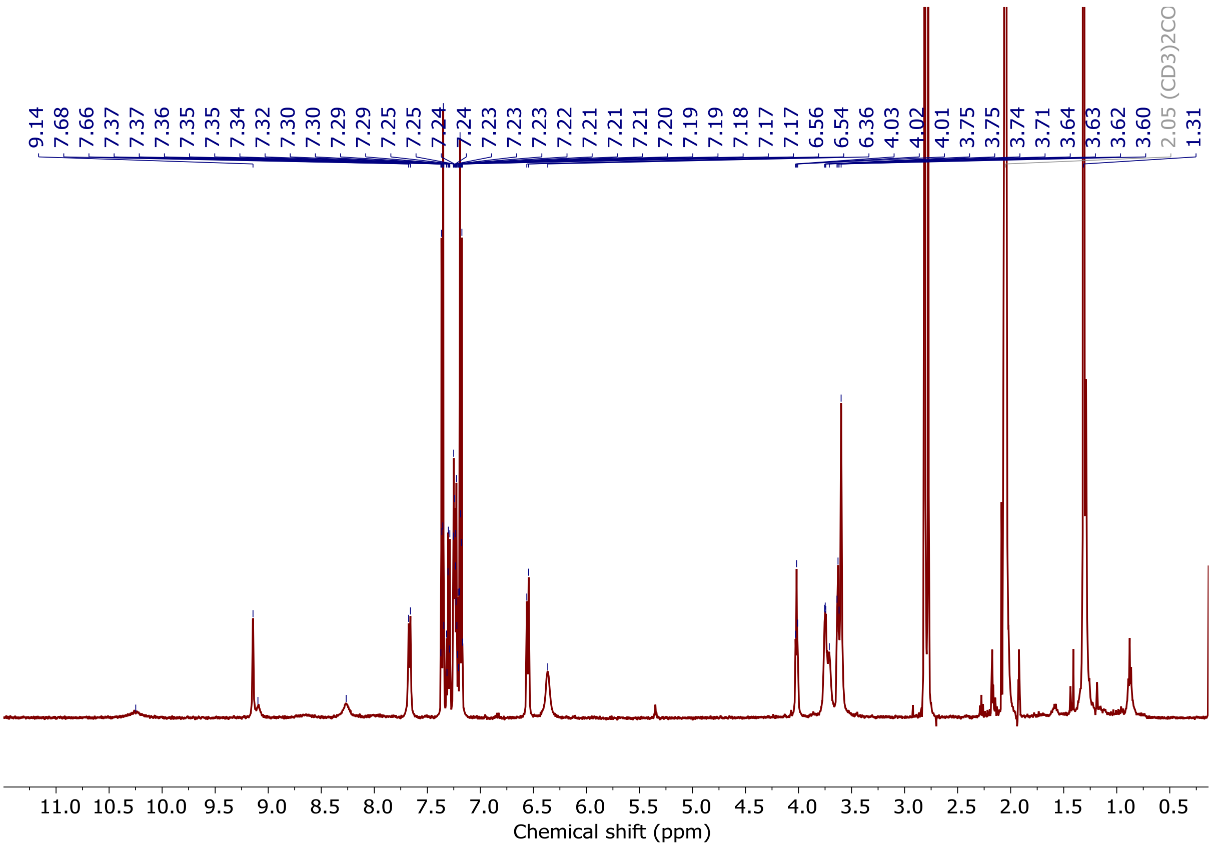


Figure S35. $^{1}$H NMR spectrum of **3·XB$^{(NO2)2}$** (acetone-d$_6$, 298 K, 500 MHz).

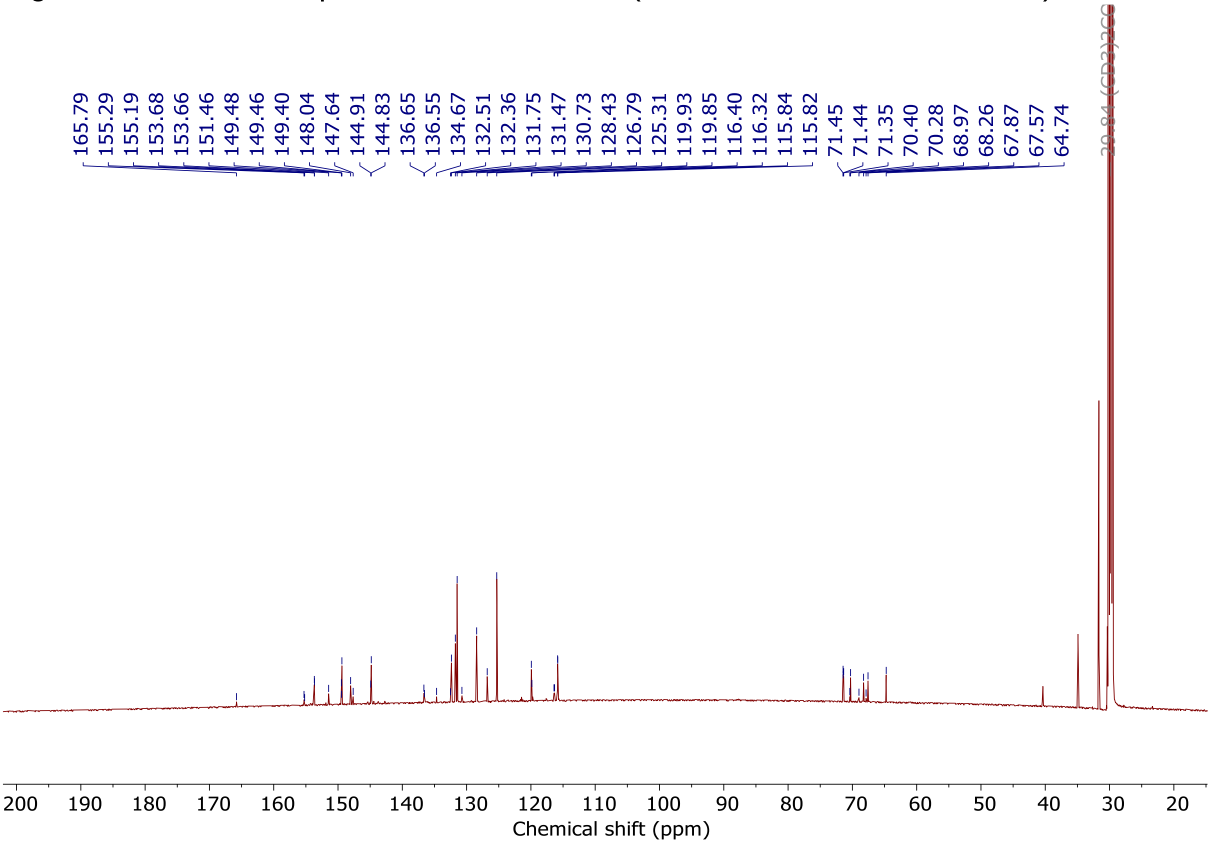


Figure S36. $^{13}$C NMR spectrum of **3·XB$^{(NO2)2}$** (acetone-d$_6$, 298 K, 151 MHz).

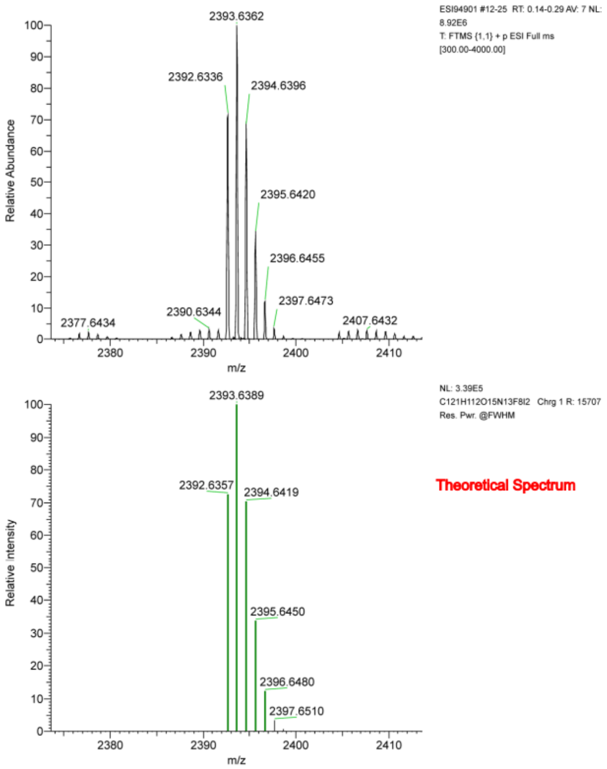


Figure S37. $^{1}$H NMR spectrum of **3·XB$^{(NO2)2}$**.

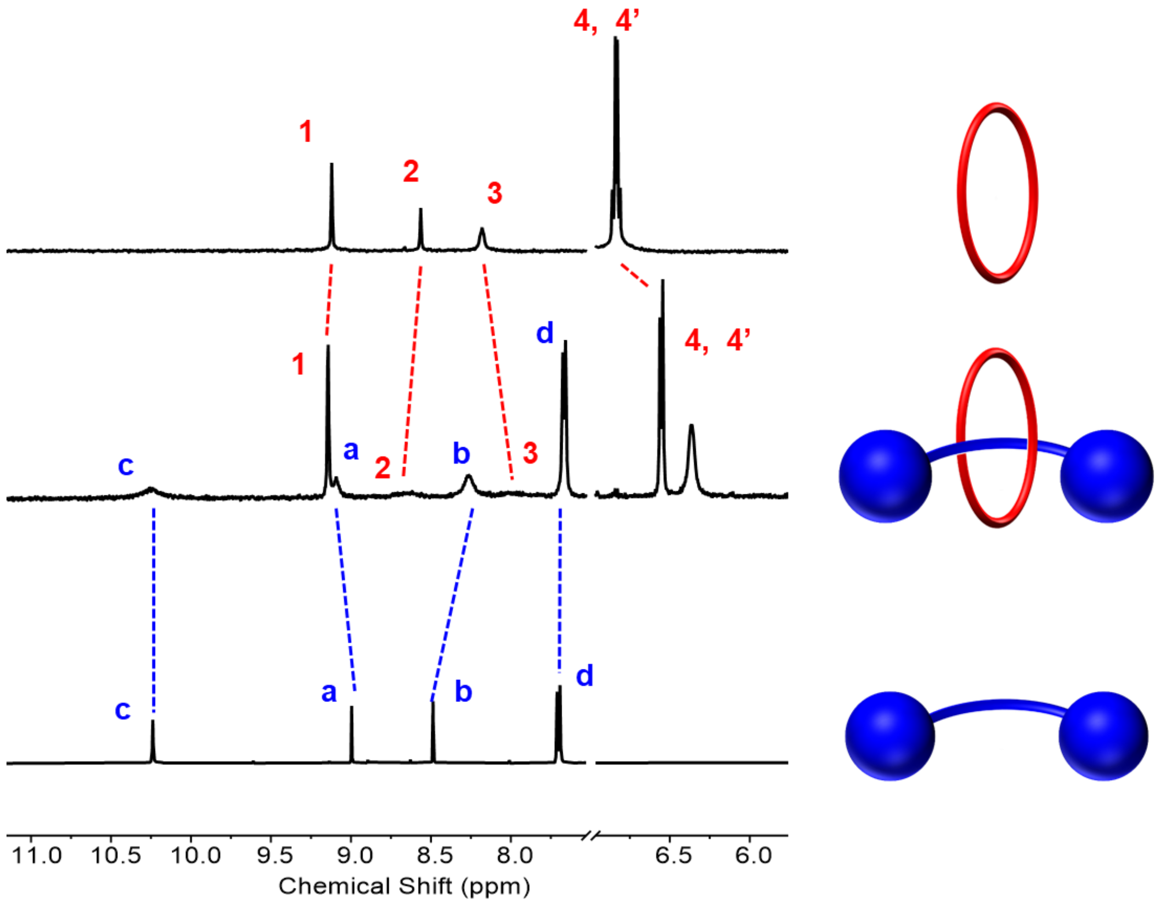


Figure S38. Truncated stacked $^{1}$H NMR spectra of macrocycle **12** (top) [2]rotaxane **3·XB$^{(NO2)2}$** (middle) and **2·XB$^{(NO2)2}$** axle (bottom) (acetone-$d_6$, 500 MHz, 298 K).

## 3. $^{1}$H NMR Anion Recognition Studies

### $^{1}$H NMR anion titration protocol

Titration protocol: In a typical $^{1}$H NMR anion titration experiment, aliquots of anion were added to the $D_2O$: Acetone-$d_6$ solution of the receptor and the spectrum recorded.

For **1·XB**, **1·XB$^{(NO2)2}$** and **1·HB**:
[Host] = 1.0 mM
[TBACl] = 50 mM

For **2·XB**, **3·XB**, **2·XB$^{(NO2)2}$** and **3·XB$^{(NO2)2}$**:
[Host] = 0.5 mM
[TBACl] or [TBABr] or [TBAI] = 25 mM

Spectra were recorded at 0, 0.2, 0.4, 0.6, 0.8, 1.0, 1.2, 1.4, 1.6, 1.8, 2.0, 2.5, 3.0, 4.0, 5.0, 7.0 and 10 equivalents.

## Discussion regarding chemical shift perturbations observed during anion titration experiments

In general, during the course of $^{1}H$ NMR anion titration experiments, the addition of aliquots of anion induces a downfield shift of those signals associated with the anion binding site – most significantly when those environments form strong hydrogen bonding-anion interactions, *e.g.* N-H, O-H or C-H. This is typically rationalised by further polarisation of the E-H bond by the proximal anion. However, a notable exception is an example by Smith *et al.* wherein the magnetic anisotropy of the nitrate anion induced upfield perturbations.[4] Whilst the magnitude of this complexation induced shift is often correlated with binding strength, it has been demonstrated that this is not always the case.[5] In the case of halogen bonding receptors where the principal interaction responsible for anion binding is not directly relayed by $^{1}H$ NMR, it is often observed that proton environments proximal to the anion binding site also experience downfield perturbations, which is presumably due to the formation of weak ancillary hydrogen bonding-anion interactions or merely via a through space effect of an anion being bound in proximity (Figure S39. Situation 1). However, it is also conceivable that anion binding to a halogen bonding receptor could induce other effects on the chemical shift of protons proximal to the binding site. Namely, it is possible that, the receptor could adopt a conformation in which HB and through space effects cannot operate, or in which no $^{1}H$ NMR shift maybe observed (Figure S39. Situation 2) or induce a conformational change that causes an upfield chemical shift (Figure S39. Situation 3), perhaps due to increased proximity of a neighbouring residue which acts to magnetically shield the proton environment being monitored.

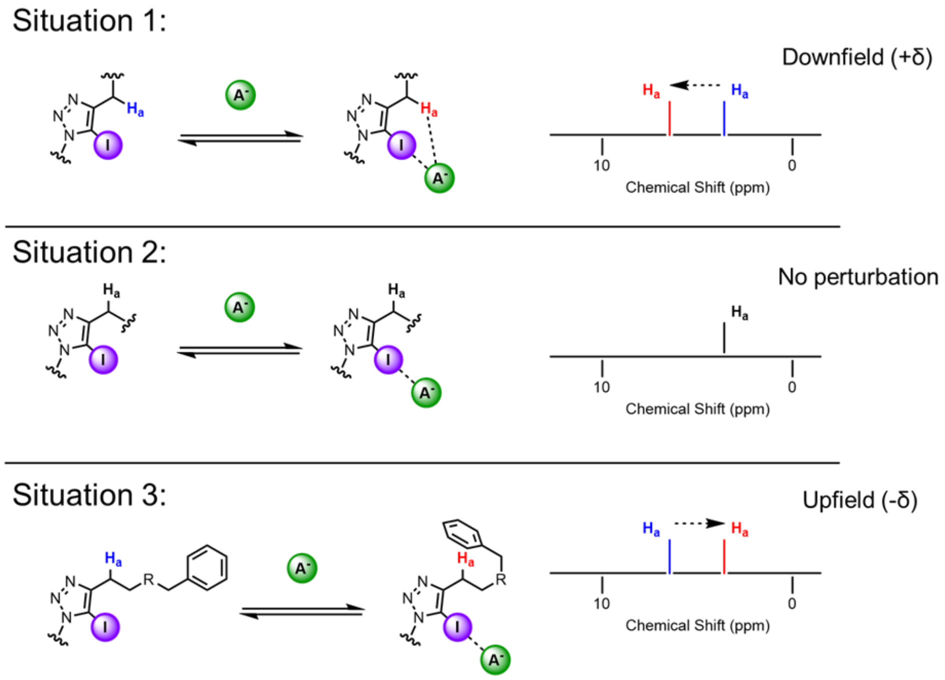


Figure S39. Schematic representation of the possible chemical shift perturbations observed during $^{1}H$ NMR anion titrations of halogen bonding systems.

In the case of the halogen bonding axle **2·XB**, the addition of halide anion titrant in either $D_2O$:Acetone-$d_6$ (5:95, v/v) or $D_2O$:Acetone-$d_6$ (10:90, v/v) consistently induced a downfield shift of proton signal c, this is consistent with a scenario represented by Figure S39 Situation 1. In the case of halogen bonding rotaxane, **3·XB**, anion titration experiments with chloride and bromide in either $D_2O$:Acetone-$d_6$ (5:95, v/v) or $D_2O$:Acetone-$d_6$ (10:90, v/v) elicited upfield shifts of proton signal c, which does not suggest the lack of halogen bonding anion interactions, but implies a scenario similar to Figure S39 Situation 3, where endotopic anion binding favours a conformation in which the macrocycle is held over the axle *via* hydrogen bonding anion interactions with the anion, thereby increasing any potential ring current effects originating from the hydroquinone groups of the macrocycle, thereby inducing a magnetic shielding effect. When similar experiments are conducted with iodide, a downfield shift of signal **c** is observed, whilst this still implies the participation of the halogen bond donor motif the contrasting direction of chemical shift perturbation is strong evidence for **3·XB** adopting a markedly different conformation to that observed for the chloride and bromide complexes. In concert with the larger $K_a$ values for chloride and bromide for **3·XB** relative to **2·XB**, we believe this supports the postulated endotopic binding mode for chloride and bromide and an exotopic binding mode for iodide (Figure S40).

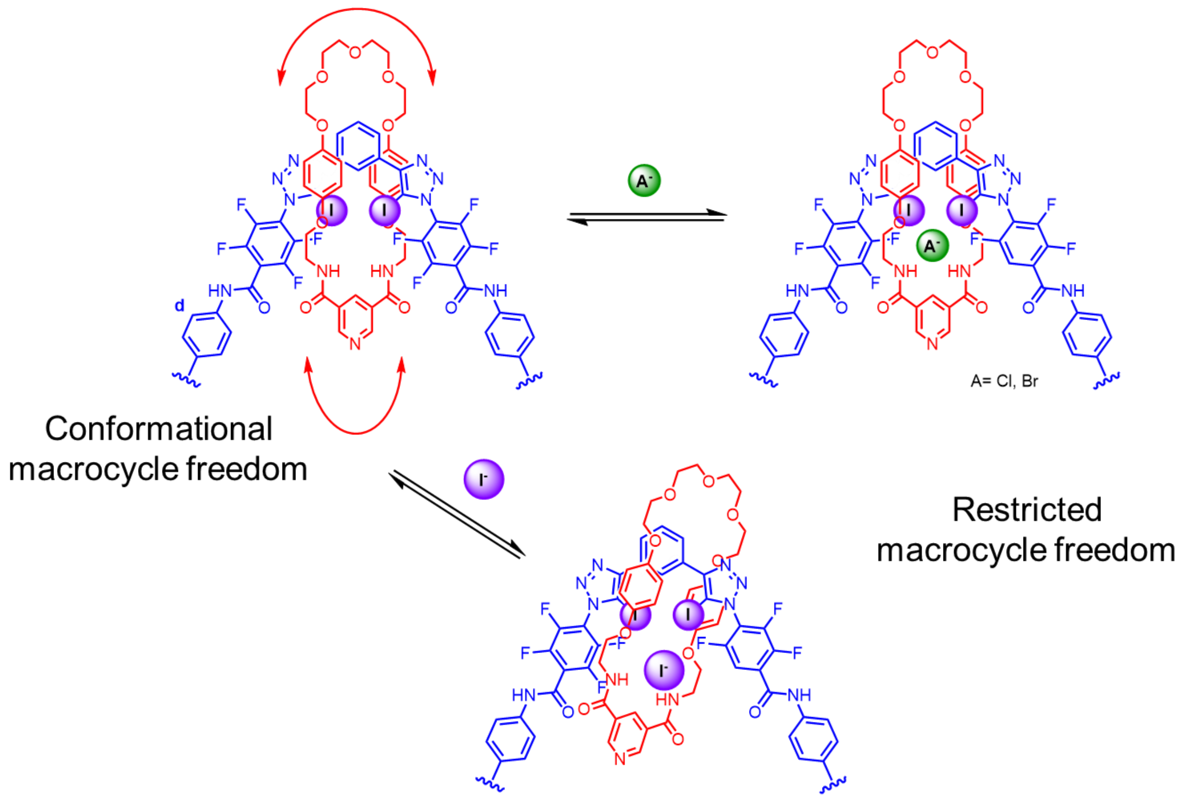


Figure S40. Schematic representation of the postulated halide anion binding conformations of **3·XB**.

In the case of **3·XB$^{(NO2)2}$** this situation of endoptopic binding for chloride or bromide and exoptopic binding for iodide is also observed. Although the anion induced chemical shift perturbations are not reversed for the two binding modes as is the case for **3·XB**, it is clear from inspection of the titration spectra that the chemical shift perturbations observed for chloride or bromide addition are considerably different in terms of magnitude and perturbation profile than those observed for iodide binding. Of particular note is the magnitude of chemical shift changes associated with anion addition e.g. 10 equivalents of $Cl^-$ to **3·XB$^{(NO2)2}$** in $D_2O$/Acetone-$d_6$ (5:95), induces a > 1 ppm shift in proton signal a, whilst the corresponding iodide addition induces a much less significant < 0.2 ppm perturbation in the same signal.

## Discussion regarding the use of proton signals for the purpose binding constant determination

In general, the selection of a proton signal to track for the purposes of binding constant determination is on the basis of the following criteria; the magnitude of the perturbation should be sufficient as to minimise error and the effect of non-specific changes resulting from dielectric constant changes, etc. As such proton resonances closest to the binding site are often chosen. However, for complex spectra the signals which may be most suitable by the aforementioned criteria may be unsuitable for other reasons, for example their coalescence with other signals may limit the number of useable data points in this series. When this is the case the selection of different signals may be employed to determine binding constants, which can be compared relative to each other, but given the multitude of other effects that can also induce chemical shift perturbations it is important to establish that these different signals give consistent binding constant values. In this study for the reasons mentioned above, in the instances when different protons signals have been used it was confirmed that the tracking of multiple signals gave rise to concordant values. Detailed below are the instances when this method has been applied.

In general, the anion induced chemical shift perturbation of axle component proton signal **c** was used for binding constant determination. However, in the case of the TBABr titration of **3·XB** in $D_2O$/Acetone-d$_6$ (10:90), the magnitude of perturbation of **c** was too small to accurately determine a $K_a$ value, as such proton signal **2** was used which determined a $K_a(Br^-)$ = 460 $M^{-1}$. To confirm the reliability of using an alternative signal, proton signal **2** was also used to determine chloride and iodide affinity, which gave $K_a$ values concordant with those obtained using proton signal **c**, summarised in Table S1.

**Table S1**: Anion association constants ($K_a$ / $M^{-1}$) for **2·XB** and **3·XB**.[a]

| | $D_2O$/Acetone-d$_6$ (5:95) | | $D_2O$/Acetone-d$_6$ (10:90) | | |
|---|---|---|---|---|---|
| Anion[b] | **2·XB** | **3·XB** | **2·XB** | **3·XB** | **3·XB**[d] |
| $Cl^-$ | 415 | 3330 | -[c] | 274 | 222 |
| $Br^-$ | 1320 | 6580 | 236 | -[e] | 460 |
| $I^-$ | 2190 | 1550 | 760 | 551 | 550 |

[a] $K_a$ values calculated using Bindfit software using a 1:1 host-guest binding model, fitted from perturbations in proton signal **c** unless specified, errors < 10%. [b] Anions added as their tetrabutylammonium salts. [c] No binding. [d] Determined from fitting of proton signal **2**. [e] Chemical shift perturbations too small to accurately determine $K_a$.

For the dinitro derivatives the external proton signal **a** was used for the purposes of fitting, as this signal was consistently trackable in both **2·XB$^{(NO2)2}$** and **3·XB$^{(NO2)2}$**. To confirm the validity of using signal **a** instead of **b**, for the titration of **2·XB$^{(NO2)2}$** with chloride, bromide and iodide in $D_2O$/Acetone-$d_6$ (5:95), both signals **a** and **b** were used to determine $K_a$ which gave concordant values.

In a similar fashion, attempting to fit a 1:1 host-guest stoichiometric binding model to the perturbations observed for proton signal **a** for **3·XB$^{(NO2)2}$** titrated with iodide in either (5:95) or (10:90) $D_2O$/Acetone-$d_6$ gave poor quality fits, again presumably due to anion-induced macrocycle co-conformational changes also eliciting chemical shift changes. As such the macrocycle proton signals **4/4'** were used in this case, as the perturbations in these signals are presumably attributable to a single cause – namely the co-conformational changes brought about by anion binding, and not a combination of multiple effects and determined $K_a(I^-) = 2660\ M^{-1}$. To confirm the validity of using alternative signals in data fitting, $K_a(Cl^-)$ and $K_a(Br^-)$ were also determined using this method and are also shown in Table S2, which gave values concordant with those obtained from fitting proton signal **a**.

**Table S2**: Anion association constants ($K_a$ / $M^{-1}$) for **2·XB$^{(NO2)2}$** and **3·XB$^{(NO2)2}$**.[a]

| | $D_2O$/Acetone-$d_6$ (5:95) | | | $D_2O$/Acetone-$d_6$ (10:90) | | | |
|---|---|---|---|---|---|---|---|
| Anion[b] | **2·XB$^{(NO2)2}$** [c] | **2·XB$^{(NO2)2}$** | **3·XB$^{(NO2)2}$** | **2·XB$^{(NO2)2}$** | **2·XB$^{(NO2)2}$** [c] | **3·XB$^{(NO2)2}$** | **3·XB$^{(NO2)2}$** [e] |
| $Cl^-$ | 1630 | 1690 | > $10^5$ | 206 | 155 | 3800 | 3740 |
| $Br^-$ | 5890 | 5900 | > $10^5$ | 955 | 914 | 5760 | 6450 |
| $I^-$ | 12600 | 14000 | -[d] | 3050 | 3000 | -[d] | 2660 |

[a] $K_a$ values calculated using Bindfit software using a 1:1 host-guest binding model, fitted from perturbations in proton signal **a** unless specified, errors < 10%. [b] Anions added as their tetrabutylammonium salts. [c] Fitted from proton signal **b**. [d] Non-reliably fittable data. [e] Determined from fitting of proton signal **4/4'**.

Summarised anion binding isotherms and stacked $^{1}$H NMR spectra

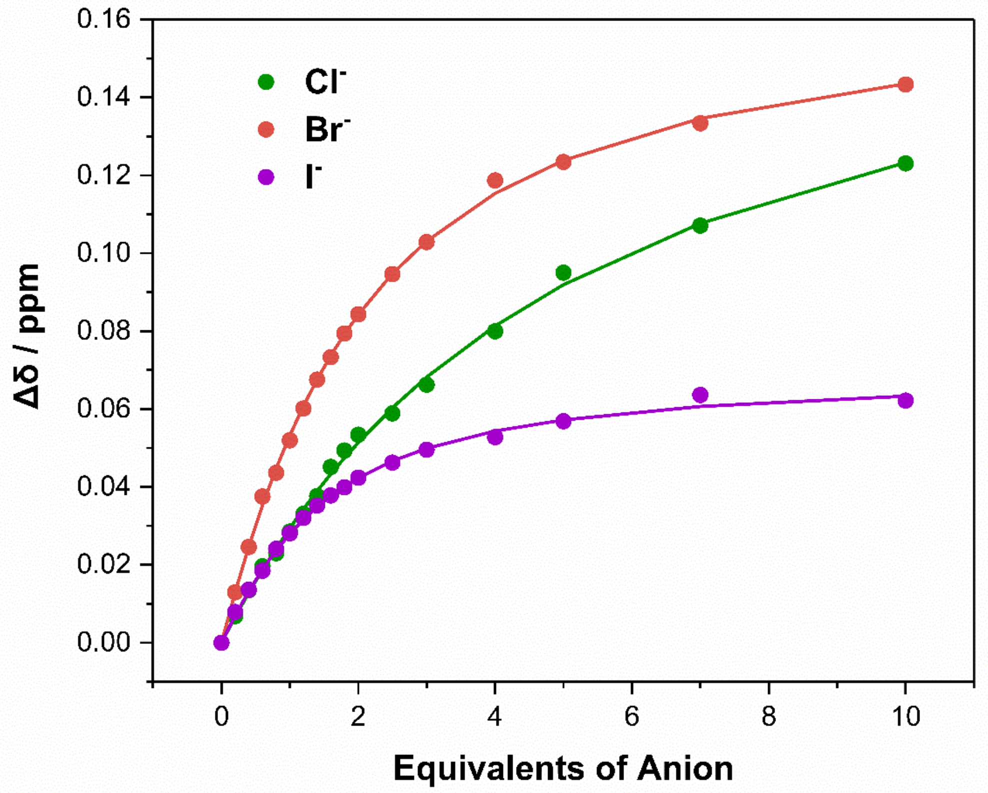


Figure S41. Anion binding isotherm for **2·XB** in $D_2O$:Acetone-$d_6$ (5:95, v/v), monitoring proton signal **c**, where circles represent experimental data and the lines represent the fitted isotherm.

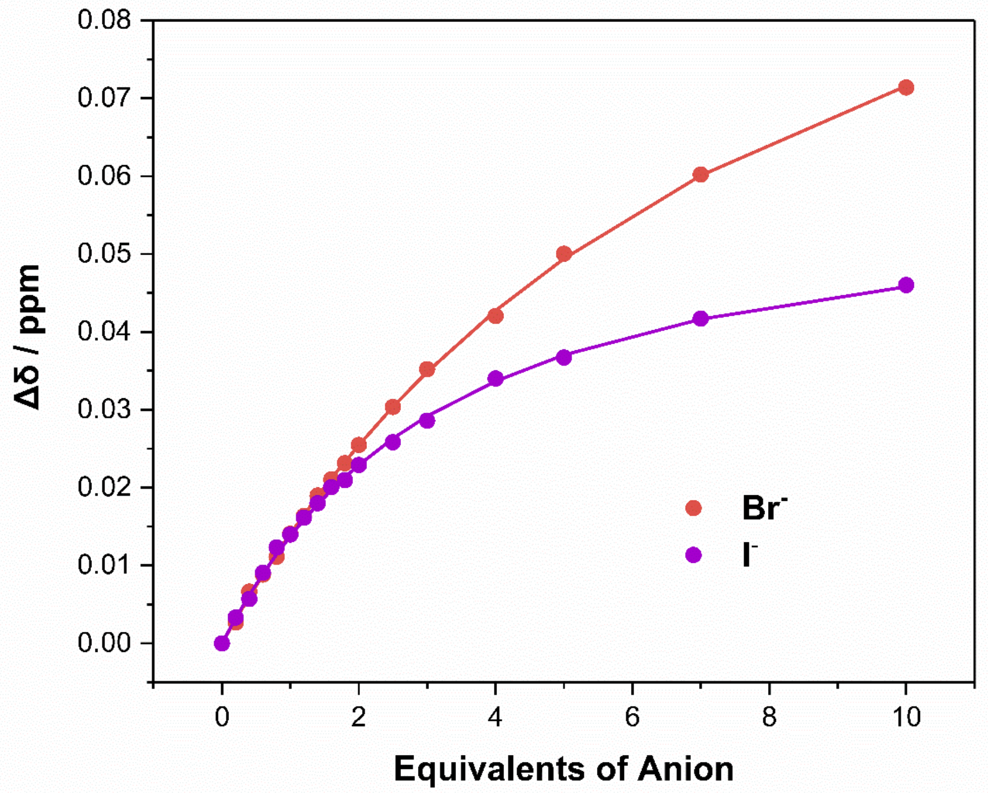


Figure S42. Anion binding isotherm for **2·XB** in $D_2O$:Acetone-$d_6$ (10:90, v/v), monitoring proton signal **c**, where circles represent experimental data and the lines represent the fitted isotherm.

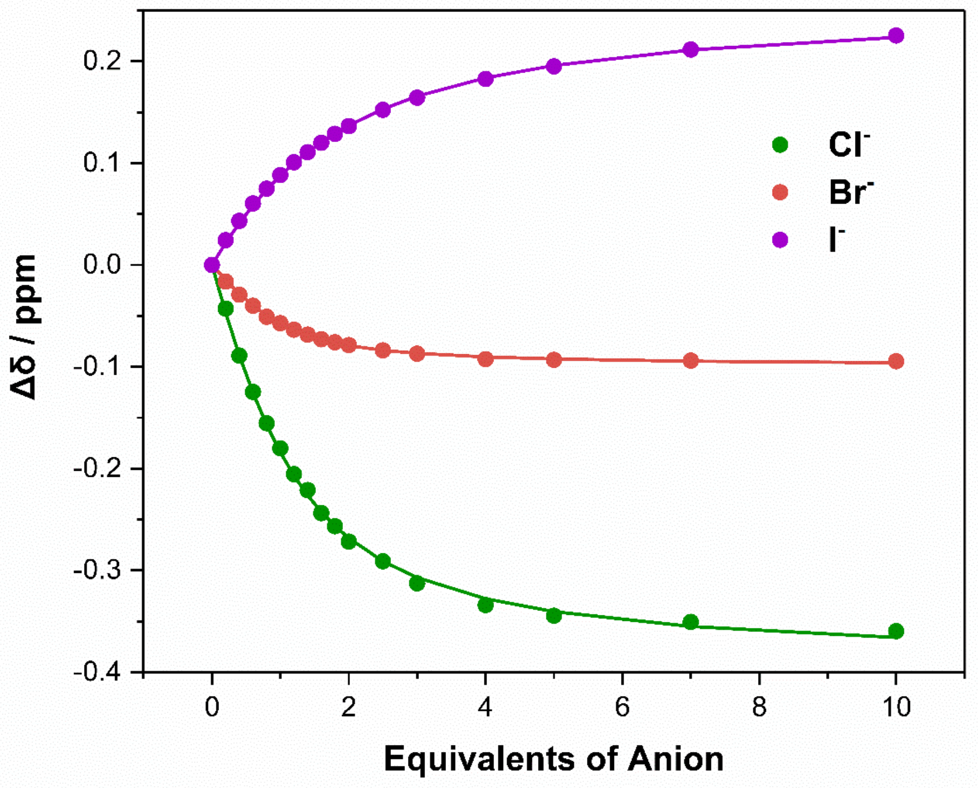


Figure S43. Anion binding isotherm for **3·XB** in $D_2O$:Acetone-$d_6$ (5:95, v/v), monitoring proton signal **c**, where circles represent experimental data and the lines represent the fitted isotherm.

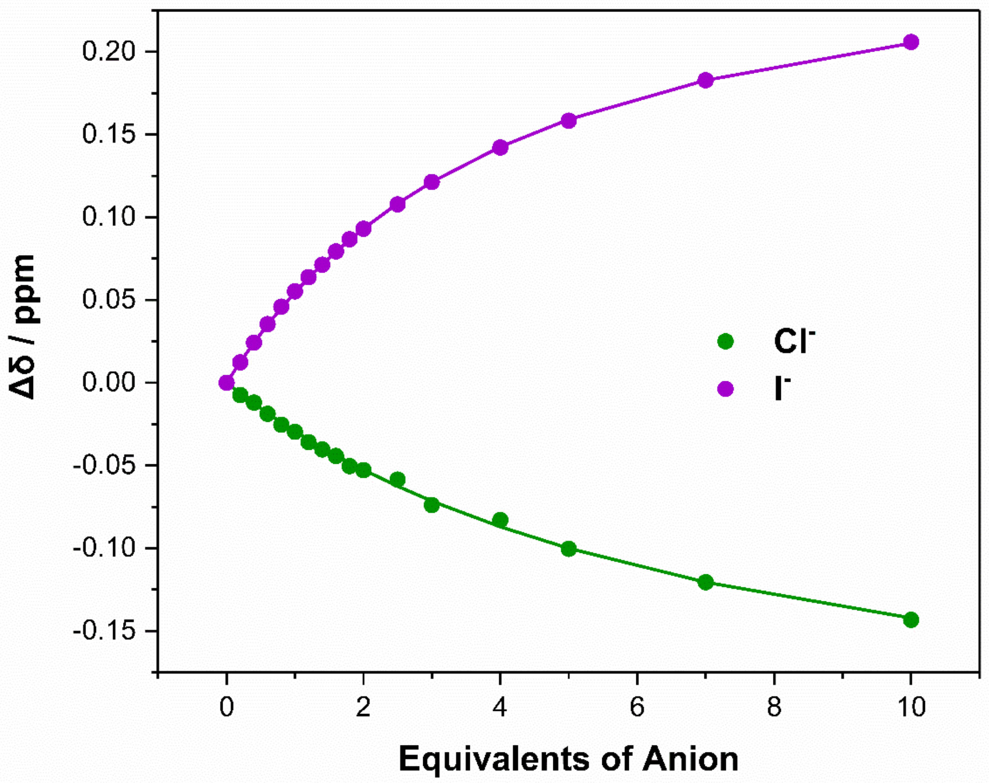


Figure S44. Anion binding isotherm for **3·XB** in $D_2O$:Acetone-$d_6$ (10:90, v/v), monitoring proton signal **c**, where circles represent experimental data and the lines represent the fitted isotherm.

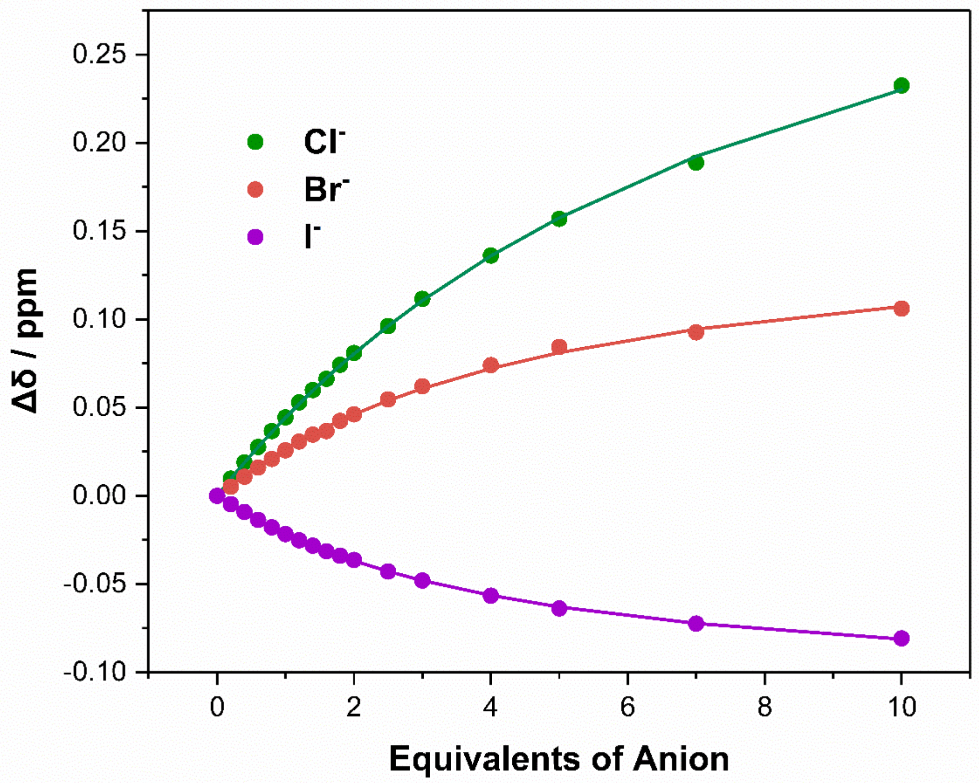


Figure S45. Anion binding isotherm for **3·XB** in $D_2O$:Acetone-$d_6$ (10:90, v/v), monitoring proton signal **2**, where circles represent experimental data and the lines represent the fitted isotherm.

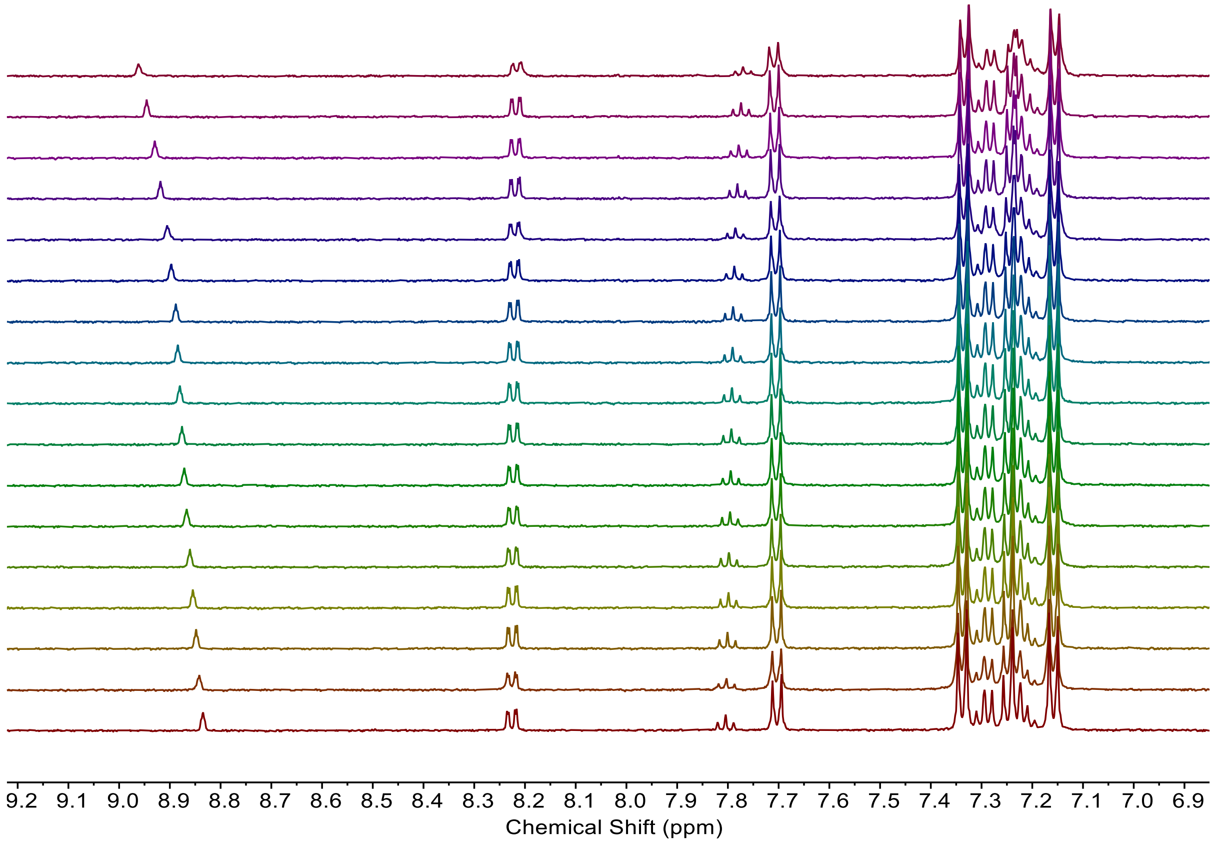


Figure S46. Stacked $^{1}H$ NMR TBACl titration of **2·XB** in $D_2O$:Acetone-$d_6$ (5:95, v/v), 500 MHz, 298 K.

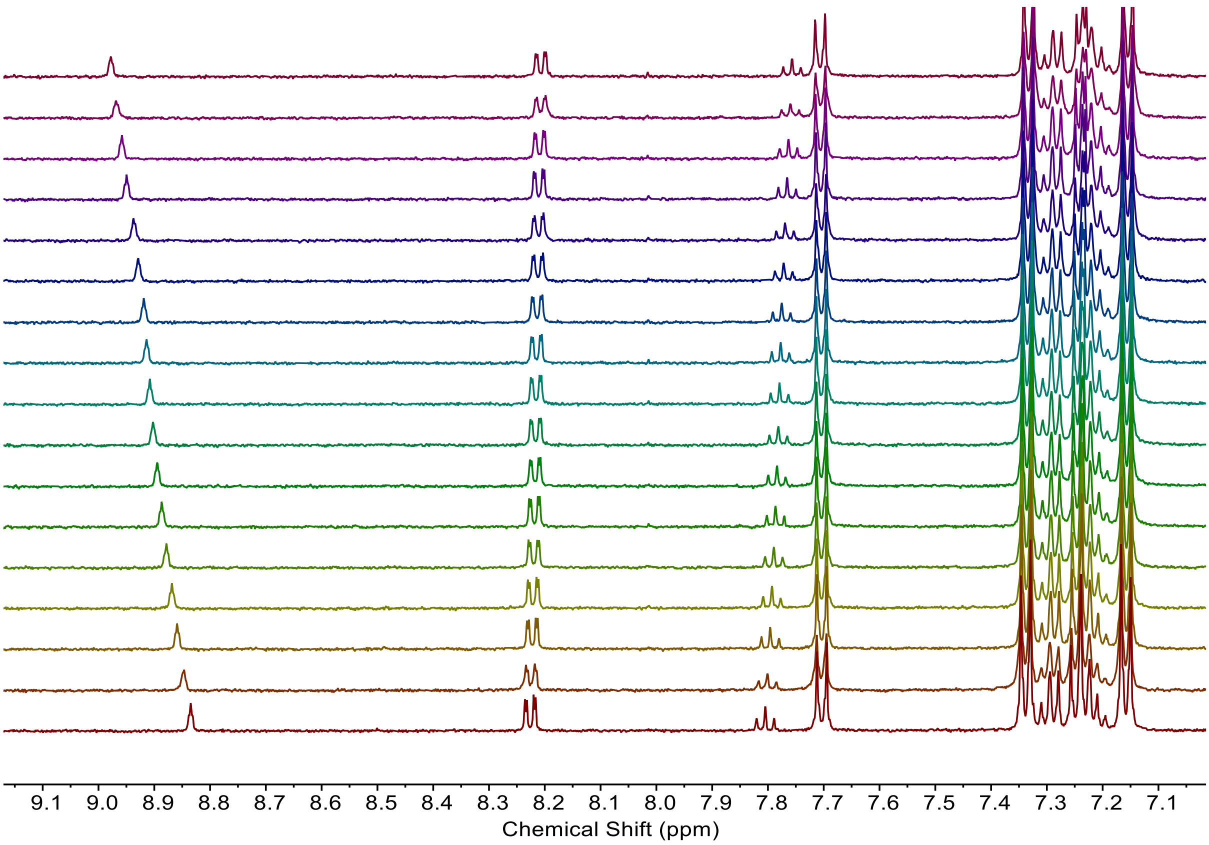


Figure S47. Stacked $^{1}$H NMR TBABr titration of **2·XB** in $D_2O$:Acetone-$d_6$ (5:95, v/v), 500 MHz, 298 K.

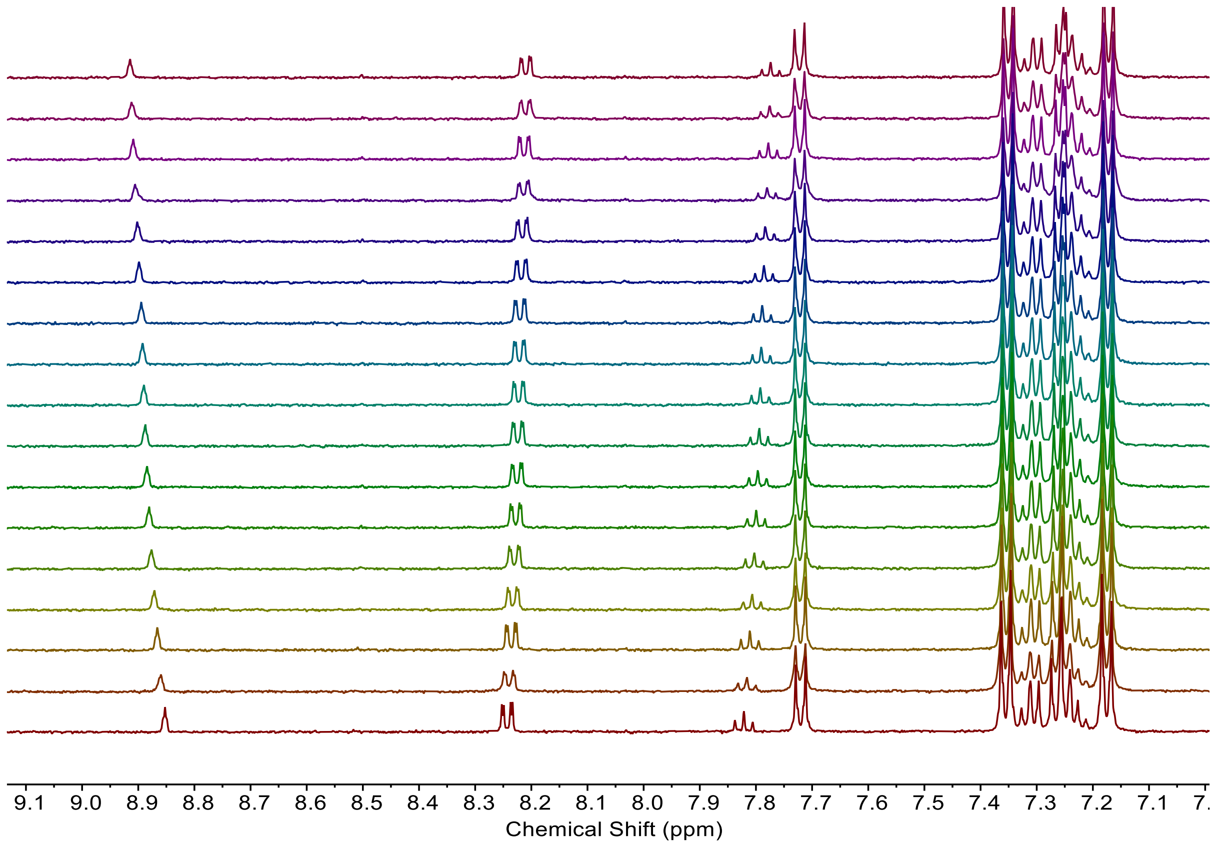


Figure S48. Stacked $^{1}$H NMR TBAI titration of **2·XB** in $D_2O$:Acetone-$d_6$ (5:95, v/v), 500 MHz, 298 K.

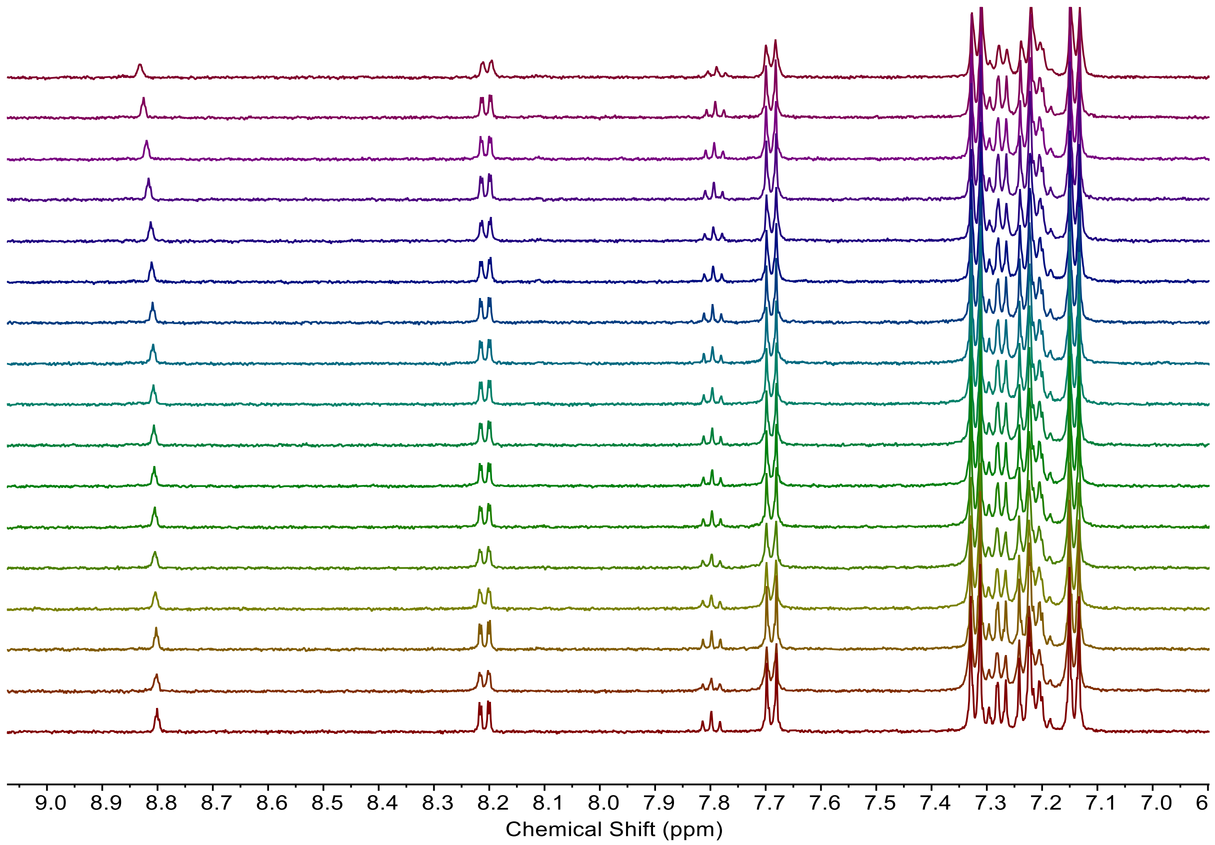


Figure S49. Stacked $^1$H NMR TBACl titration of **2·XB** in $D_2O$:Acetone-$d_6$ (10:90, v/v), 500 MHz, 298 K.

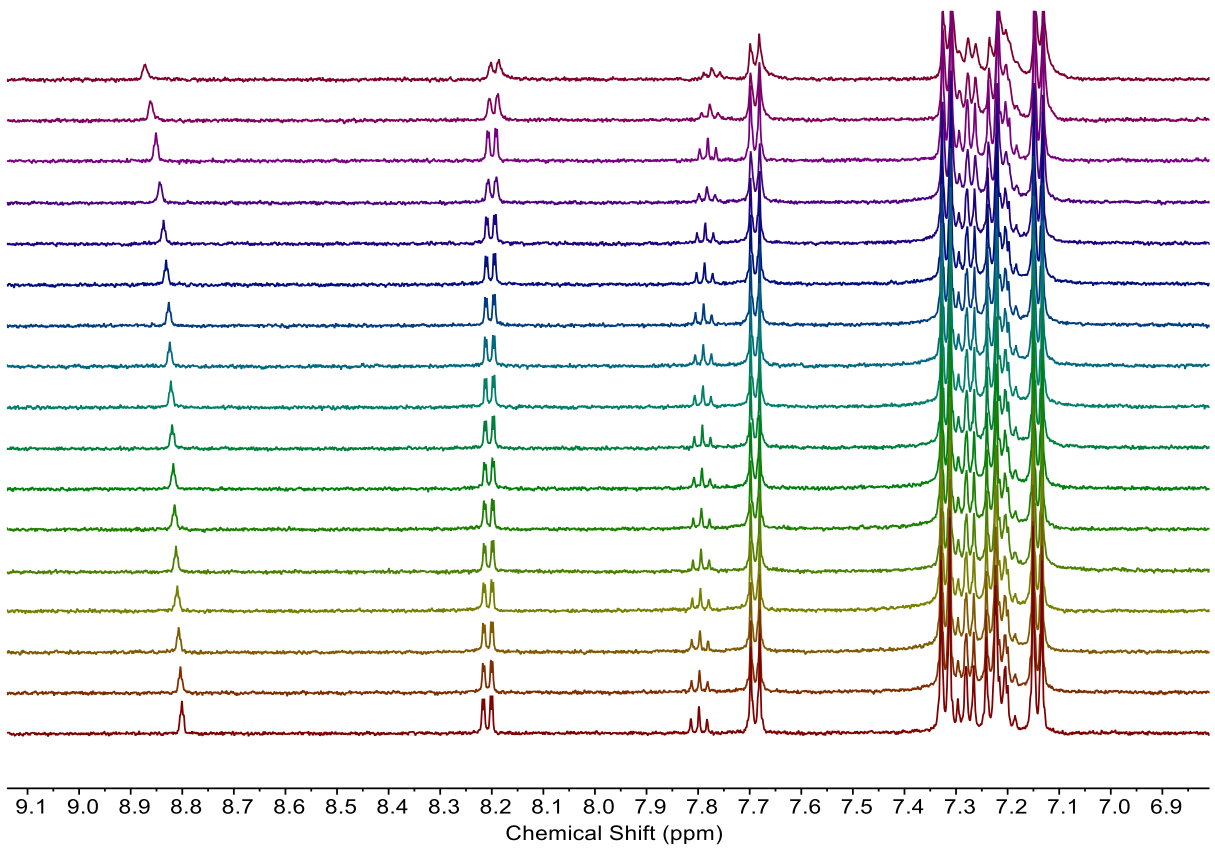


Figure S50. Stacked $^{1}H$ NMR TBABr titration of **2·XB** in $D_2O$:Acetone-$d_6$ (10:90, v/v), 500 MHz, 298 K.

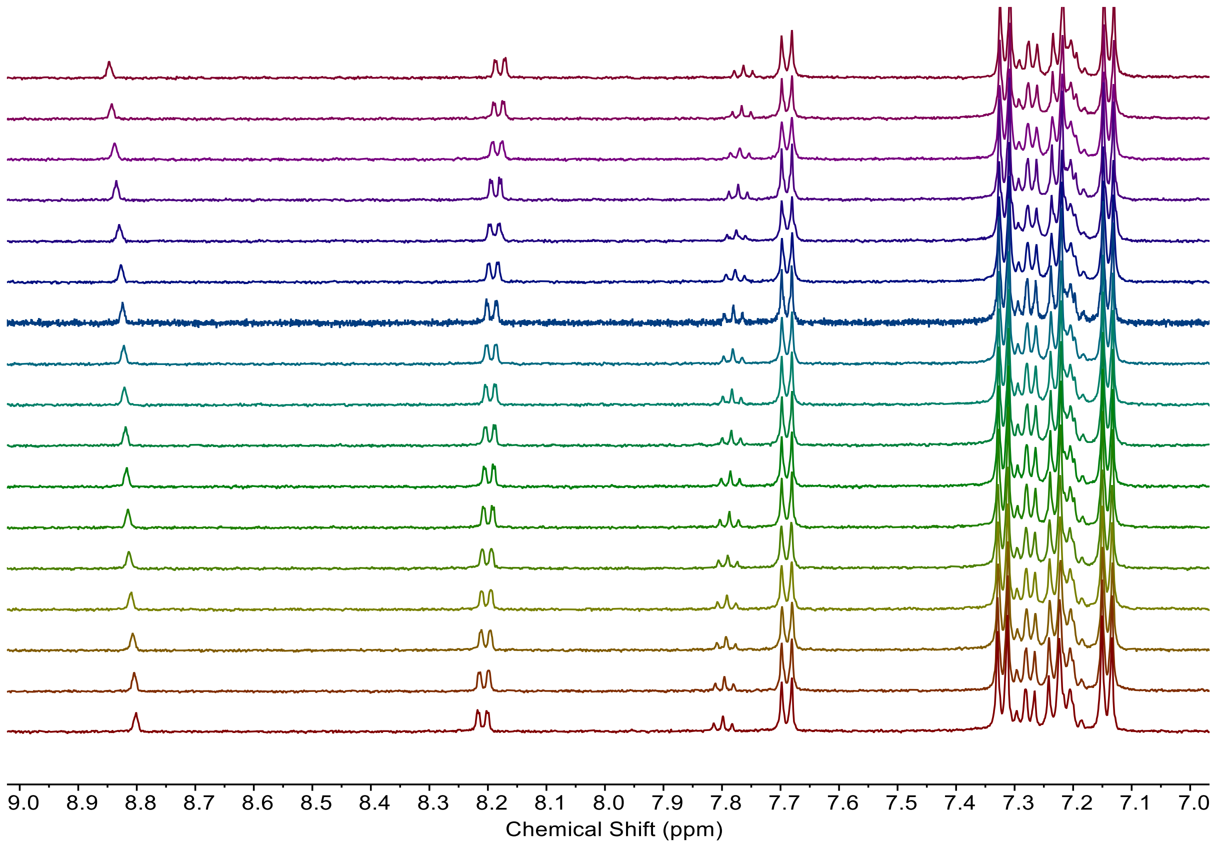


Figure S51. Stacked $^{1}$H NMR TBAI titration of **2·XB** in $D_2O$:Acetone-$d_6$ (10:90, v/v), 500 MHz, 298 K.

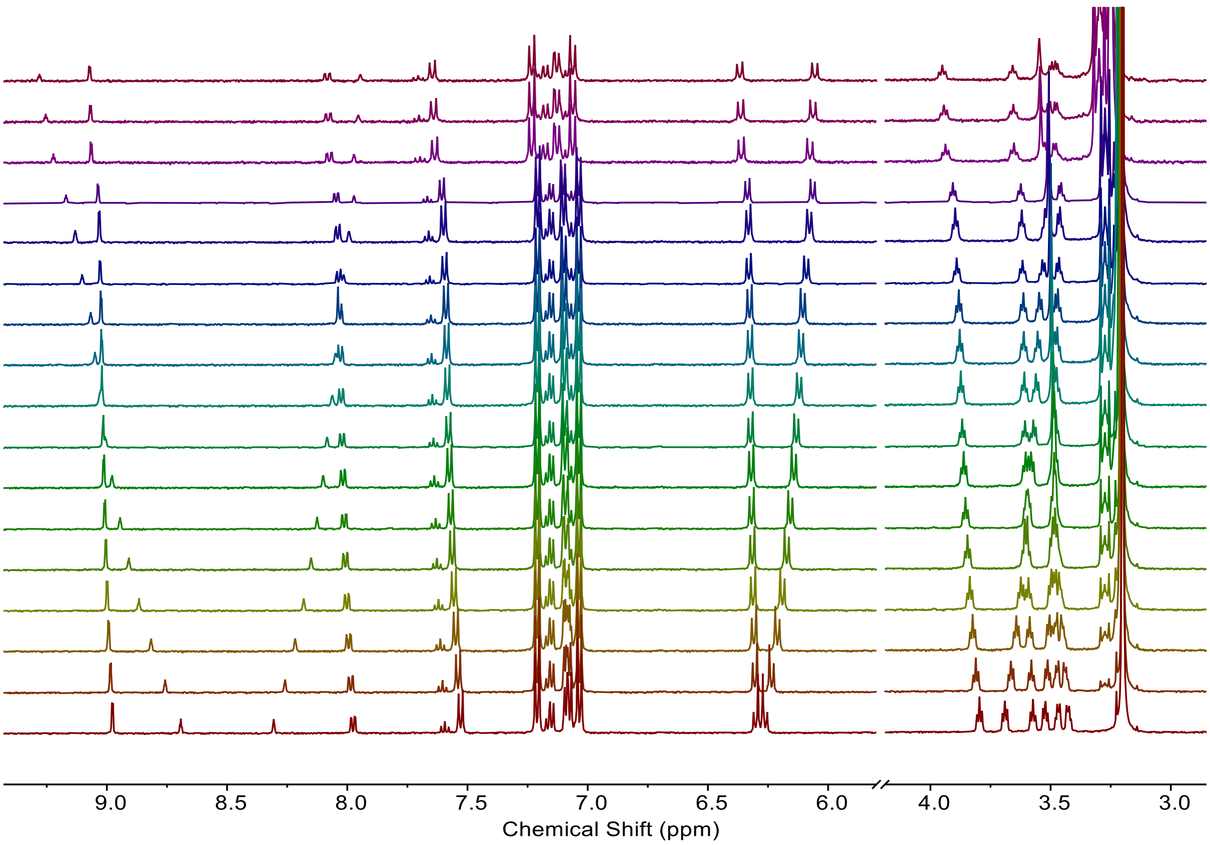


Figure S52. Stacked $^{1}$H NMR TBACl titration of **3·XB** in $D_2O$:Acetone-$d_6$ (5:95, v/v), 500 MHz, 298 K.

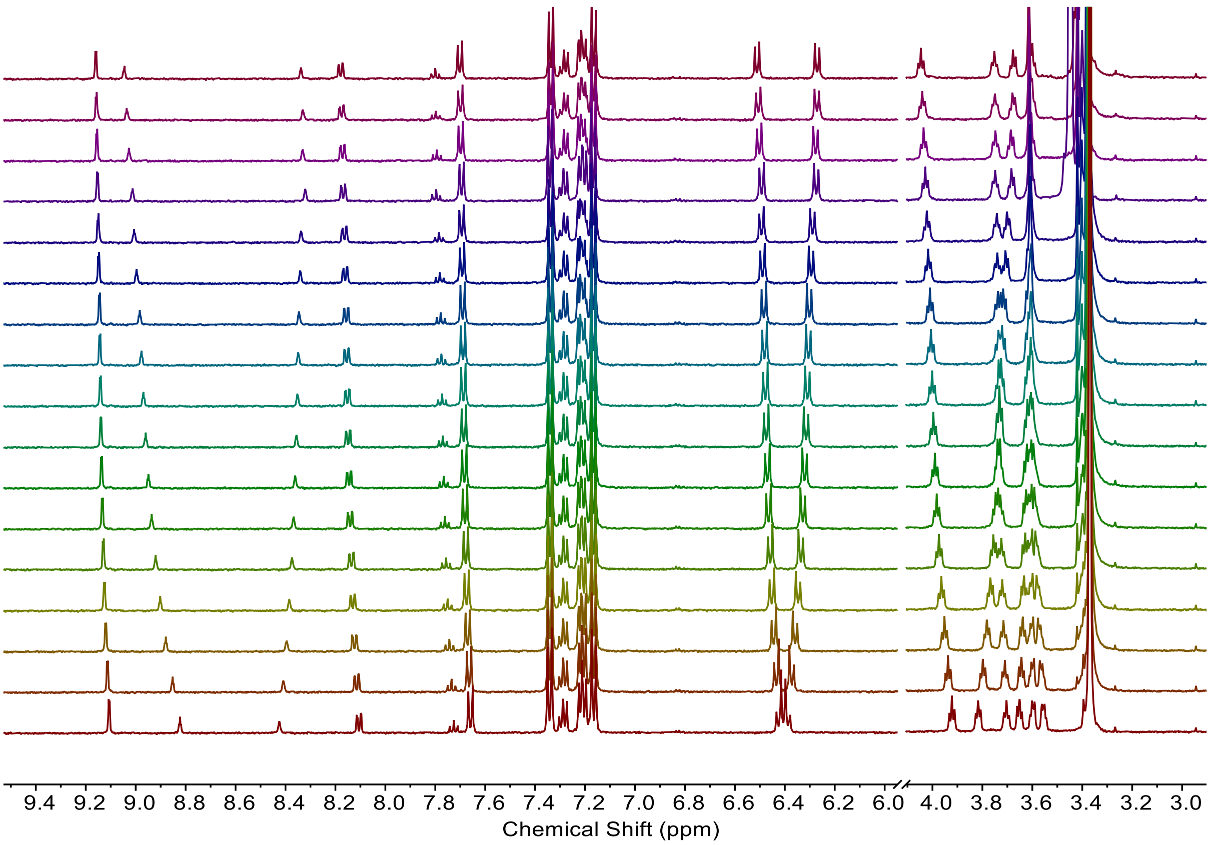


Figure S52. Stacked $^{1}H$ NMR TBABr titration of **3·XB** in $D_2O$:Acetone-$d_6$ (5:95, v/v), 500 MHz, 298 K.

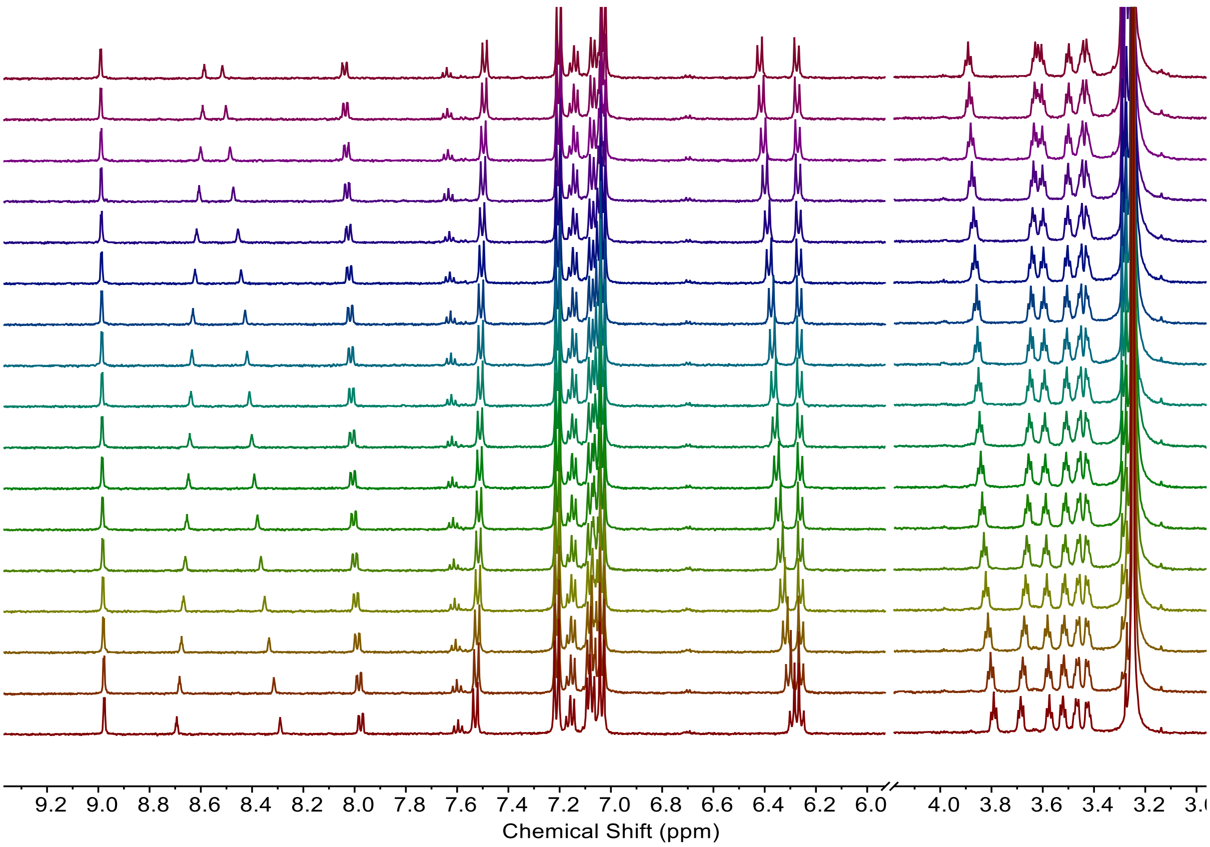


Figure S53. Stacked $^{1}H$ NMR TBAI titration of **3·XB** in $D_2O$:Acetone-$d_6$ (5:95, v/v), 500 MHz, 298 K.

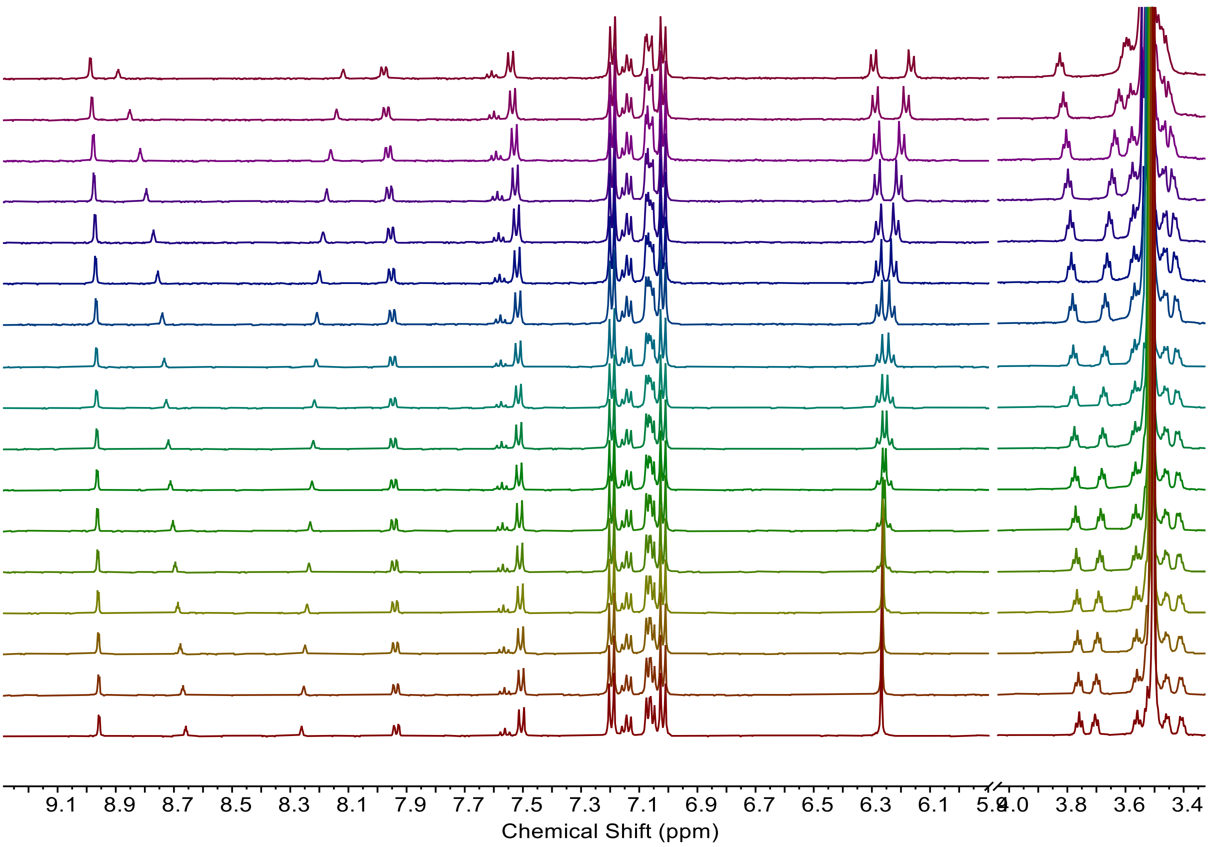


Figure S54. Stacked $^{1}$H NMR TBACl titration of **3·XB** in $D_2O$:Acetone-$d_6$ (10:90, v/v), 500 MHz, 298 K.

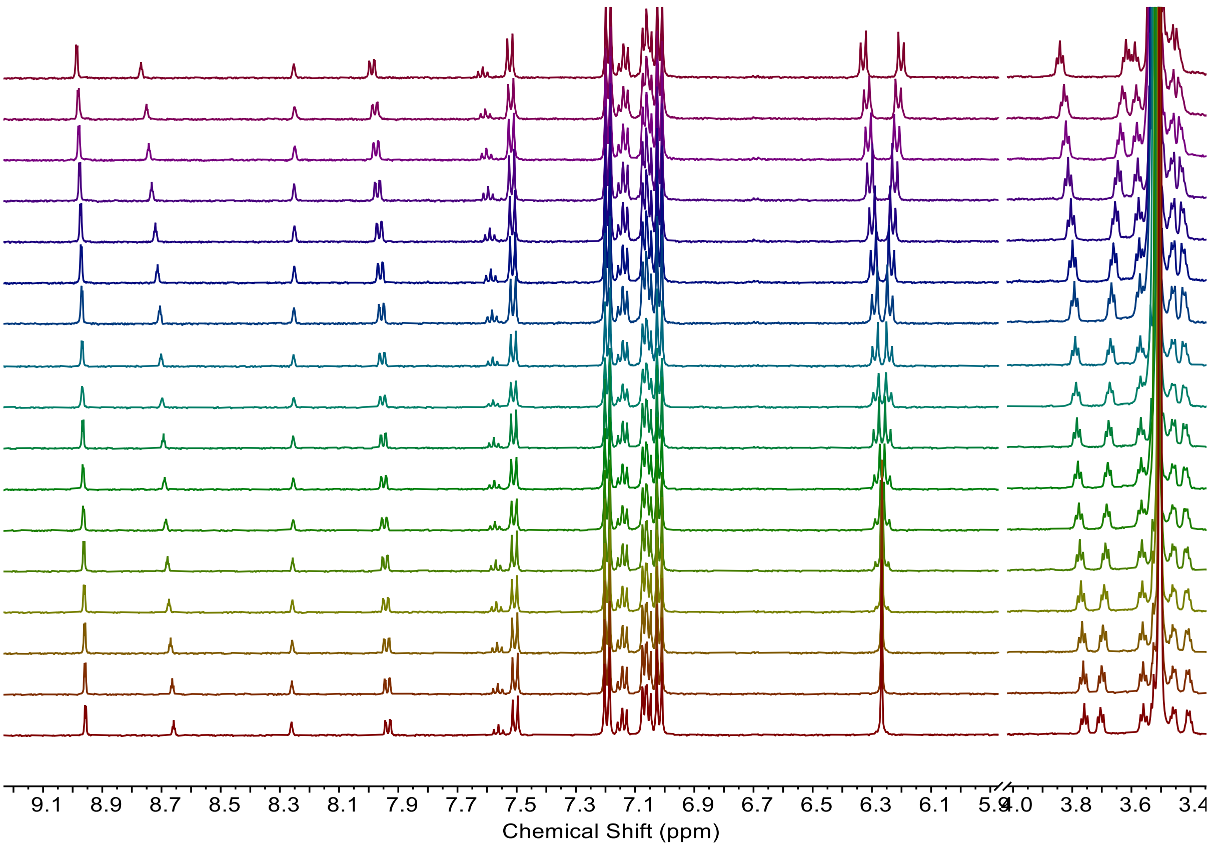


Figure S55. Stacked $^{1}H$ NMR TBABr titration of **3·XB** in $D_2O$:Acetone-$d_6$ (10:90, v/v), 500 MHz, 298 K.

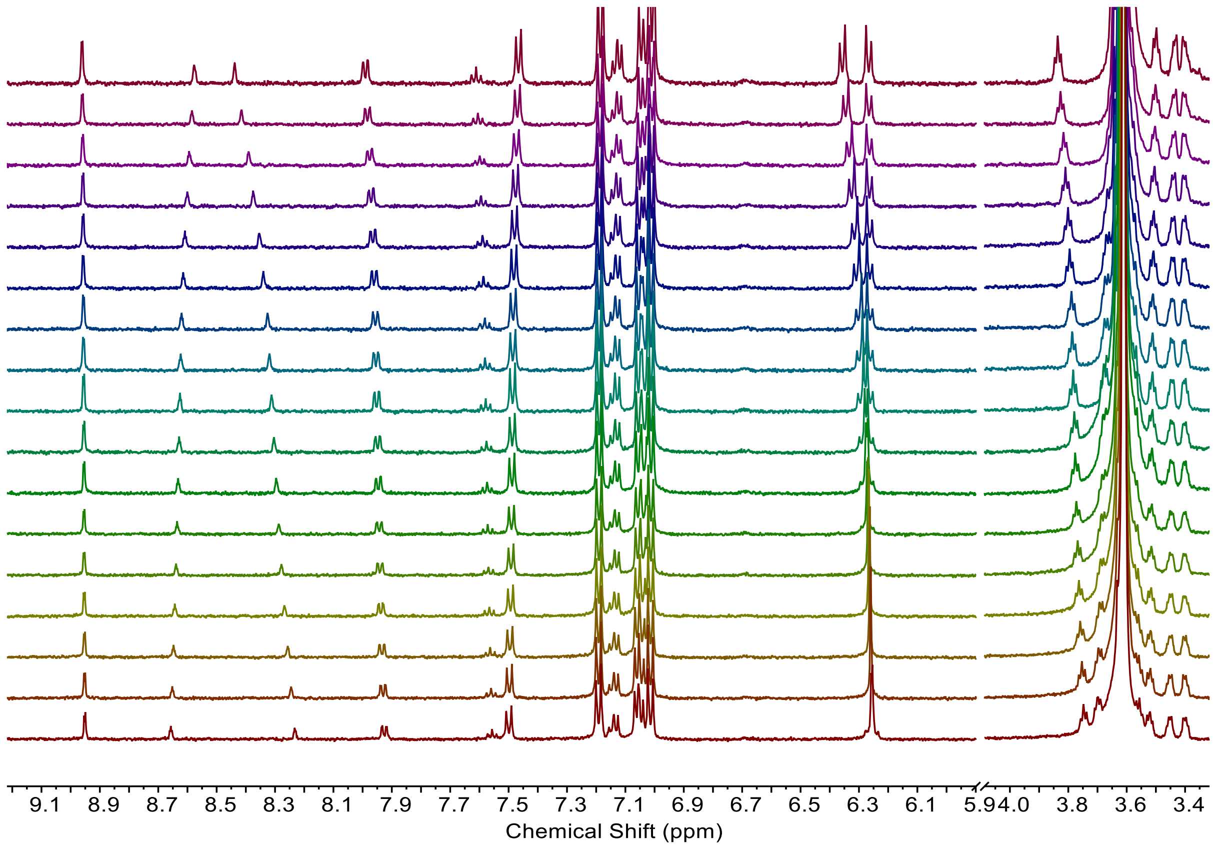


Figure S56. Stacked $^{1}$H NMR TBAI titration of **3·XB** in $D_2O$:Acetone-$d_6$ (10:90, v/v), 500 MHz, 298 K.

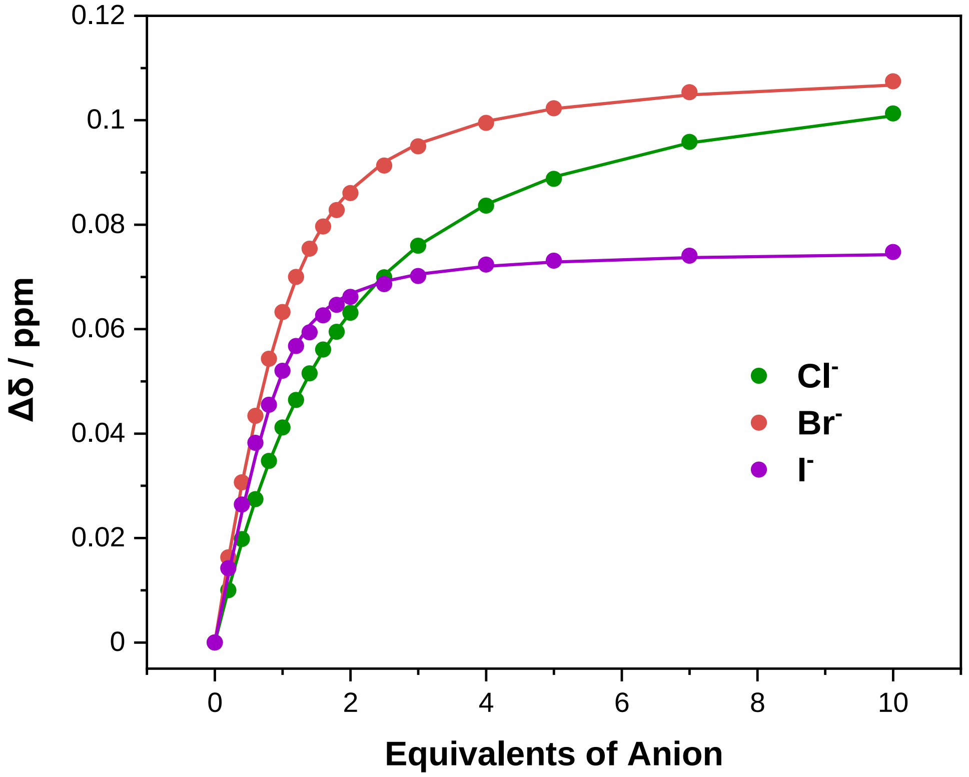


Figure S57. Anion binding isotherms for **2·XB$^{(NO2)2}$** in $D_2O$:Acetone-$d_6$ (5:95, v/v), monitoring proton signal **b**, where circles represent experimental data and the lines represent the fitted isotherm.

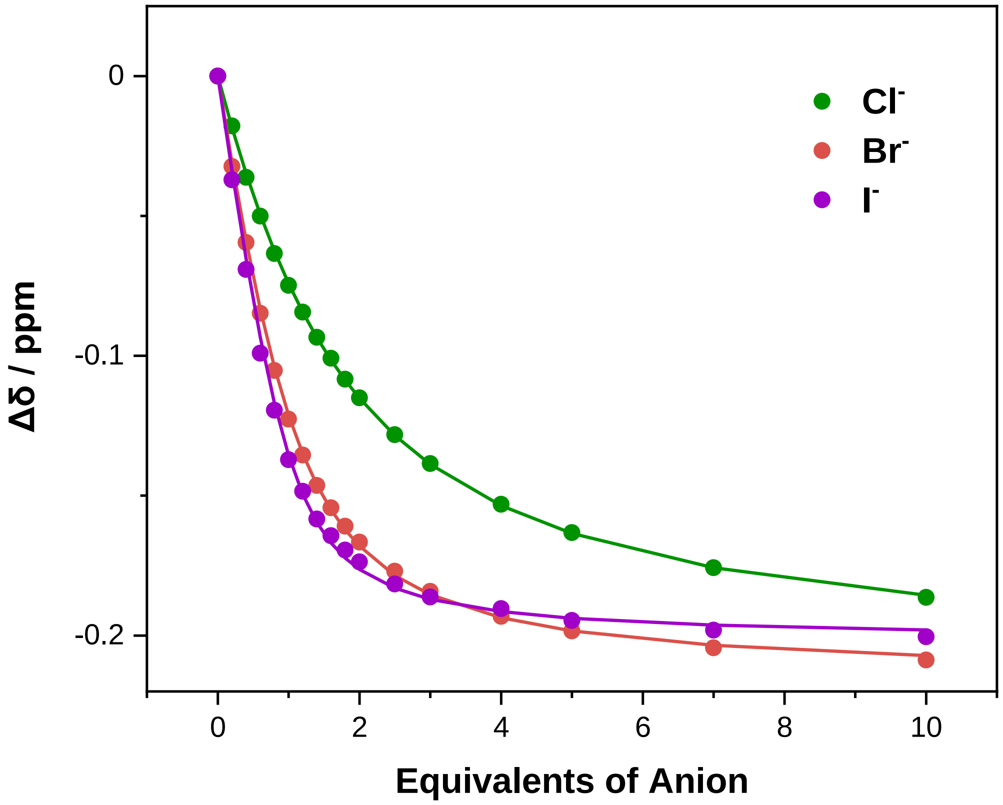


Figure S58. Anion binding isotherms for **2·XB$^{(NO2)2}$** in $D_2O$:Acetone-$d_6$ (5:95, v/v), monitoring proton signal **a**, where circles represent experimental data and the lines represent the fitted isotherm.

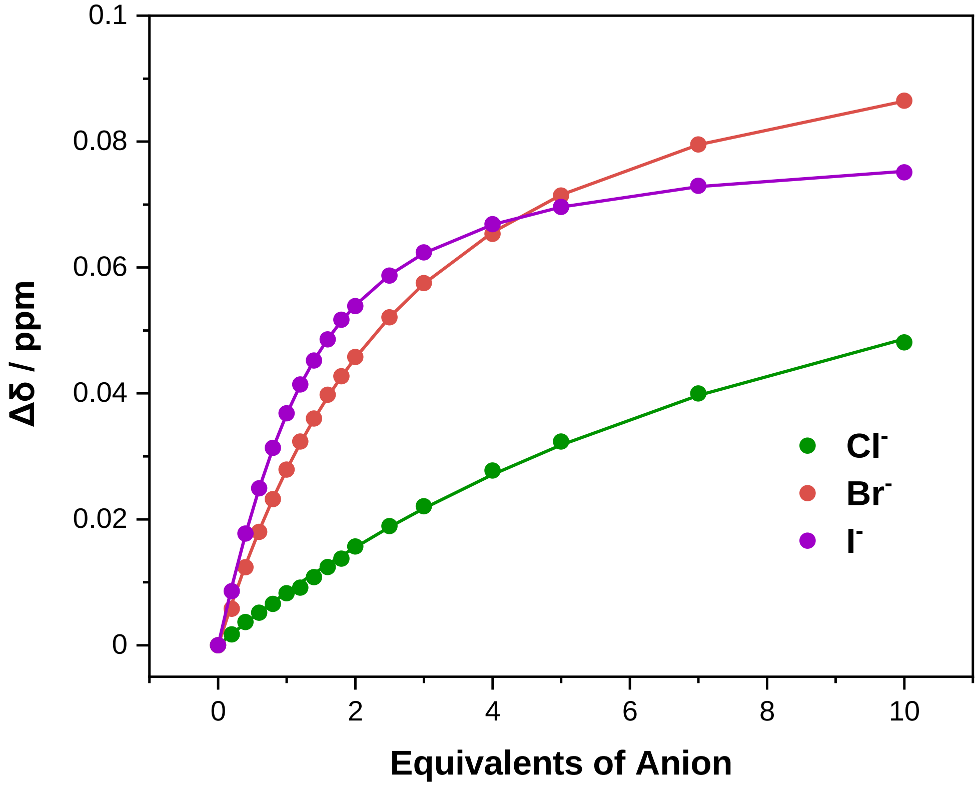


Figure S59. Anion binding isotherms for **2·XB$^{(NO2)2}$** in $D_2O$:Acetone-$d_6$ (10:90, v/v), monitoring proton signal **b**, where circles represent experimental data and the lines represent the fitted isotherm.

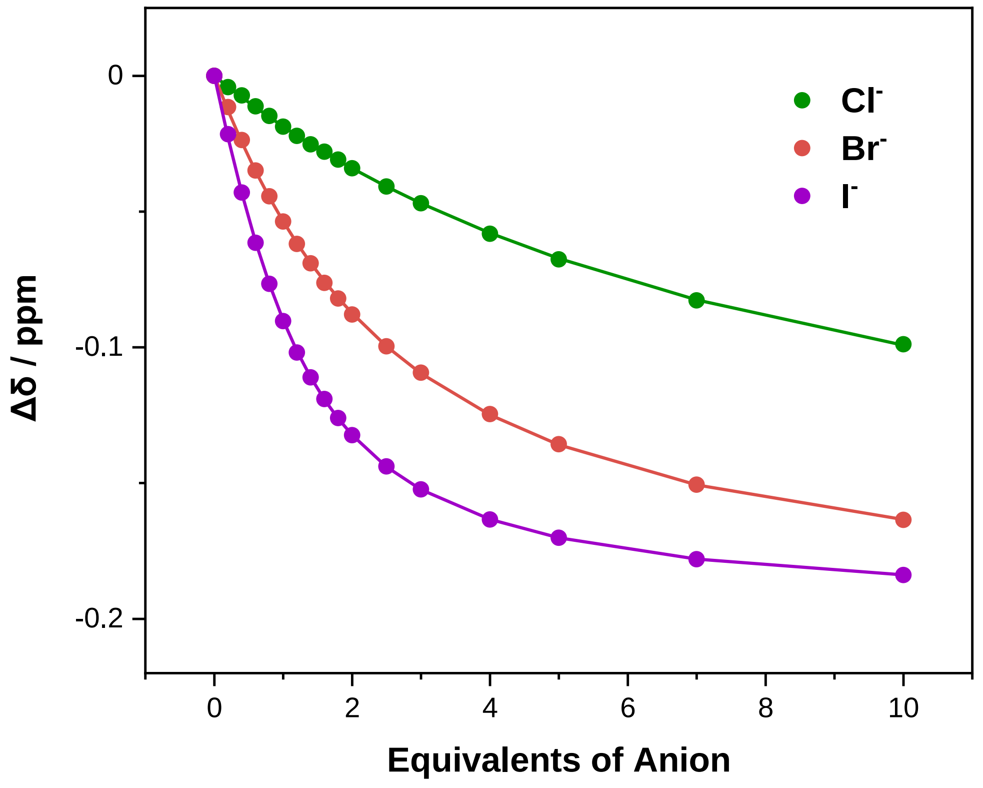


Figure S60. Anion binding isotherms for **2·XB$^{(NO2)2}$** in $D_2O$:Acetone-$d_6$ (10:90, v/v), monitoring proton signal **a**, where circles represent experimental data and the lines represent the fitted isotherm.

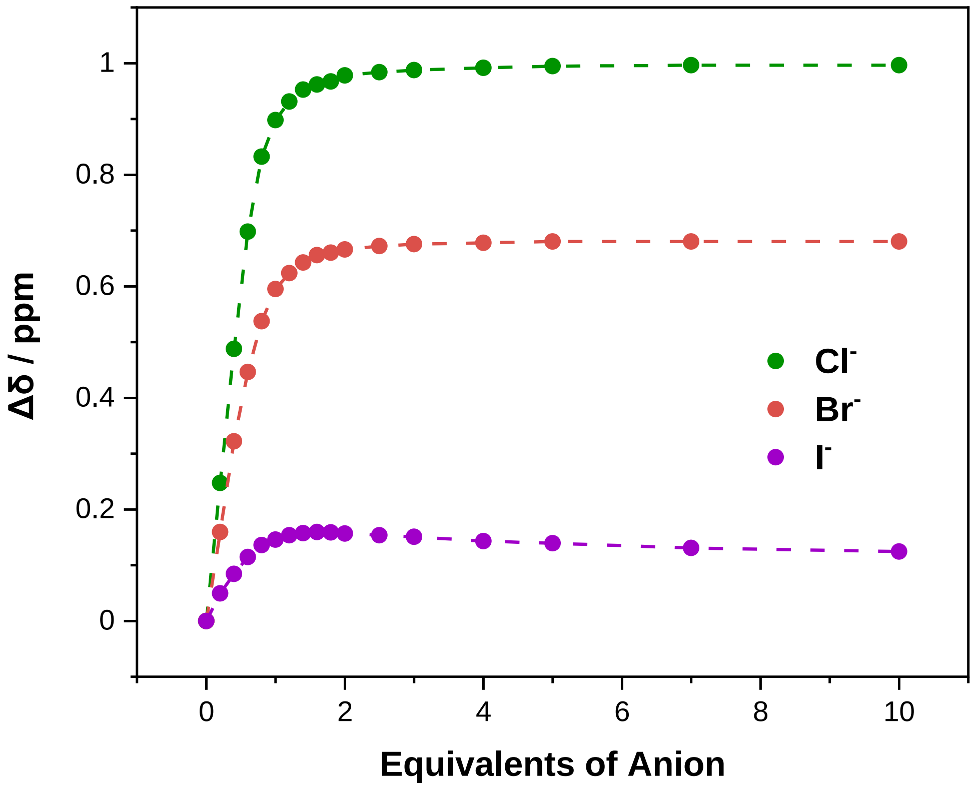


Figure S61. Anion binding isotherms for **3·XB^(NO2)2^** in $D_2O$:Acetone-$d_6$ (5:95, v/v), monitoring proton signal **a**, where circles represent experimental data and the dotted lines are a visual aid.

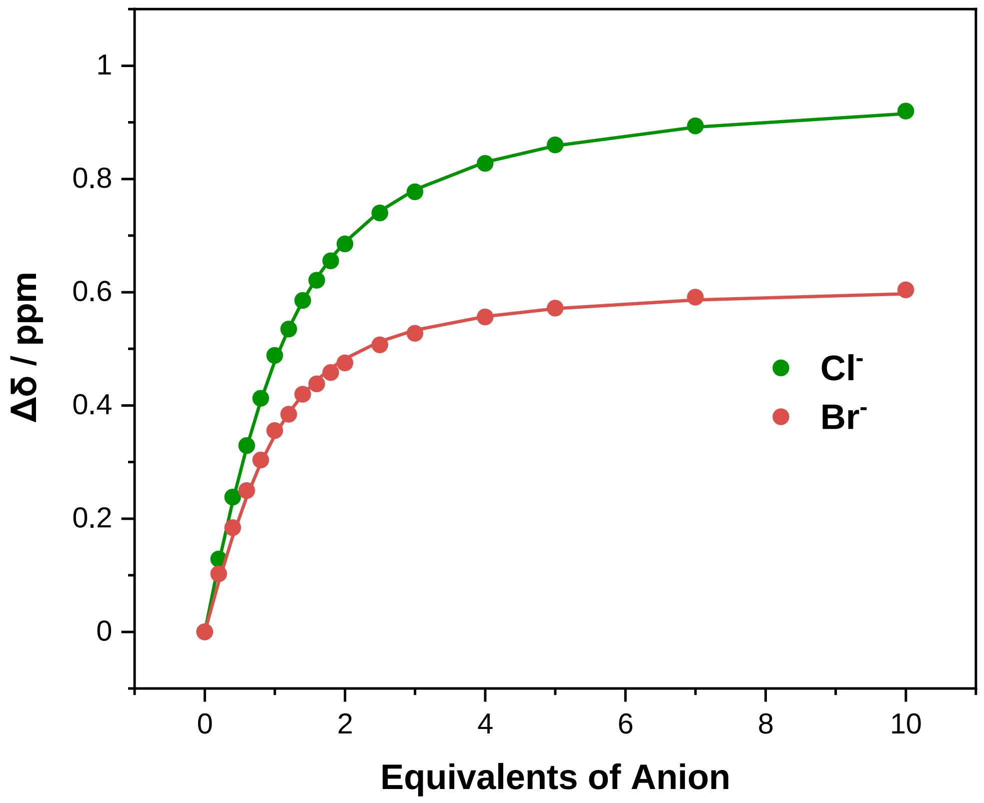


Figure S62. Anion binding isotherms for **3·XB$^{(NO2)2}$** in $D_2O$:Acetone-$d_6$ (10:90, v/v), monitoring proton signal **a**, where circles represent experimental data and the lines represent the fitted isotherm.

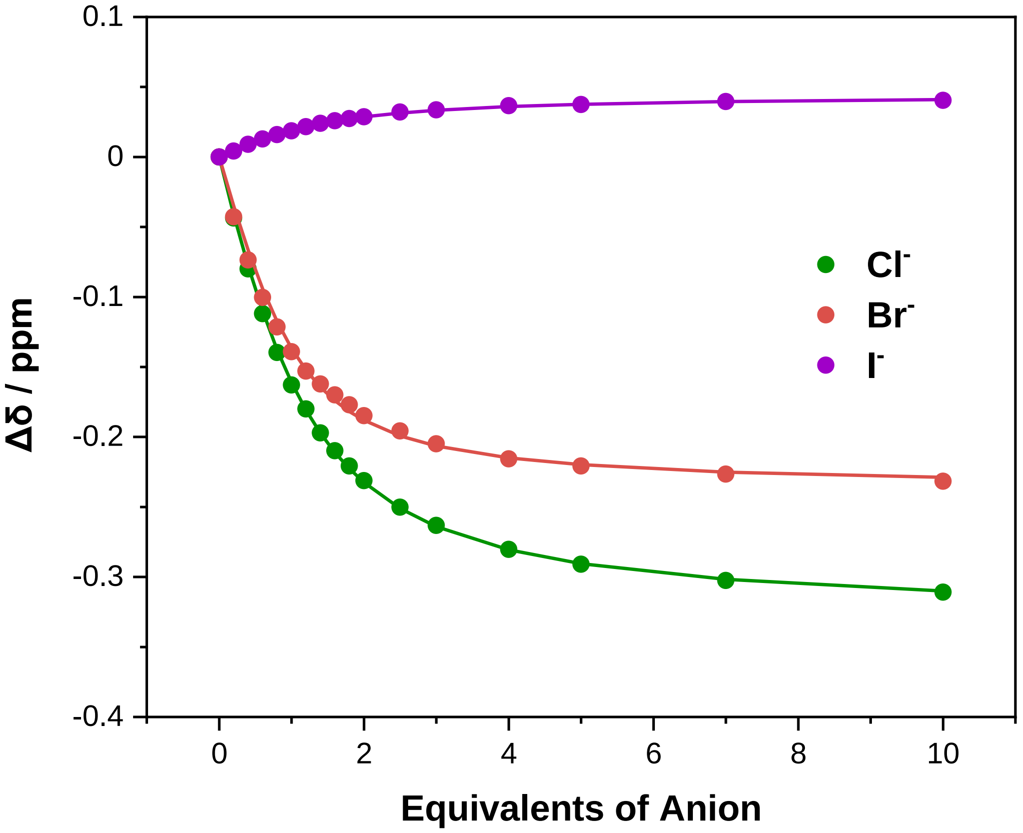


Figure S63. Anion binding isotherms for **3·XB$^{(NO2)2}$** in $D_2O$:Acetone-$d_6$ (10:90, v/v), monitoring proton signal **4/4'**, where circles represent experimental data and the lines represent the fitted isotherm.

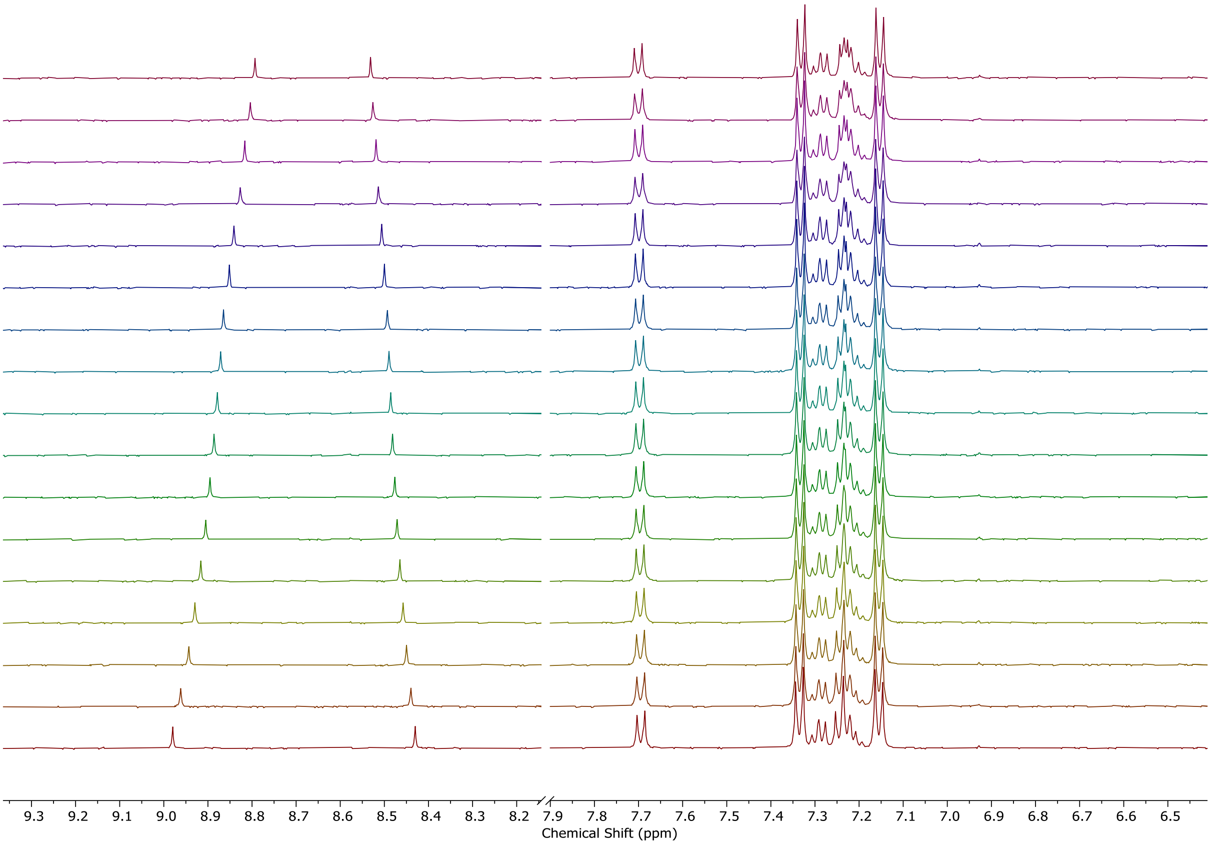


Figure S64. Stacked $^{1}$H NMR TBACl titration of **2·XB$^{(NO2)2}$** in $D_2O$:Acetone-$d_6$ (5:95, v/v), 500 MHz, 298 K.

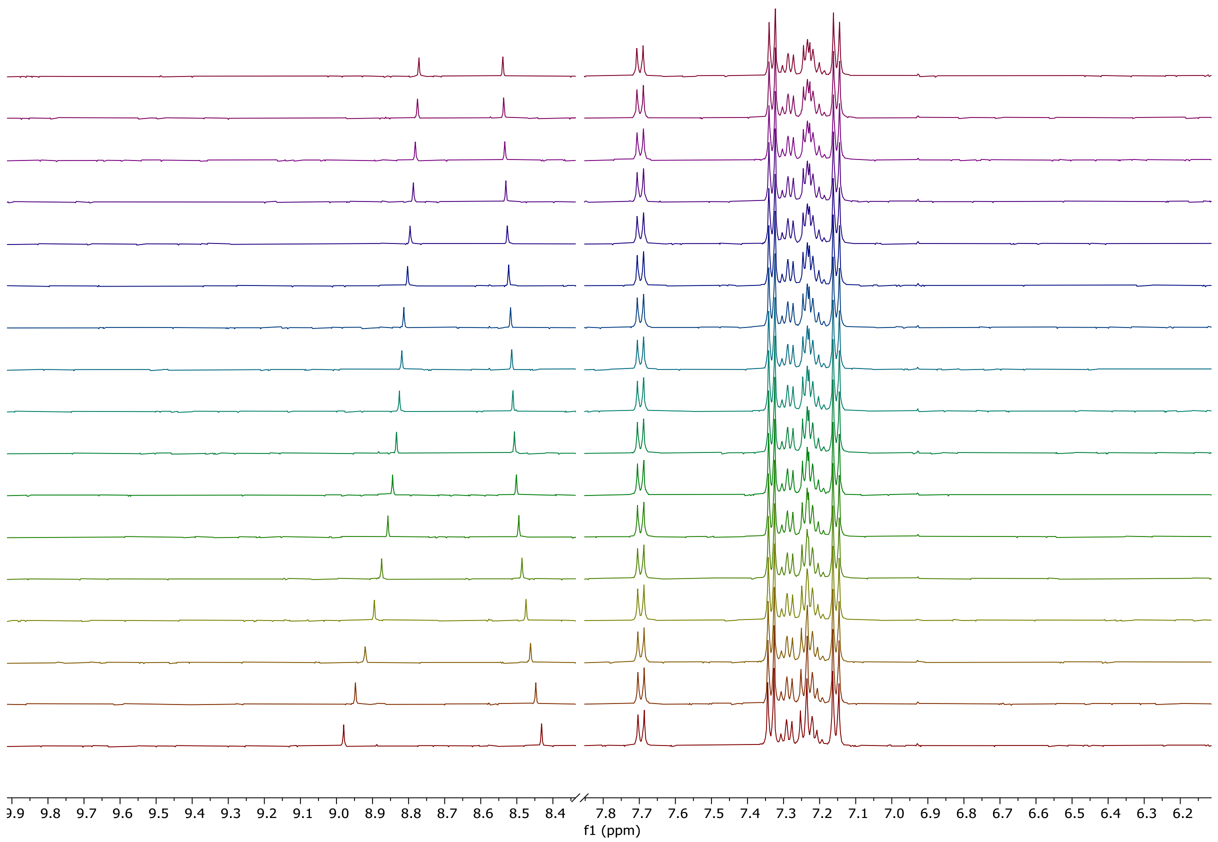


Figure S65. Stacked $^1$H NMR TBABr titration of **2·XB$^{(NO2)2}$** in $D_2O$:Acetone-$d_6$ (5:95, v/v), 500 MHz, 298 K.

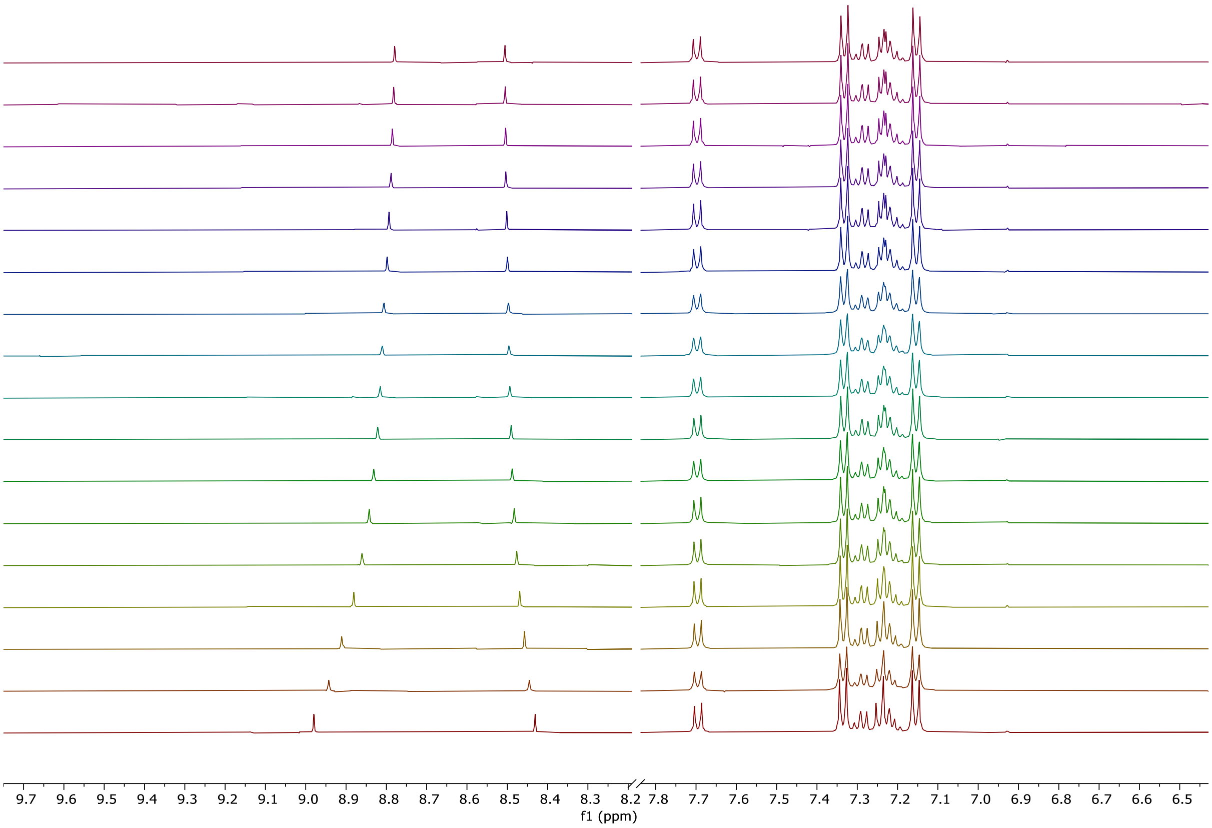


Figure S66. Stacked $^{1}$H NMR TBAI titration of **2·XB$^{\mathbf{(NO2)2}}$** in $D_2O$:Acetone-$d_6$ (5:95, v/v), 500 MHz, 298 K.

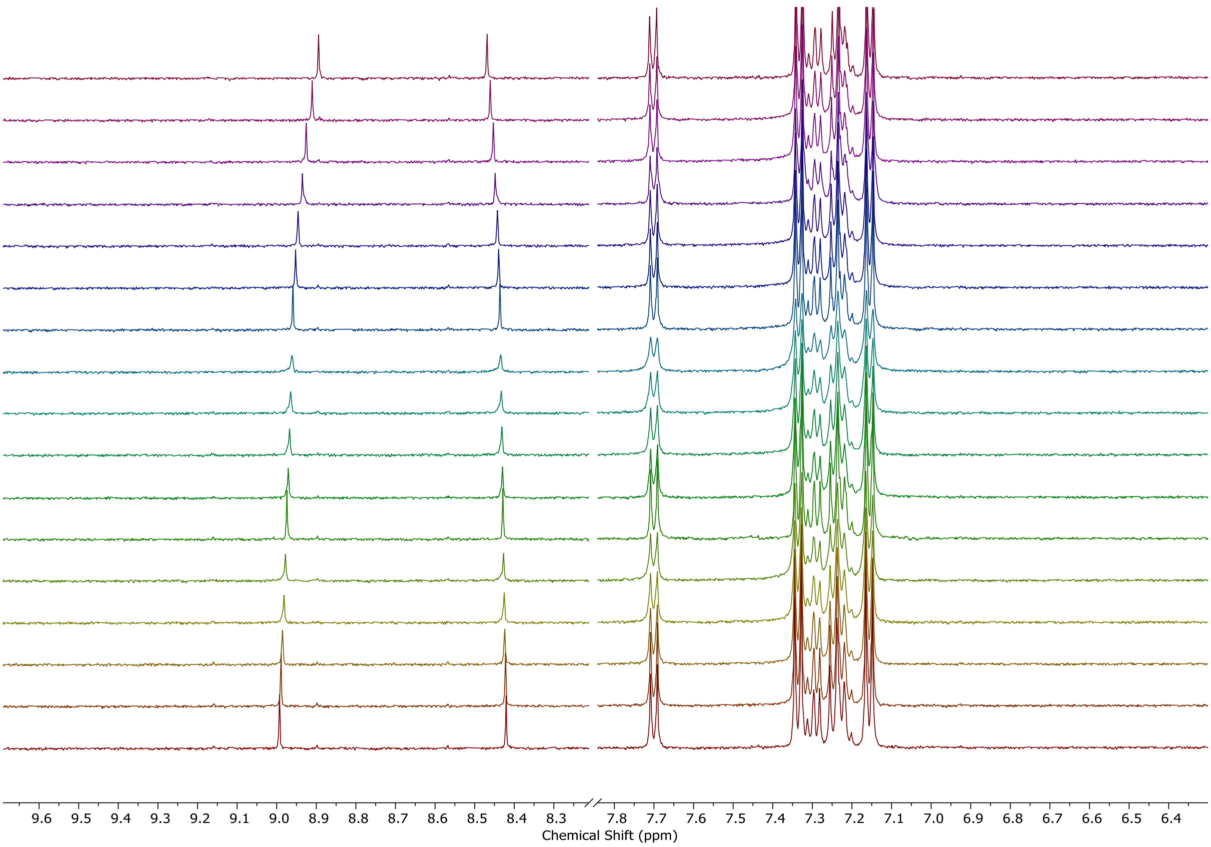


Figure S67. Stacked $^{1}H$ NMR TBACl titration of **2·XB$^{(NO2)2}$** in $D_2O$:Acetone-$d_6$ (10:90, v/v), 500 MHz, 298 K.

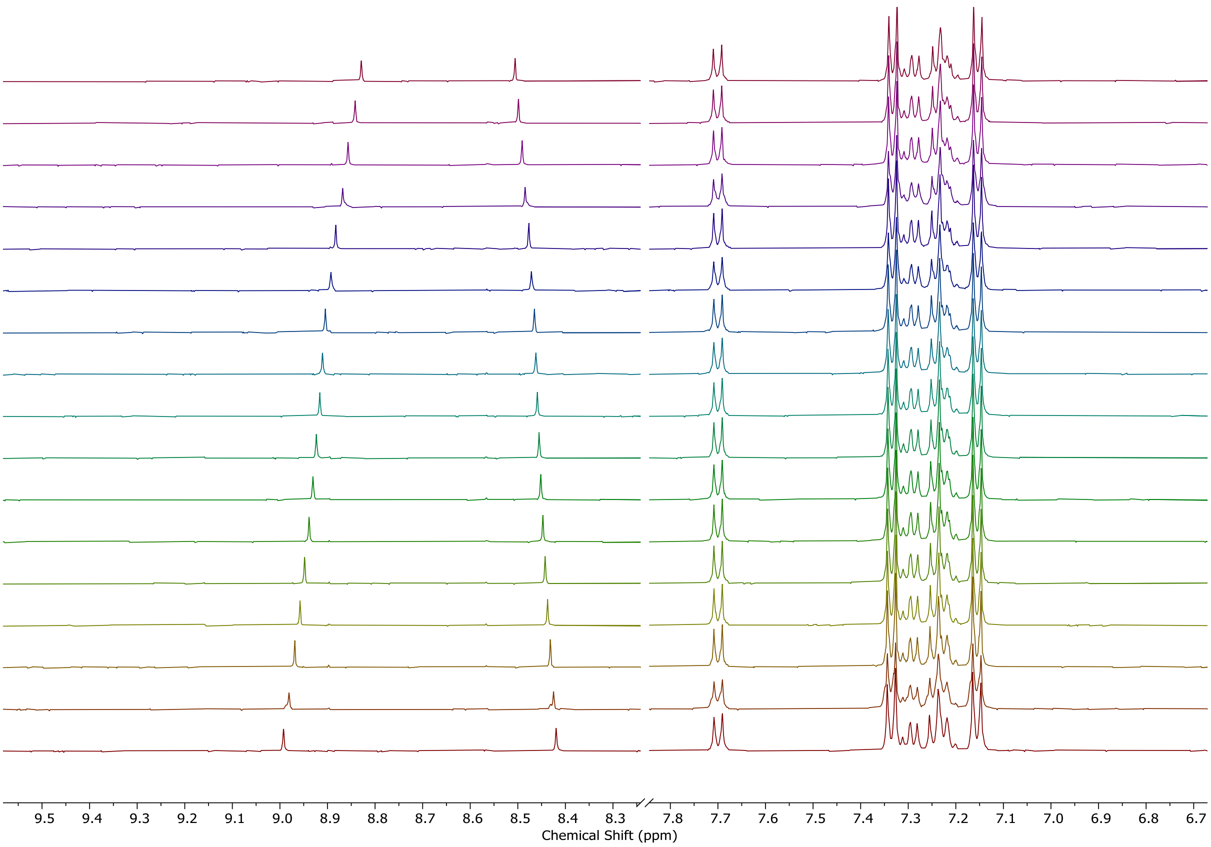


Figure S68. Stacked $^{1}H$ NMR TBABr titration of **2·XB$^{(NO2)2}$** in $D_2O$:Acetone-$d_6$ (10:90, v/v), 500 MHz, 298 K.

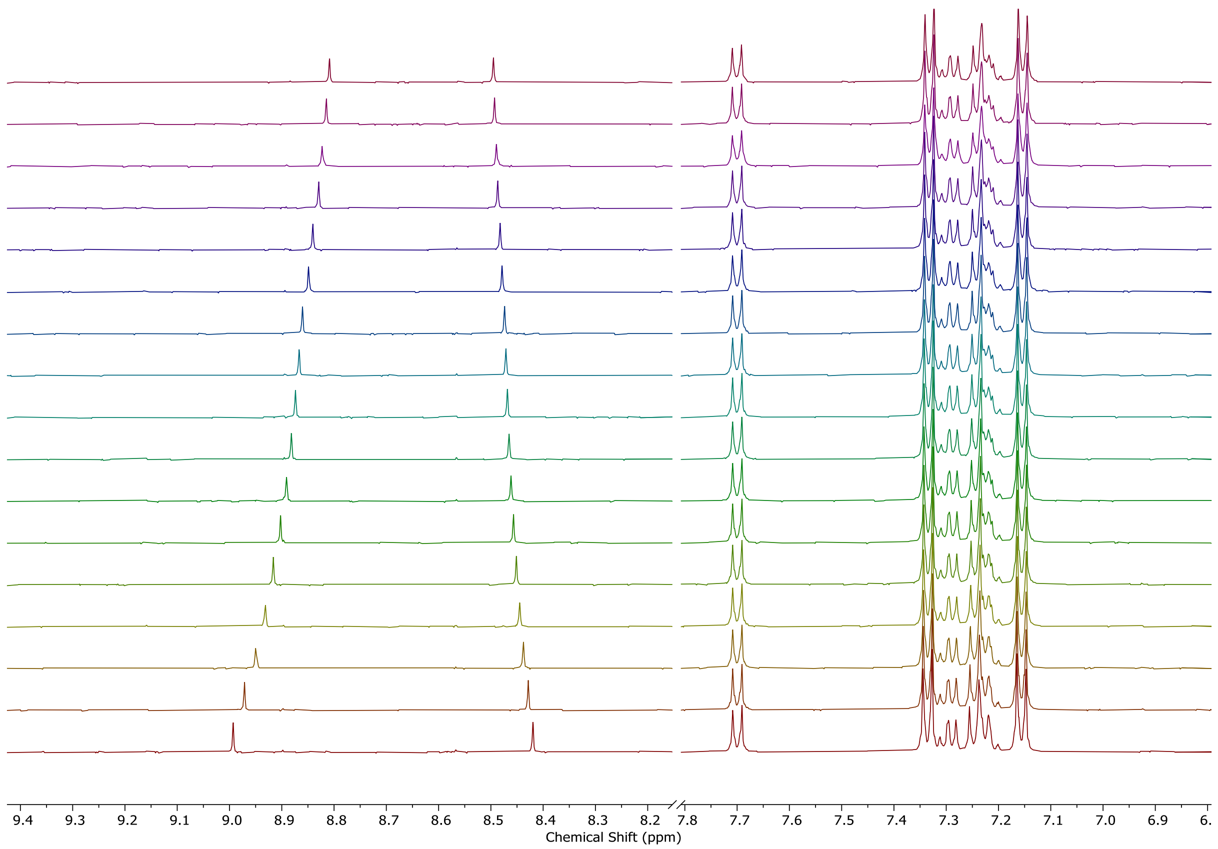


Figure S69. Stacked $^1$H NMR TBAI titration of **2·XB$^{(NO2)2}$** in $D_2O$:Acetone-$d_6$ (10:90, v/v), 500 MHz, 298 K.

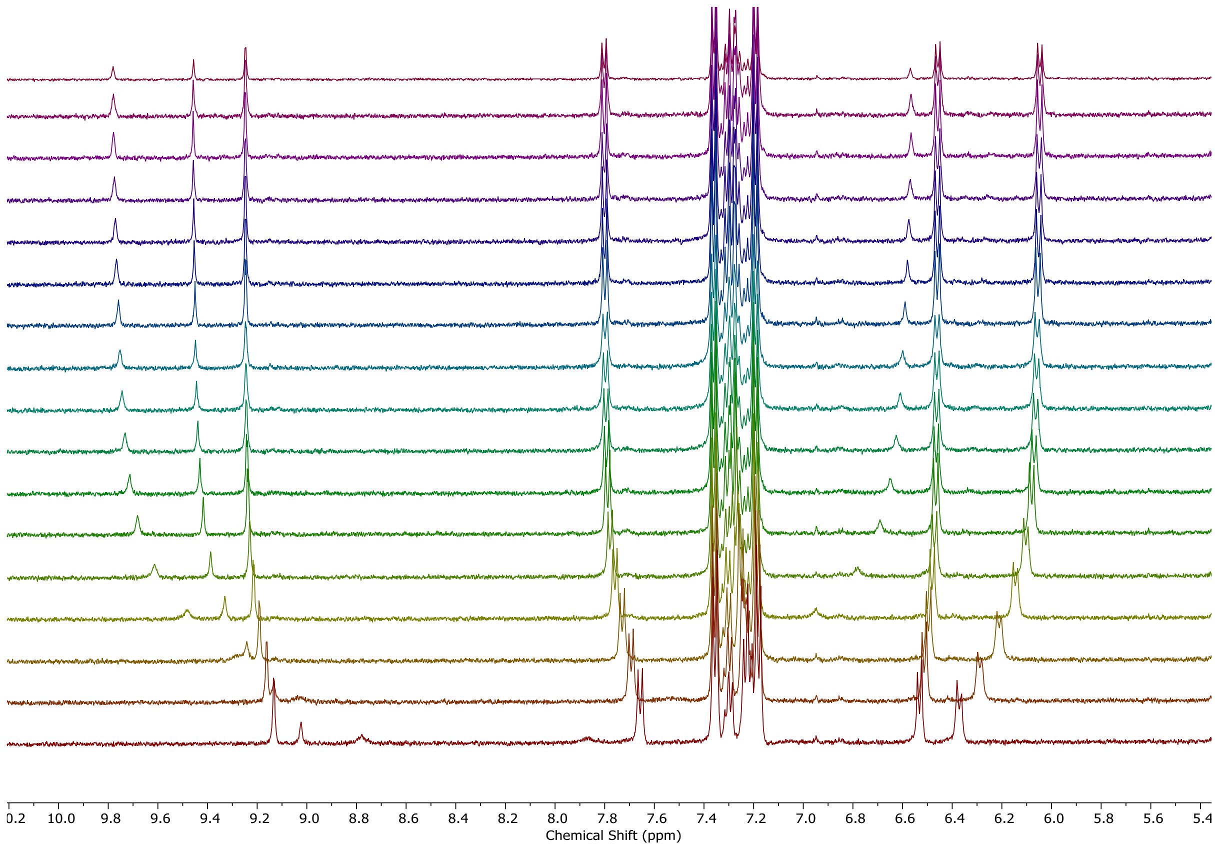


Figure S70. Stacked $^{1}$H NMR TBACl titration of **3·XB$^{(NO2)2}$** in $D_2O$:Acetone-$d_6$ (5:95, v/v), 500 MHz, 298 K.

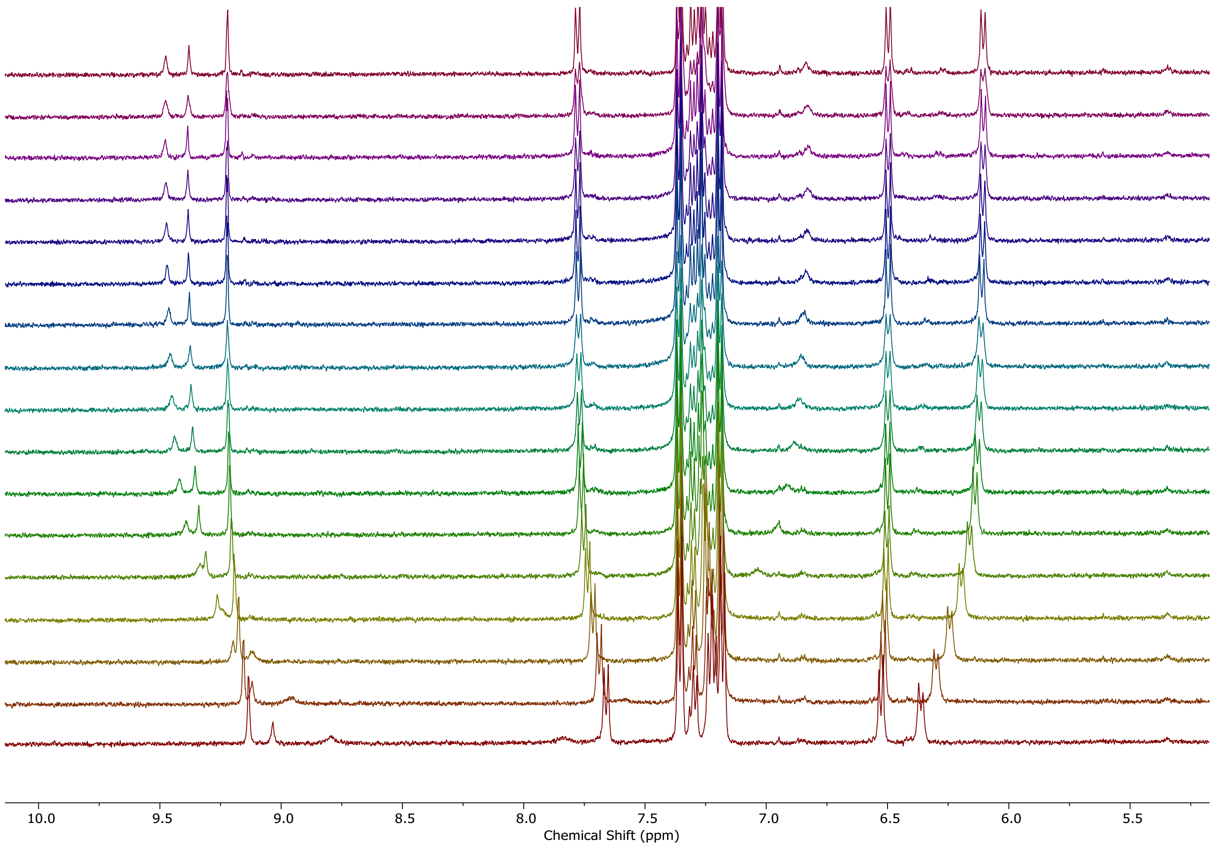


Figure S71. Stacked $^{1}H$ NMR TBABr titration of **3·XB$^{(NO2)2}$** in $D_2O$:Acetone-$d_6$ (5:95, v/v), 500 MHz, 298 K.

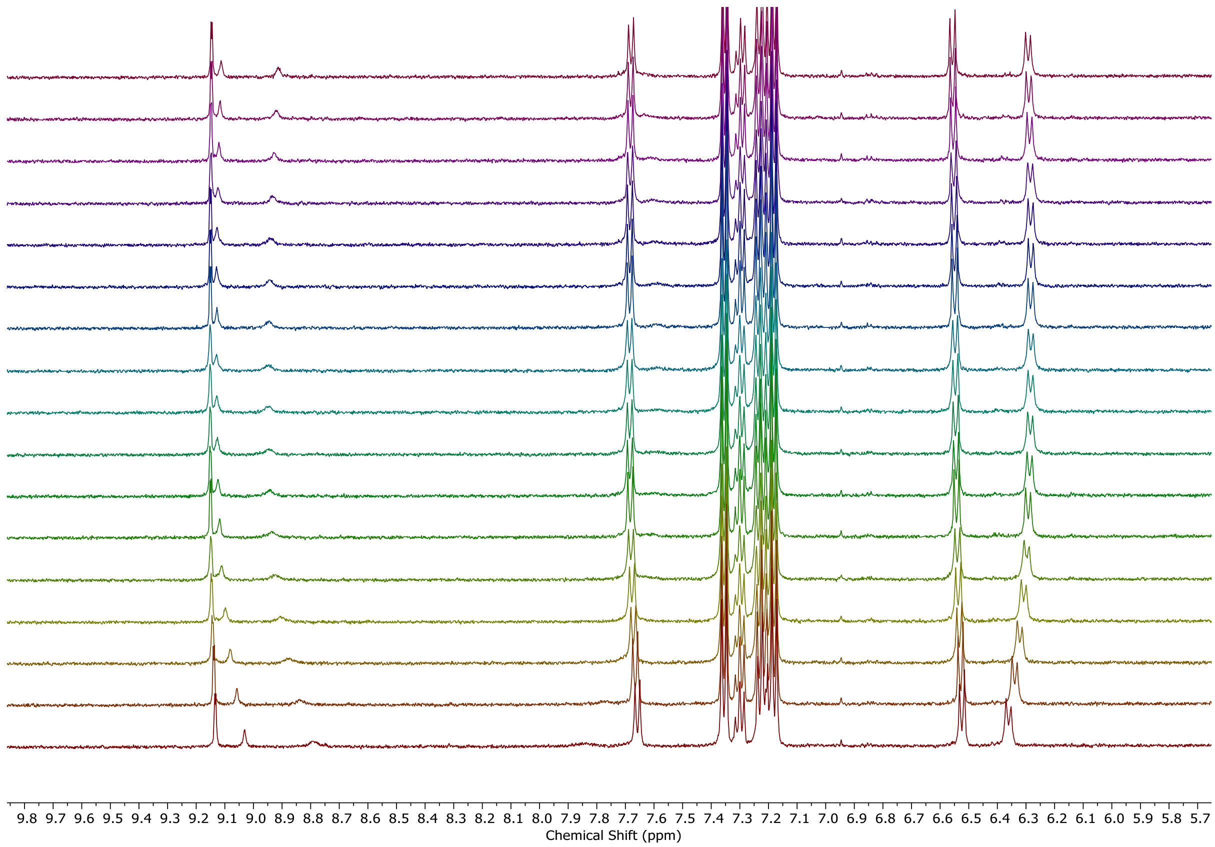


Figure S72. Stacked $^{1}H$ NMR TBAI titration of **3·XB$^{(NO2)2}$** in $D_2O$:Acetone-$d_6$ (5:95, v/v), 500 MHz, 298 K.

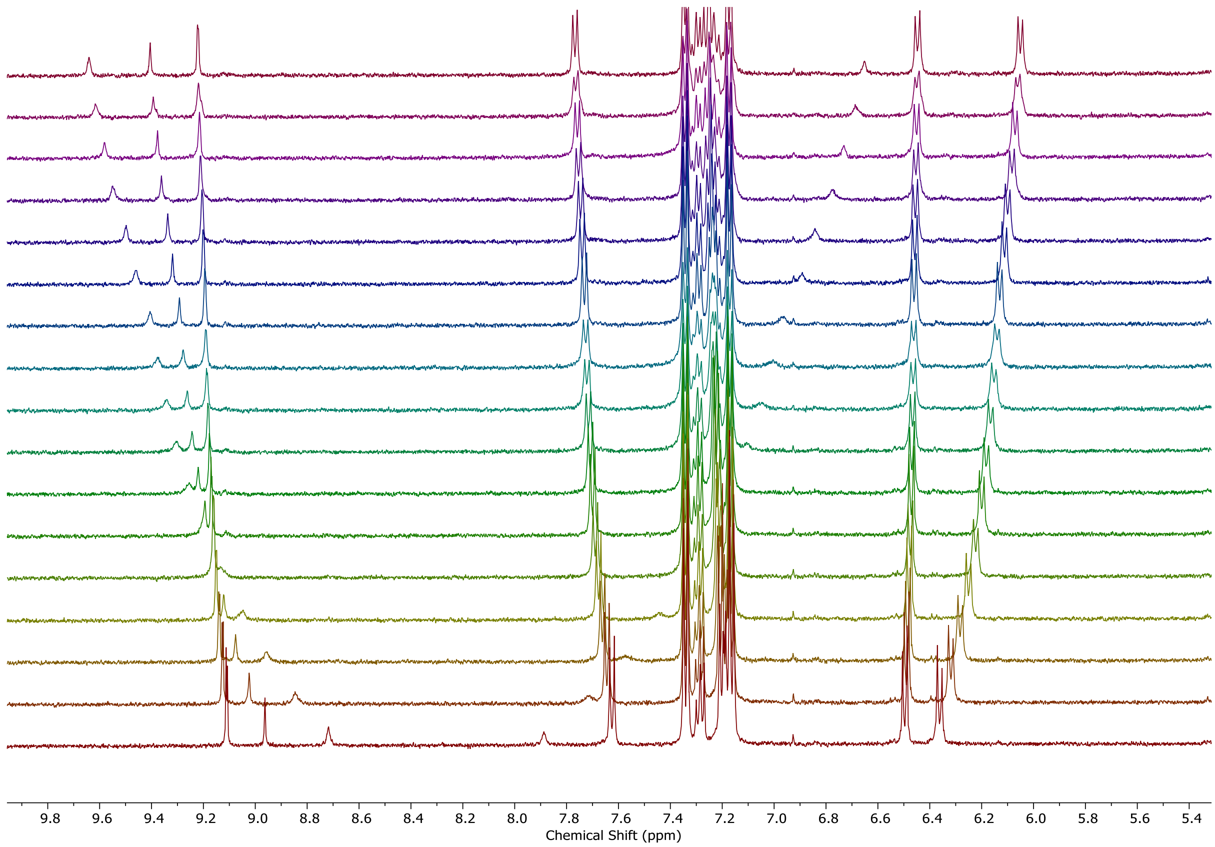


Figure S73. Stacked $^{1}H$ NMR TBACl titration of **3·XB$^{(NO2)2}$** in $D_2O$:Acetone-$d_6$ (10:90, v/v), 500 MHz, 298 K.

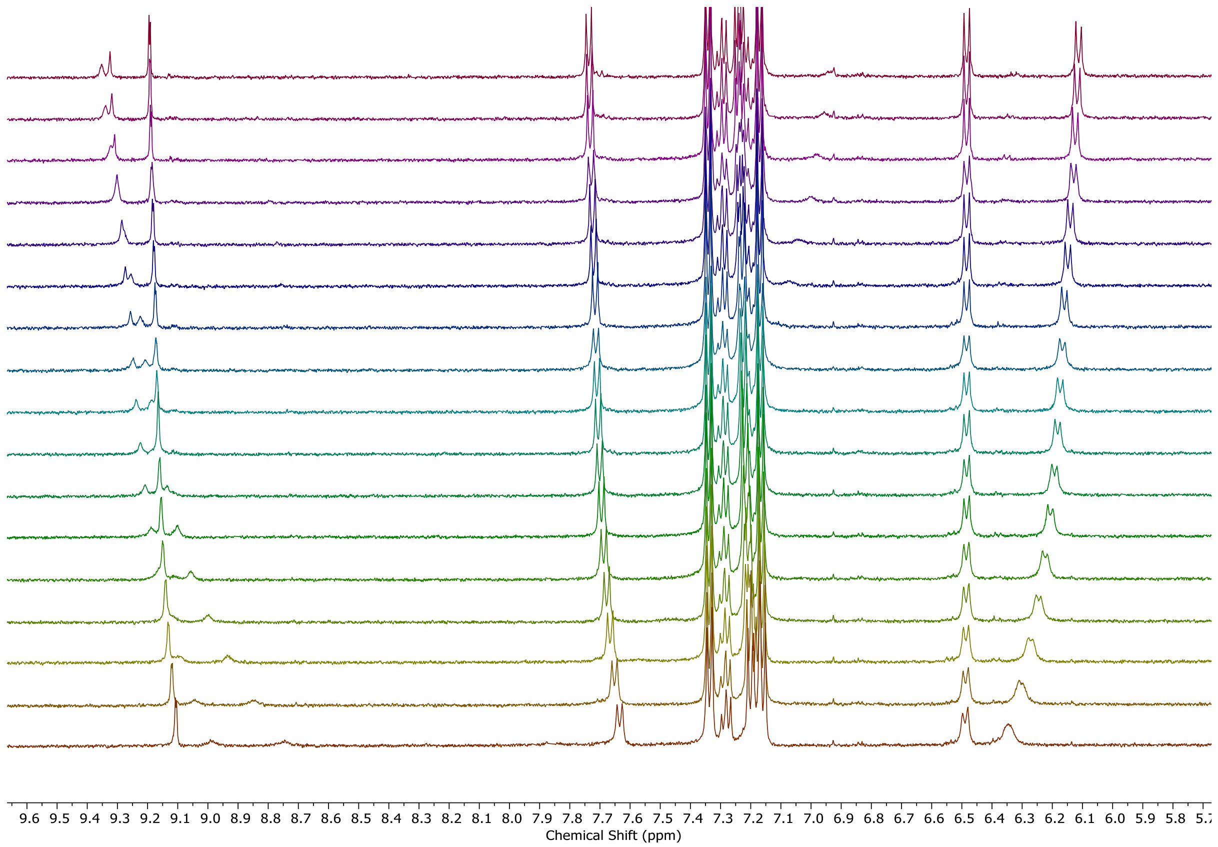


Figure S74. Stacked $^{1}$H NMR TBABr titration of **3·XB$^{(NO2)2}$** in $D_2O$:Acetone-$d_6$ (10:90, v/v), 500 MHz, 298 K.

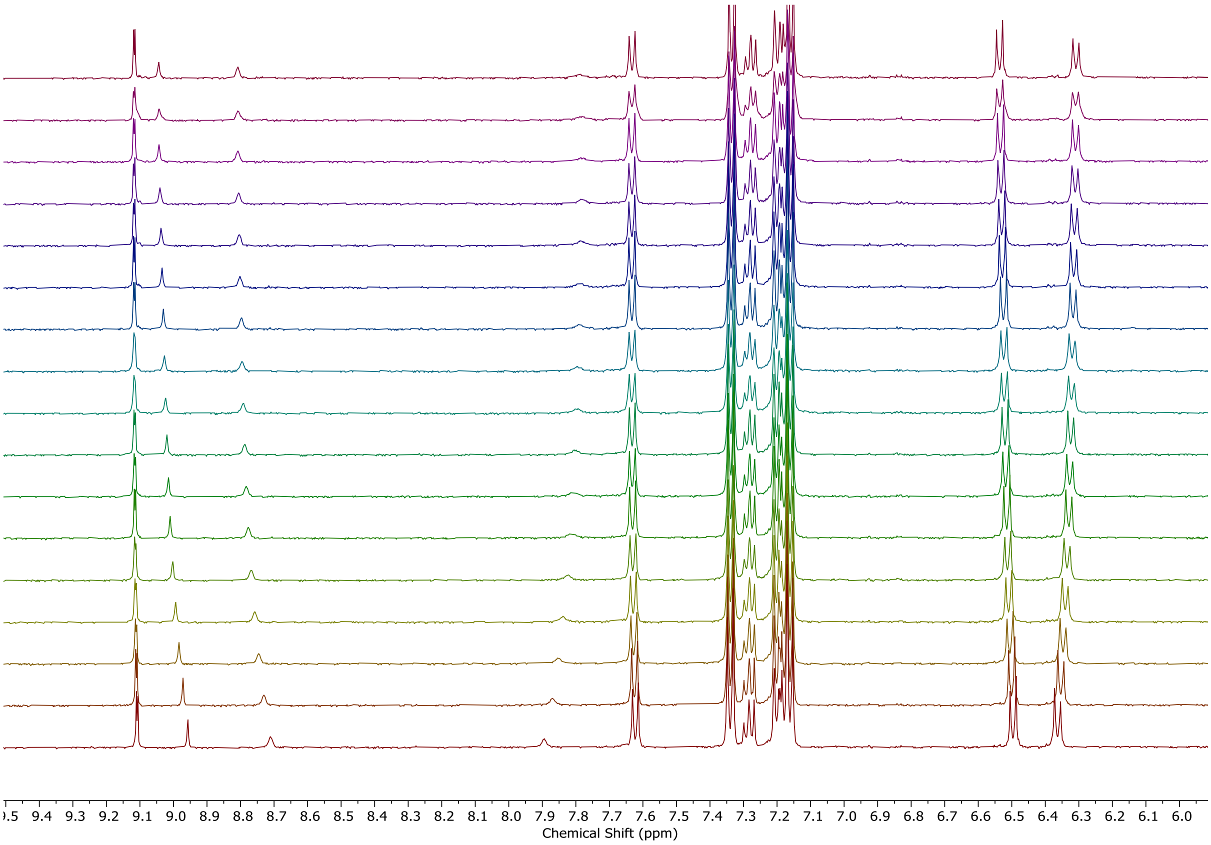


Figure S75. Stacked $^{1}H$ NMR TBAI titration of **3·XB$^{(NO2)2}$** in $D_2O$:Acetone-$d_6$ (10:90, v/v), 500 MHz, 298 K.

## 4. 2D ¹H-¹H ROESY NMR Spectra

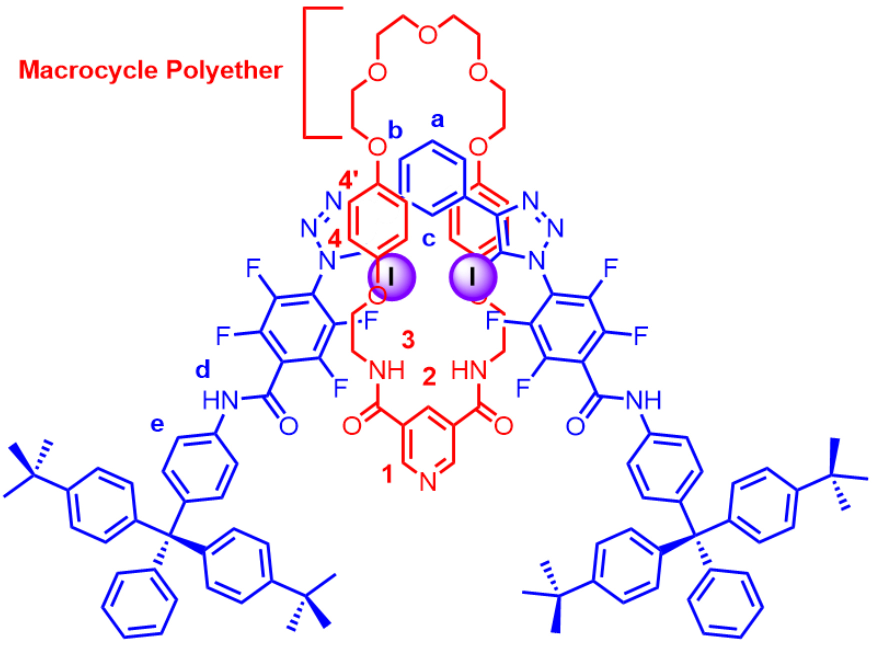


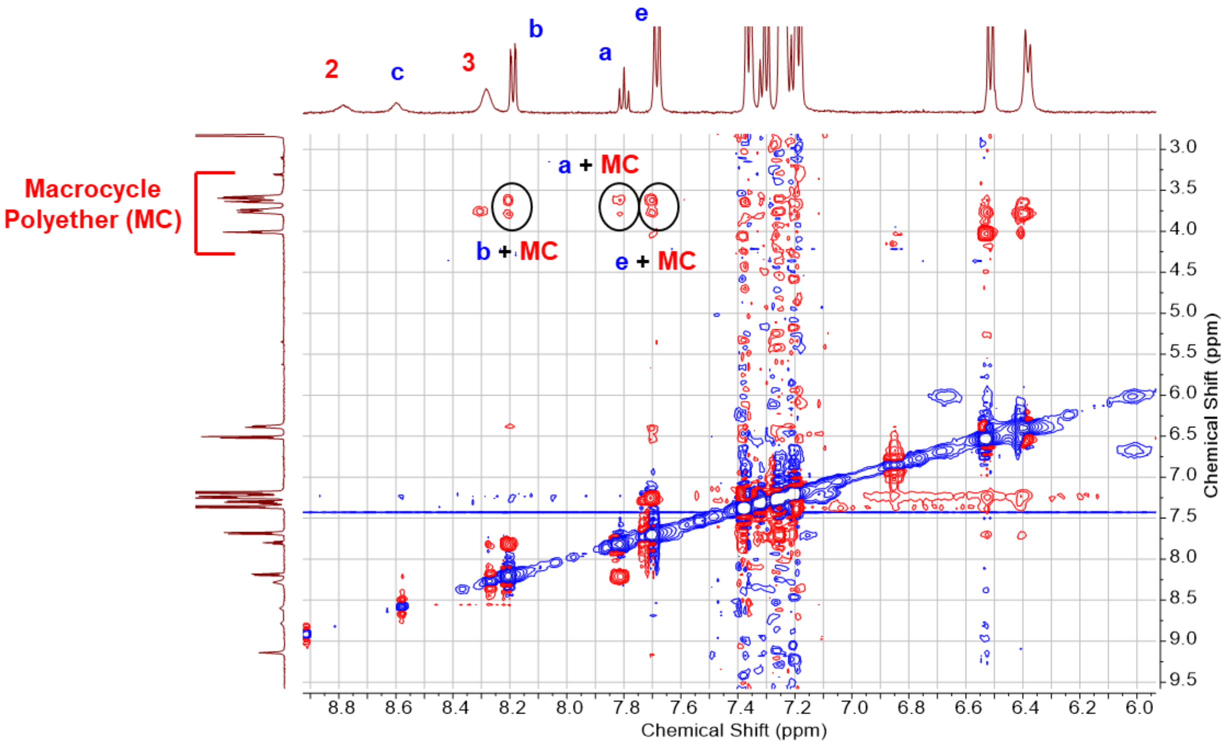


Figure S76. 2D $^1$H-$^1$H ROESY NMR of **3·XB** (acetone-d$_6$, 298 K, 500 MHz).

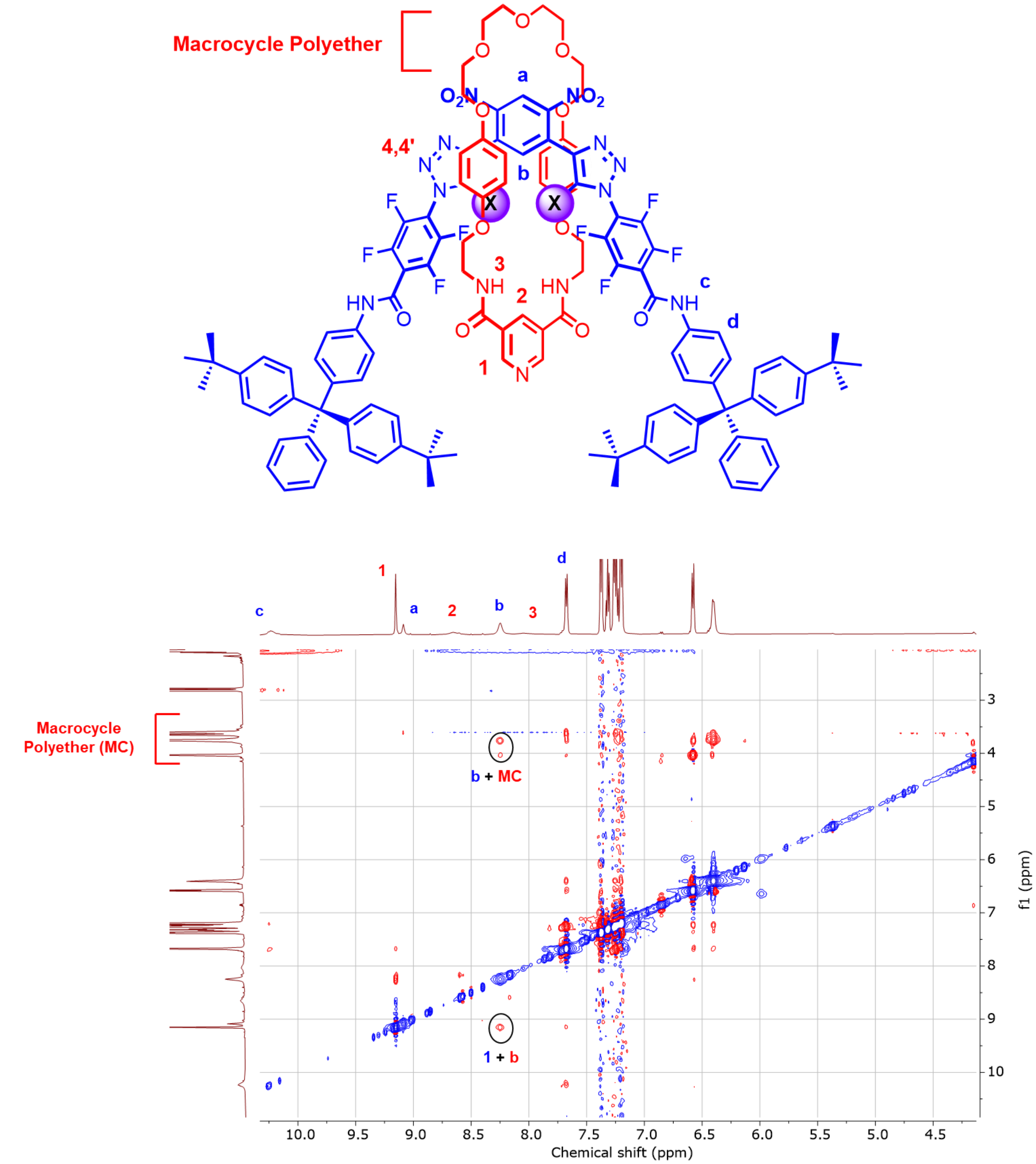


Figure S77. 2D $^{1}H$-$^{1}H$ ROESY NMR of **3·XB$^{(NO2)2}$** (acetone-$d_6$, 298 K, 500 MHz).

## 5. Single Crystal Diffraction Data

Single crystals of **1·XB$^{(NO2)2}$**·TBACl suitable for X-ray analysis were each coated with Paratone-N oil, suspended on a 200 μm MiTeGen loop, and placed in a cold gaseous nitrogen stream on an Oxford Diffraction Supernova X-ray diffractometer performing φ- and ω-scans at 150(2) K. Diffraction intensities were measured using graphite monochromated Cu Kα radiation (1.54184 Å). Data collection, indexing, initial cell refinements, frame integration, final cell refinements and absorption corrections were accomplished using the program CrysAlisPro. † Scattering factors and anomalous dispersion corrections were taken from the International Tables for X-ray Crystallography. The structure was solved by direct methods and refined against F2. All hydrogen atoms were included into the model at geometrically calculated positions and refined using a riding model. Figures of the crystal structures have been created using the open-source PyMOL Molecular Graphics System. Selected crystallographic data for **1·XB$^{(NO2)2}$**·TBACl is given in Table S3. Crystal samples of compound **1·XB$^{(NO2)2}$**·TBACl were twined and the structure was modelled using the HKLF5 refinement against data that accounted for the twinning rotation matrix.‡ In the crystal lattice, disordered solvent molecules were modelled with Squeeze procedure in Platon. ‡

Deposition Number; 2081712 for **1·XB$^{(NO2)2}$**·TBACl contains the supplementary crystallographic data for this paper. These data are provided free of charge by the joint Cambridge Crystallographic Data Centre and Fachinformationszentrum Karlsruhe Access Structures service www.ccdc.cam.ac.uk/structures.

† CrysAlisPRO, Oxford Diffraction /Agilent Technologies UK Ltd, Yarnton, England.

‡ Single-crystal structure validation with the program PLATON. Spek, A. L. Journal of Applied Crystallography (2003), 36 (1), 7-13.

Table S3. Crystal data and structure refinement for **1·XB$^{(NO2)2}$**·TBACl.

| | | |
|---|---|---|
| Identification code | 006zy21 | |
| Empirical formula | C38 H38 Cl F10 I2 N9 O4 | |
| Formula weight | 1164.02 | |
| Temperature | 150(2) K | |
| Wavelength | 1.54184 Å | |
| Crystal system | Triclinic | |
| Space group | P-1 | |
| Unit cell dimensions | a = 13.5191(4) Å | α= 97.386(2)°. |
| | b = 15.5677(6) Å | β= 90.098(2)°. |
| | c = 27.2235(6) Å | γ = 113.512(3)°. |
| Volume | 5201.4(3) $Å^3$ | |
| Z | 4 | |
| Density (calculated) | 1.486 $Mg/m^3$ | |
| Absorption coefficient | 10.677 $mm^{-1}$ | |
| F(000) | 2296 | |
| Crystal size | 0.4 x 0.2 x 0.1 $mm^3$ | |
| Theta range for data collection | 3.571 to 79.217°. | |
| Index ranges | -17<=h<=17, -19<=k<=19, -34<=l<=34 | |
| Reflections collected | 41347 | |
| Independent reflections | 41347 [R(int) = 0.1206] | |
| Completeness to theta = 67.684° | 100.0 % | |
| Absorption correction | Semi-empirical from equivalents | |
| Max. and min. transmission | 1.00000 and 0.62082 | |
| Refinement method | Full-matrix least-squares on $F^2$ | |
| Data / restraints / parameters | 41347 / 0 / 1161 | |
| Goodness-of-fit on $F^2$ | 0.928 | |
| Final R indices [I>2sigma(I)] | R1 = 0.0863, wR2 = 0.2165 | |
| R indices (all data) | R1 = 0.1330, wR2 = 0.2445 | |
| Extinction coefficient | n/a | |
| Largest diff. peak and hole | 3.513 and -1.608 e.$Å^{-3}$ | |

## 6. References


[1] L. M. Hancock, L. C. Gilday, S. Carvalho, P. J. Costa, V. Félix, C. J. Serpell, N. L. Kilah, P. D. Beer, *Chem. – Eur. J.* **2010**, *16*, 13082–13094.

[2] J. F. W. Keana, S. X. Cai, *J. Org. Chem.* **1990**, *55*, 3640–3647.

[3] A. Borissov, J. Y. C. Lim, A. Brown, K. E. Christensen, A. L. Thompson, M. D. Smith, P. D. Beer, *Chem. Commun.* **2017**, *53*, 2483–2486.

[4] J. M. Mahoney, K. A. Stucker, H. Jiang, I. Carmichael, N. R. Brinkmann, A. M. Beatty, B. C. Noll, B. D. Smith, *J. Am. Chem. Soc.* **2005**, *127*, 2922–2928.

[5] B. Qiao, J. R. Anderson, M. Pink, A. H. Flood, *Chem. Commun.* **2016**, *52*, 8683–8686.
